# A mathematical model of metabolism and regulation provides a systems-level view of how *Escherichia coli* responds to oxygen

**DOI:** 10.3389/fmicb.2014.00124

**Published:** 2014-03-27

**Authors:** Michael Ederer, Sonja Steinsiek, Stefan Stagge, Matthew D. Rolfe, Alexander Ter Beek, David Knies, M. Joost Teixeira de Mattos, Thomas Sauter, Jeffrey Green, Robert K. Poole, Katja Bettenbrock, Oliver Sawodny

**Affiliations:** ^1^Institute for System Dynamics, University of StuttgartStuttgart, Germany; ^2^Max Planck Institute for Dynamics of Complex Technical SystemsMagdeburg, Germany; ^3^Department of Molecular Biology and Biotechnology, The University of SheffieldSheffield, UK; ^4^Molecular Microbial Physiology, Swammerdam Institute for Life Sciences, University of AmsterdamAmsterdam, Netherlands; ^5^Life Sciences Research Unit, Université du LuxembourgLuxembourg, Luxembourg

**Keywords:** *Escherichia coli*, mathematical modeling, metabolism, regulation, respiration, fermentation, thermokinetic modeling

## Abstract

The efficient redesign of bacteria for biotechnological purposes, such as biofuel production, waste disposal or specific biocatalytic functions, requires a quantitative systems-level understanding of energy supply, carbon, and redox metabolism. The measurement of transcript levels, metabolite concentrations and metabolic fluxes *per se* gives an incomplete picture. An appreciation of the interdependencies between the different measurement values is essential for systems-level understanding. Mathematical modeling has the potential to provide a coherent and quantitative description of the interplay between gene expression, metabolite concentrations, and metabolic fluxes. *Escherichia coli* undergoes major adaptations in central metabolism when the availability of oxygen changes. Thus, an integrated description of the oxygen response provides a benchmark of our understanding of carbon, energy, and redox metabolism. We present the first comprehensive model of the central metabolism of *E. coli* that describes steady-state metabolism at different levels of oxygen availability. Variables of the model are metabolite concentrations, gene expression levels, transcription factor activities, metabolic fluxes, and biomass concentration. We analyze the model with respect to the production capabilities of central metabolism of *E. coli*. In particular, we predict how precursor and biomass concentration are affected by product formation.

## 1. Introduction

*Escherichia coli* is able to utilize a variety of electron and carbon donors, such as glucose or glycerol, and electron acceptors, such as oxygen or nitrate. Energy currencies in form of the proton motive force (pmf) and the ATP/ADP ratio are supplied by either substrate level phosphorylation or by proton translocation against the pmf during membrane-associated electron transport (Lengeler et al., 1998). The membrane-associated electron transport chain transfers electrons from cytoplasmatic metabolites, mostly the electron carriers NADH and FADH_2_, to quinones and from the quinones to the external electron acceptor (Ingledew and Poole, 1984). The thermodynamic force of these redox reactions can be used to translocate protons and thus contribute to the maintenance of the pmf. Dependent on the extracellular medium, *E. coli* uses different strategies to match the balance of carbon, electrons, and energy. We focus on the use of glucose as electron and carbon donor and oxygen as the electron acceptor. If oxygen is available, the redox balance is maintained by transferring electrons to the external acceptor oxygen. Membrane-associated electron transfer is coupled to proton translocation. Glucose is oxidized to carbon dioxide or partially oxidized products such as acetate and succinate, often referred to as overflow products, or it is converted into precursors for biosynthesis. If no oxygen and no other external electron acceptors are available, ATP is gained mainly by substrate level phosphorylation in glycolysis. Redox balance is maintained by the excretion of different metabolites (e.g., acetate, formate, ethanol, and succinate). Even in the absence of extracellular electron acceptors, the electron transport chain can be active, since intracellular electron acceptors, such as fumarate, are available (Lengeler et al., 1998).

The metabolism of bacteria needs to match the requirements of growth and maintenance for carbon, electrons and energy with the supply from the medium. Complex regulatory networks control this process, such as those that operate following a change in oxygen availability. The redesign of bacteria for biotechnological purposes, such as biofuel production, puts additional loads on the metabolism. A high level expression of a production pathway is often not sufficient for satisfactory production capabilities. For optimal production, metabolic regulation needs to be adapted accordingly (Shimizu, 2009).

The adaption to a change of oxygen availability is controlled by transcriptional regulation centered around the transcription factors FNR and ArcA (Sawers, 1999). The activity of FNR depends directly on oxygen (Jordan et al., 1997) and the activity of ArcA depends on the quinols and quinones of the electron transport chain (Georgellis et al., 1997; Bekker et al., 2010; Alvarez et al., 2013; Sharma et al., 2013).

Alexeeva et al. (2000, 2002, 2003) introduced the aerobiosis scale that allows the reproducible adjustment of different microaerobic steady states in a continuous, glucose-limited chemostat culture. Anaerobic growth corresponds to an aerobiosis value of 0%. An aerobiosis value of 100% is defined as the steady state with the minimal oxygen input into the reactor where no fermentation products are excreted, i.e., where all carbon is either incorporated into biomass or respired to carbon dioxide. The aerobiosis scale is linear with the oxygen input into the reactor. The dependency of the biomass-specific oxygen uptake flux on the aerobiosis level is concave because at higher oxygen availability the steady state biomass concentration is higher. For the *E. coli* wild type, biomass-specific acetate production decreases linearly with aerobiosis until it vanishes at 100% aerobiosis units. The aerobiosis scale allows the reproducible analysis of microaerobic states on the physiological (Alexeeva et al., 2000, 2002, 2003; Bekker et al., 2010; Steinsiek et al., 2011) and transcriptional level (Partridge et al., 2006, 2007; Rolfe et al., 2011, 2012; Trotter et al., 2011). Using the aerobiosis scale, results of different laboratories using different reactors can be compared. The aerobiosis scale thus provides an ideal basis for mathematical modeling.

The analysis of measurement data of transcript levels, protein abundances, metabolite concentrations, and fluxes is a valuable tool to reveal bottlenecks of production pathways. In simple cases, such as a feedback inhibition in a linear path, the repressing conditions can be identified by the measurement of metabolite concentrations and reaction fluxes, and countermeasures can be implemented genetically. In more complex cases, the inherent correlation between different measured quantities may not always be so apparent. The metabolic pathways of the central metabolism form a strongly interconnected network with complex interdependencies. A thorough analysis of manipulations of this network requires integration of measurement data of different types, in particular transcript and protein levels, metabolite concentrations, fluxes, and transcription factor activities, by analyzing their dependencies within the network structure. Mathematical modeling of the central metabolism can provide tools for analyzing and predicting the effect of genetic intervention and thus provide guidance when redesigning organisms for biofuel production. The model-based integration of signal transduction, regulation, and metabolism is still not standard and most models are restricted to describing either metabolic processes, signal transduction or regulation (Gonçalves et al., 2013). The response of cellular metabolism to oxygen was studied previously using mathematical models. For example, Varma et al. (1993) used flux balance analysis to analyze the yield optimal behavior of *E. coli* for different oxygen availabilities. Peercy et al. (2006) presented a kinetic model of the respiratory chain of *E. coli* and its regulation via FNR and ArcA. They demonstrated that the model is able to show complex dynamic behavior such as oscillations and hysteresis. Beard (2005) described the electron transport chain of mitochondria by choosing a force for each reaction that is consistent with the requirements of thermodynamics. Similarly, Klamt et al. (2008) used linear relationships of affinity and flux to describe the kinetics of the electron transport chain of purple non-sulfur bacteria.

Here, we present a modeling approach that integrates several levels of information. The goal of the modeling approach was to provide a physically consistent systems-level view of the central carbon and energy metabolism of *E. coli* and its regulation. We show how the model is able to explain the steady state response of *Escherichia coli* to oxygen by comparing model simulation and measurement data for different values of aerobiosis. We demonstrate the utility of the model by making predictions on the effect of biofuel production pathways on bacterial metabolism.

## 2. Materials and methods

### 2.1. Modeling

The complete model information is available in the supplementary files. In particular, Supplementary Data Sheet 1 shows an overview of all model elements in alphabetical order and Supplementary Data Sheet 2 shows the model definition file. The model definition file together with the Mathematica package TKMOD (Thermo-Kinetic Modeling) can be downloaded from https://seek.sysmo-db.org/models/23. Together they provide a runnable version of the model.

#### 2.1.1. Model of the metabolism

We use the thermodynamic-kinetic modeling formalism (Ederer and Gilles, 2007; Ederer, 2010) to describe the metabolic reaction network. We assume an ideal, aqueous solution with the chemical potentials *μ*″_*i*_ = *μ*″°_*i*_ (*T, p, a*_H_2_O_, pH, *I*) + R^*^ · *T* · log(*c*_*i*_/*c*°) + *z*_*i*_ · *F* · *ϕ*_*i*_. For *μ*″°_*i*_ we use the transformed Gibbs formation energy of metabolite *i*. A Legendre transformation is conducted to adapt the Gibbs energy to constant pH and water activity *a*_H_2_O_ (Alberty, 2003). The Debye-Hückel equation is used to correct for the effect of ionic strength *I* (Alberty, 2003). Temperature *T* and pressure *p* are constant. The symbols R^*^ and *F* denote the ideal gas constant and the Faraday constant, respectively. The charge number *z*_*i*_ of biochemical species *i* is approximated by the charge of the dominant species at pH 7 and taken from Reed et al. (2003). The electrical potential of the compartment of metabolite *i* is denoted by *ϕ*_*i*_. According to Ederer (2010), we get that the relationship between thermokinetic potential ξ_*i*_ and concentration *c*_*i*_ is *c*_*i*_ = *C*_*i*_ · ξ_*i*_ with *C*_*i*_ = *c*° · exp (− (*μ*″°_*i*_ + *z*_*i*_ · *F* · *ϕ*_*i*_)/(R^*^ · *T*)) where *C*_*i*_ is the thermokinetic capacity.

Fluxes *J*_*k*_ of metabolic reactions are modeled according to Ederer (2010) as (*R*_*j*_(ξ)/*c*_E,*j*_) · *J*_*j*_ = *F*_*j*_(ξ) where *F*_*j*_(ξ) is the thermokinetic force, *R*_*j*_(ξ) is the enzyme-specific thermokinetic resistance and *c*_E,*j*_ is the enzyme concentration. For example, the thermokinetic force of reaction *A* + *B* ⇌ *C* is *F* = ξ_*A*_ · ξ_*B*_ − ξ_*C*_. The above expression reflects three major effects that control metabolic fluxes: the thermokinetic force *F* describes the influence of reactants and products, the resistance *R*_*j*_(ξ) describes the specific enzyme activity that may depend on further activators and inhibitors, and the enzyme concentration *c*_E,*j*_ describes the influence of the enzyme concentration on the metabolic reaction. For most reactions the thermokinetic resistance *R*_*j*_(ξ) is assumed to be independent of the metabolite potentials ξ. For some reactions, where enzymatic regulation proved to be important for describing the experimental data according non-constant terms were included in *R*_*j*_(ξ) (see Supplementary Data Sheet 2).

#### 2.1.2. *De novo* synthesis of conserved moieties

The *de novo* synthesis of AMP, NAD, NADP, and CoA was modeled such that the concentrations of these conserved moieties are constant despite dilution due to growth. The *de novo* synthesis of quinones is modeled as a function of the aerobiosis value in order to reproduce the observed changes in the total quinone concentrations. In the model, the pool concentration of ubiquinone and ubiquinol increases linearly with aerobiosis and the pool concentration of menaquinone and menaquinol decreases linearly with aerobiosis. At oxygenation levels higher than 100% aerobiosis these concentrations are constant. In order to reproduce the observation that even in the complete anaerobic case a substantial part of the quinone pool is oxidized, we introduced a constant pool of oxidized quinones that does not participate in any reaction.

#### 2.1.3. Transcription factor activity

Transcription factors control gene expression by activating or repressing the expression of many genes. The observed transcription factor activities are the result of a complex interplay of the amount of the transcription factor that in turn may be controlled by other transcription factors and its activation that is often controlled by a metabolic signal, for example oxygen in the case of FNR. As a simplification, we introduce the activity *a*_TF,*i*_ of transcription factor *i*. If the activity of transcription factor *i* is minimal, we write that *a*_TF,*i*_ = 0 and if it is maximal, we write that *a*_TF,*i*_ = 1. We assume a phenomenological Hill type equation $a_{\text{TF},i}=x_{\text{TF},i}^{n_{\text{TF},i}}/\left(x_{\text{TF},i}^{n_{\text{TF},i}}+k_{\text{TF},i}^{n_{\text{TF},i}}\right)$ that describes *a*_TF,*i*_ in dependence on the respective metabolic signal *x*_TF,*i*_. For *n*_TF,*i*_ > 0 transcription factor *i* is activated by its metabolic signal *x*_TF,*i*_. For *n*_TF,*i*_ < 0 it is inhibited. Supplementary Data Sheet 3 lists the transcription factors with the respective metabolic signals.

#### 2.1.4. Gene expression

Gene expression is described by the equation *ċ*_E,*i*_ = *J*_syn,*i*_(*a*_TF_) − *μ* · *c*_E,*i*_ where *c*_E,*i*_ is the concentration of enzyme *i*, *μ* is the dilution rate due to growth and *J*_syn,*i*_ is the synthesis rate of enzyme *i* that depends on the activities of the transcription factors *a*_TF_. The complex dependency of the synthesis rate on the transcription factors is approximated by a phenomenological relationship. For example, the expression of a gene that is activated by transcription factors 1 and 2 and inhibited by transcription factor 3 is modeled by *J*_syn,*i*_ = *s*(*k*_1_, *a*_TF,1_) · *s*(*k*_2_, *a*_TF,2_) · *s*(*k*_3_, 1 − *a*_TF,3_) where *s*(*k, a*_*TF*_) = 2^−*k*^ + (1 − 2^−*k*^) · *a*_*TF*_. This means that each transcription factor is able to change the expression of a gene by a factor of 2^*k*^ and the interaction of different transcription factors is multiplicative. As will be seen, this model allows the description of the measurement data and therefore the use of more complex models allowing for example for additive interactions is not necessary at this stage.

#### 2.1.5. Growth and maintenance

Central metabolism provides biosynthesis with precursor molecules and with redox and energy equivalents. In order to describe the growth of the biomass we assume a stoichiometric relation for the reaction of precursors into biomass:

$$\begin{array}{c} \begin{array}{c} \nu_{\text{g6p}}\cdot \text{G6P}+\nu_{\text{f6p}}\cdot \text{F6P}+\nu_{\text{dhap}}\cdot \text{DHAP}+\nu_{\text{3pg}}\cdot \text{3PG}+\\ \nu_{\text{pep}}\cdot \text{PEP}+\nu_{\text{pyr}}\cdot \text{PYR}+\nu_{\text{accoa}}\cdot \text{ACCOA}+\nu_{\text{succoa}}\cdot \text{SUCCOA}+\\ \nu_{\text{akg}}\cdot \text{AKG}+\nu_{\text{oaa}}\cdot \text{OAA}+\nu_{\text{r5p}}\cdot \text{R5P}+\nu_{\text{e4p}}\cdot \text{E4P}+\\ \nu_{\text{atp}}\cdot \text{ATP}+\nu_{\text{nadph}}\cdot \text{NADPH}+\nu_{\text{nad}}\cdot \text{NAD}\\ \to \\ (\nu_{\text{accoa}}+\nu_{\text{succoa}})\cdot \text{COA}+\nu_{\text{atp}}\cdot \text{ADP}+\nu_{\text{nad}}\cdot \text{NADH}+\\ \nu_{\text{nadph}}\cdot \text{NADP}+\nu_{\text{g3p}}\cdot \text{G3P}+\nu_{\text{succ}}\cdot \text{SUCC}+\nu_{\text{fum}}\cdot \text{FUM}+\\ \nu_{\text{co2}}\cdot \text{CO2}+\nu_{\text{ac}}\cdot \text{AC}+1\text{g DCW} \end{array} \end{array}$$

where the stoichiometric coefficients ν_*i*_ define how much precursor is needed to produce 1 gram of dry cell mass (see Neidhardt et al., 1990). We assume that the rate of this reaction depends on the concentrations of the reactants with linlog kinetics. Growth of *E. coli* can only occur if the adenylate energy charge is above a threshold (Chapman et al., 1971). To model this fact, the linlog kinetics are extended by a factor realizing a ramp function with saturation dependent on the ATP/ADP ratio:

$$\mu =\{\begin{array}{ll} 0 & \text{if }k_{lo}<c_{atp}/c_{adp}\\ k_{a}\cdot (k_{b}+\sum_{i}\nu_{i}\cdot \log(c_{i}))\cdot & \text{if }k_{lo}\leq c_{atp}/c_{adp}\leq k_{hi}\\ \;(c_{atp}/c_{adp}-k_{lo})/(k_{hi}-k_{lo}) & \\ k_{a}\cdot (k_{b}+\sum_{i}\nu_{i}\cdot \log(c_{i})) & \text{if }k_{hi}<c_{atp}/c_{adp} \end{array}$$

where *i* runs over the reactants. Cellular maintenance is modeled by assuming a hydrolysis rate of ATP to ADP that is not coupled to processes of the central metabolism or growth. It follows a ramp function with saturation dependent on the ATP/ADP ratio. The use of ramp functions assures that below a certain threshold no growth or maintenance occurs and that the rate of growth or maintenance saturates above a certain threshold.

#### 2.1.6. Chemostat environment

With the specific growth rate we get according to the chemostat equations (Smith and Waltman, 2008) for the biomass concentration **ċ**_X_ = *μ* · *c*_X_ − *D* · *c*_X_. The extracellular metabolites are described by **ċ**_*i*_ = *J*_*i*_ · *c*_X_ + *D* · *c*_in,*i*_ − *D* · *c*_*i*_, where *c*_in,*i*_ is the concentration of *i* in the inflow, *J*_*i*_ is the specific production (positive) or consumption (negative) rate of metabolite *i* by the biomass and *D* is the dilution rate. For the gaseous compounds oxygen and carbon dioxide the equation is modified to **ċ**_*i*_ = *J*_*i*_ · *c*_X_ + *k*_in,*i*_ − *k*_out,*i*_ · *c*_*i*_ because this compounds are mainly exchanged via the gas phase, but not the liquid phase. The parameter *k*_in,*i*_ describes the supply of the medium by the aeration flow. For oxygen this parameter depends on the aerobiosis value. The parameter *k*_out,*i*_ describes the outgasing.

#### 2.1.7. Model reduction

The resulting model spans the time scales from fast metabolic reactions up to steady state growth. The stiffness of the model calls for model reduction. For several reactions of the central metabolism it is known that they proceed near thermodynamic equilibrium (Kümmel et al., 2006a). We assume quasi-equilibrium for several reactions. For example the reaction G6P ⇌ F6P is usually rapid such that we can assume that the concentrations of G6P and F6P are in equilibrium with each other. This allows the reduction of the order and the stiffness of the model. In thermokinetic modeling this can be achieved by assuming a vanishing resistance (*R*_*j*_ = 0). The resulting differential-algebraic equation system has index 2 but can be simplified to index 1 (Ederer, 2010).

#### 2.1.8. TKMOD

Modeling is done using the tool TKMOD (Ederer, 2010). TKMOD reads a model description (see Supplementary Data Sheet 2) where stoichiometry, thermokinetic capacities and resistances can be defined. Then it derives the model equations by using the computer algebra system Mathematica (Wolfram Research, 2010). TKMOD automatically performs reduction steps for fast reactions with *R*_*j*_ = 0 and writes a FORTRAN code for the simulation equations. TKMOD uses DASKR that is a solver for differential-algebraic equation systems for simulation (Brown et al., 1994, 1998, 2007). A version of TKMOD including DASKR is packaged together with the model files and available from https://seek.sysmo-db.org/models/23.

#### 2.1.9. Parameters

The model described above has different types of parameters. The stoichiometric parameters of the reactions are taken mainly from Reed et al. (2003). The amount of translocated protons during electron transport follows Borisov et al. (2011). The thermokinetic capacities are computed from the standard Gibbs energies of formation. Standard Gibbs formation energies for most metabolites are taken from Alberty (2003). Thermodynamic data for the ubiquinone/quinol and menaquinone/quinol pairs are from Alvarez et al. (2013). Data for metabolites in the pentose phosphate pathway are taken from Kümmel et al. (2006b). The parameters of gene expression, gene regulation and the thermokinetic resistances are manually adjusted in order to fit the experimental data. The thermokinetic resistance *R*_*j*_ of reaction *j* is related to the thermokinetic capacities of the reactants *C*_*i*_ and the specific forward rate constant *k*_+*j*_ by *R*_*j*_ = *k*^−1^_+*j*_ · ∏_*i* ∈ *E*_*j*__ *C*^−|ν_*ij*_|^_*i*_. In order to restrict the search space, we assume that *k*_+*j*_ can take only values of the type 10^*x*^ with an integer *x*. Similarly, we assume that the parameters *k*_*i*_ of the gene expression model can take only integer values. The quality of the fit we achieve with this high restriction suggests that the order of magnitude of the resistances determines most of the behavior of the model and that many features of the model are robust against uncertainties in the exact values.

#### 2.1.10. Comparison of simulation and experimental data

Measurement data on biomass concentration, yield factors and fluxes can be compared directly to the simulation results. Valgepea et al. (2010) observe a high correlation between transcript levels and enzyme concentrations for similar chemostat conditions. Also for the gene expression data used in this study a high correlation was observed (Rolfe et al., 2011; Trotter et al., 2011). For this reason, we are able to compare measured transcript levels with the simulated enzyme concentrations. The direct use of measured metabolite concentrations in the mathematical model is subject to several uncertainties. The Gibbs formation energies used to parametrize the model are measured for dilute aqueous solution different from the crowded cytoplasm (Cossins et al., 2011). Systematic losses during quenching and probe preparation may prevent an absolute quantification. For these reasons, we treat the measured metabolite concentrations as relative values.

Relative measurement values *x*_*m*_ (transcripts, metabolites) are scaled with a factor *f* before comparing them (in a plot) with the respective simulated variables *x*_*s*_. The factor *f* is calculated such that the quadratic difference ∥1/f^*^x_*s*_ − *x*_*m*_∥^2^_2_ between measured an simulated variables is minimal and can be computed as $f=\frac{{\vec{x}}_{s}^{T}\cdot {\vec{x}}_{s}}{{\vec{x}}_{m}^{T}\cdot {\vec{x}}_{s}}$. Here, $\vec{x}$_*s*_ and $\vec{x}$_*m*_ denote the vectors with corresponding pairs of simulated and measured data points. This means that *x*_*s*_ is plotted together not with *x*_*m*_ but with *f* · *x*_*m*_.

### 2.2. Experimental data

Experimental conditions and strain *E. coli* MG1655 were as described in (Rolfe et al., 2012). Biomass and extracellular metabolite concentrations were determined as in (Steinsiek et al., 2011) Transcript data were measured via DNA microarray technology and are taken from (Rolfe et al., 2011). Measurements were complemented with RT-PCR data as described in (Steinsiek et al., 2011).

For determination of intracellular metabolite concentrations cells were first quenched following the method of Link et al. (2008) and afterwards a modified extraction procedure published by Ritter et al. (2008) was used. Following the method of Link et al. (2008) 10 ml of cell containing medium from continuous cultivations were immediately quenched in 20 ml methanol-glycerol solution (60/40% v/v) at −60°C thereby holding the temperature below −20°C. Samples were thoroughly mixed and immediately transferred to dry ice and cooled to −50°C. After centrifugation for 30 min at 10,000g and −20°C the cell pellet was washed with methanol-glycerol solution (60%/40% v/v) at −20°C. After a second centrifugation step all the supernatant was removed and the pellet was kept at −80°C until extraction. The cell pellet was extracted with 1 ml methanol and immediately after resuspension 500 μl of trichloromethane were added and the solution was mixed vigorously. The sample was split into three aliquots and 450 μl trichloromethane pre-chilled on ice were added to each aliquot. Samples were thoroughly vortexed. Afterwards 900 μl of methanol/tricine buffer (9:10 parts; final concentration of tricine 1 mM, pH = 7.4) were added, the sample was vortexed again and centrifuged for 10 min at 16,000 g at 4°C. 800 μl of the upper (hydrophilic) phase were collected and stored. This step was repeated and the supernatant was collected, combined with the first sample and boiled for 4 min at 90°C. The sample was again centrifuged at 16000g at 4°C and the supernatant was evaporated to dryness under nitrogen stream. Samples were afterwards analyzed by anion exchange chromatography using a BioLC type DX320 (Dionex) as described by Ritter et al. (2006).

Quinone/ol concentrations were determined as described in (Bekker et al., 2007).

## 3. Results and discussion

We present a mathematical model of the oxygen response of an *E. coli* population in a glucose-limited chemostat. Modeling is facilitated by the restriction to steady state conditions and the use of the aerobiosis scale. The use of the aerobiosis scale allows the integration of the experiments in different reactors with one unique parameter set. Due to the restriction to steady state conditions, differences in initial conditions (e.g., initial pH, cell density, gene expression levels) do not need to be considered. The overall model contains a thermokinetic model of the metabolic network. The model reproduces the effect of reactants, products, important activators and inhibitors, as well as the enzyme concentrations on the metabolic reaction rate by simplified kinetic laws. The metabolic model is complemented by a gene expression model. The synthesis rate of enzymes depends on the activities of transcription factors. The activities of transcription factors depends in turn on their respective metabolic signals. Information about the network structure is based on the EcoCyc database (Keseler et al., 2013). The metabolism and regulation model are embedded into a model describing the growth of the bacterial population and the chemostat environment. The final model is able to provide an integrated description of metabolic fluxes and concentrations, gene expression levels and genetic regulation. A further hallmark of the model is that the balances of the metabolites ATP, ADP, and AMP, as well as NADH, NAD, NADPH, and NADP are explicitly considered. In many models of smaller subnetworks the concentrations of these ubiquitous metabolites are assumed to be constant because only a small subset of all producing and consuming reactions is modeled. The present model seeks a complete description of the balance of these metabolites and thus reflects the constraints that arise from energy and redox requirements.

The parameters of the model fall into two classes: (1) The stoichiometric parameters and the Gibbs formation energies are largely organism-independent and can be taken from available databases. (2) The thermokinetic resistances and the parameters of gene expression and gene regulation are free and (within bounds) not subject to physical constraints. Their values depend on properties of enzymes, transcription factors, and consensus sequences that may vary between strains. By adjusting the different parameter values for the latter class of parameters, the model can describe different physically feasible behaviors of the cell population. In order to test the model, the free parameters are adapted to a data set describing a steady state chemostat at different levels of aerobiosis. The data set includes metabolite concentrations, gene expression data and uptake and excretion fluxes. The model reproduces the steady state values of most measured variables and predicts the values of many others at several values of aerobiosis. This means that the model is able to describe the steady state response of *E. coli* to oxygen.

Despite the complexity and size of the considered system, the use of simplifying assumptions keeps the model tractable. For most reactions a constant thermokinetic resistance is assumed. The resistance is allowed to vary only by integer factors of 10 to restrict search space. Resistances of rapid reactions are assumed to be zero allowing for a reduction of model size and stiffness. Gene expression and gene regulation are described with phenomenological equations. Parameters describing the influence of transcription factors on gene expression are allowed to take only integer values. Growth and maintenance are described by reactions with phenomenological kinetics. The rationale behind these simplifications is that they facilitate modeling and parameter adaptation while still preserving the basic physical and regulatory constraints on the cellular behavior.

The following section presents the comparison of model results and experimental data for several aerobiosis values. The subsequent section demonstrates the use of the model by providing predictions of the effects of production pathways on the central metabolism.

### 3.1. Behavior across the aerobiosis scale

**Figure 1:**
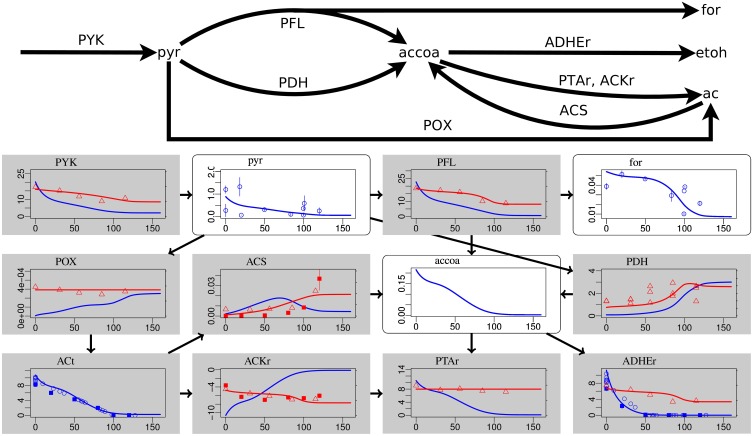
**Steady-state simulation results of the fermentation pathways in comparison to measurement data**. The abscissa is aerobiosis in percent. White boxes represent metabolites. The ordinate is in nmol/g. Blue lines and circles show simulation and measurement data, respectively. The error bars show the technical standard deviation. Gray boxes represent reactions. The blue lines show the reaction flux in mmol/g/h. Blue symbols show flux data computed from the steady state concentration of extracellular concentrations. Different symbols indicate that the data were measured in different laboratories. The red lines show gene expression in arbitrary units. If two red lines are shown, they refer to different genes with qualitatively different expression. Red triangles show microarray data (Rolfe et al., 2011). If for one reaction several triangles are shown, they refer to different genes. Red squares show RT-PCR data with standard deviation. Labels from left to right and top to bottom: PYK pyruvate kinase, pyr pyruvate, PFL pyruvate formate lyase, for formate, POX pyruvate oxidase, ACS acetyl-CoA synthetase, accoa acetyl-CoA, PDH pyruvate dehydrogenase, ACt acetate transport, ACKr acetate kinase, PTAr phosphotransacetylase, ADHEr acetaldehyde dehydrogenase.

**Figure 2:**
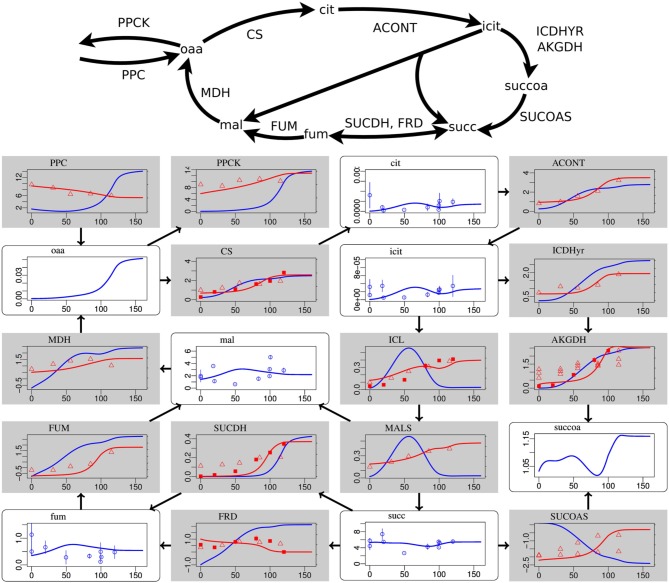
**Steady-state simulation results of the citric acid cycle in comparison to measurement data**. For explanations see caption of Figure 1. Labels from left to right and top to bottom: PPC phosphoenolpyruvate carboxylase, PPCK phosphoenolpyruvate carboxykinase, cit citrate, ACONT aconitase, oaa oxaloacetate, CS citrate synthase, icit Isocitrate, ICDHyr isocitrate dehydrogenase (NADP), MDH malate dehydrogenase, mal L-malate, ICL isocitrate lyase, AKGDH 2-oxogluterate dehydrogenase, FUM fumarase, SUCDH succinate dehydrogenase, MALS malate synthase, succoa succinyl-CoA, fum fumarate, FRD succinate dehydrogenase, succ succinate, SUCOAS succinyl-CoA synthetase.

**Figure 3:**
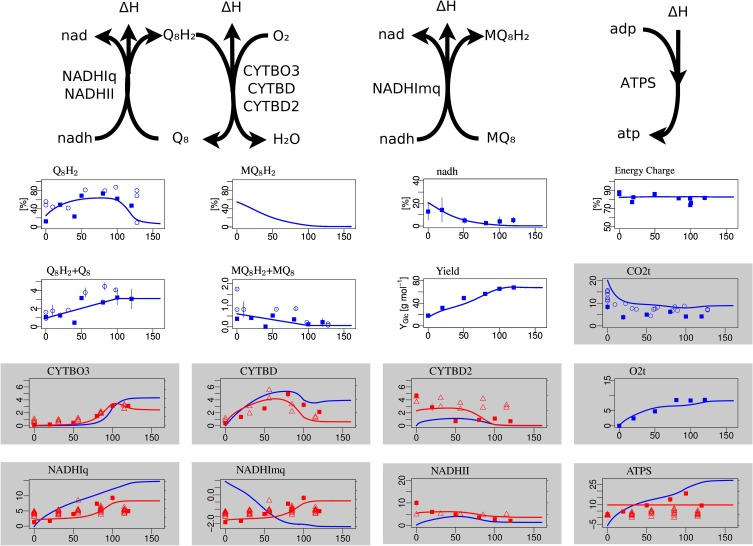
**Steady-state simulation results of the electron transport chain in comparison to measurement data**. The plots with white background in the first row show the degree of reduction of ubiquinones, menaquinones and NADH and the ATP energy charge, respectively. The second row shows the total concentration (oxidized plus reduced form) of ubiquinone and menaquinone and the substrate-biomass yield *Y*. The labels for the gray boxes mean: CO2t CO2 transport through cytoplasmatic membrane, CYTBO3 cytochrome oxidase *bo*, CYTBD cytochrome oxidase *bd*, CYTBD2 cytochrome oxidase *bd2*, O2t o2 transport, NADHIq8 NADH dehydrogenase (ubiquinone-8) *nuo*, NADHImq NADH dehydrogenase (menaquinone-8) *nuo*, NADHII NADH dehydrogenase (ubiquinone-8) *ndh*, ATPS ATP synthase.

**Figure 4:**
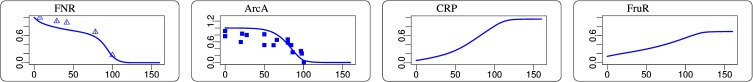
**Steady-state simulation results of some transcription factor activities in comparison to measurement data**. The abscissa is aerobiosis in percent. The ordinate is in arbitrary units. FNR data from Rolfe et al. (2011). ArcA data was determined as described in Rolfe et al. (2012).

The model is compared to measurement data of transcripts, metabolites and uptake/excretion fluxes for several values of aerobiosis. Under identical (Rolfe et al., 2011; Trotter et al., 2011) and similar experimental conditions (Valgepea et al., 2010), it was shown that transcript levels correlate well with protein levels. Thus, here we compare modeled enzyme levels with measured transcript levels. Figures 1–4 show important parts of the model's results. Supplementary Data Sheet 4 shows a more complete overview of the simulation results.

Most measurement data at the flux, metabolite, transcript, and gene regulatory level are reproduced in an integrated, thermodynamically consistent way by our model. The relative tendencies of metabolite concentrations are described well for glycolysis (Supplementary Data Sheet 4) and fermentation pathways (Figure 1). For low oxygen availability the biomass yield is low and glucose uptake is high to allow for a growth rate equal to the dilution rate. Consequently, the metabolite concentrations in the first steps of glycolysis (glucose 6-phosphate g6p, fructose 6-phosphate f6p, and fructose 1,6-bisphosphate fdp) before the enzymatic reaction catalyzed by glyceraldehyde-3-phosphate dehydrogenase GAPD decrease with oxygen availability. GAPD catalyzes a rapid reaction with NADH as a product. Because the concentration of NADH decreases strongly at high aerobiosis values, the thermodynamic pull of NADH reverses the pattern for 3-phospho-glycerophosphate 13dpg, glycerate 2-phosphate 2pg, 3-phospho-glycerate 3pg, and phosphoenolpyruvate pep. The pattern is again inverted after the essentially irreversible pyruvate kinase PYK such that pyruvate pyr and the metabolites of the fermentation pathways are high for low oxygen availability and low for high oxygen availability. This is consistent with the observed inverse correlation of fermentation product excretion and aerobiosis. As a test to check if the model could predict trends of metabolite concentrations, we computed the steady state solutions for different dilution rates that in a steady-state chemostat are equal to the growth rates. The results are qualitatively consistent with the experimental observation from Schaub and Reuss (2008) in that the concentrations of fructose 1,6-bisphosphate, dihydroxyacetone phosphate, and glyceraldehyde 3-phosphate increase with dilution rate, whereas the concentrations of phosphoenolpyruvate, glycerate 2-phosphate and 3-phospho-glycerate decrease with dilution rate.

The concentrations of the different quinone species in the electron transport chains are well matched, in particular the non-monotone behavior of the ubiquinone redox state (Figure 3). The NADH redox state follows the expected pattern from high reduction potential at low arerobiosis values to low reduction potential at high aerobiosis values. The same holds for the menaquinone redox state that however could not be experimentally measured. The level of oxidized ubiquinone q8 correlates to the concentration of its oxidant, oxygen, with low but almost constant levels for microaerobic levels and high level in the fully aerobic state. The strongly differentially regulated *de novo* synthesis of ubiquinones leads to strong increase of the total concentration (oxidized plus reduced) of ubiquinones. In the microaerobic range this leads to a seemingly paradoxical increase of the concentration of reduced ubiquinols such that maximal reduction is reached for intermediate aerobiosis levels and not for anaerobic growth.

Deviations occur for metabolites in the citric acid cycle (Figure 2) and the pentose phosphate pathway (Supplementary Data Sheet 4). Since the dynamic ranges of these metabolites are small in both, measurement and simulation, and since transcript levels and uptake and production fluxes are described well by the model, these deviations are considered to be minor. Driven by the redox state of NADH, the citric acid cycle shows the switch from its branched form at low aerobiosis values to the cyclic form at high aerobiosis values. The branched mode is characterized by reaction fluxes directed from oxaloacetate (oaa) and citrate (cit) to succinate (succ) and 2-oxoglutarate akg, whereas in the cyclic mode the 2-oxogluterate dehydrogenase AKGDH couples both branches and flux occurs in the direction from succinate succ to oxaloacetate (oaa). Succinate dehydrogenase SUCDH and fumarate reductase FRD participate in the cyclic and branched modes, respectively. This selectivity is caused by differential gene expression and the different use of quinone species (menaquinone for FRD and ubiquinone for SUCDH). Thus, the model reflects the expected behavior of the citric acid cycle for different oxygen availabilities. The model comprises the anaplerotic reaction phosphoenolpyruvate carboxylase PPC and the reverse phosphoenolpyruvate carboxykinase PPCK as well as the anaplerotic glyoxylate shunt realized by isocitrate lyase ICL and malate synthase MALS. The model is able to fit the data for different distributions of anaplerosis over both possible pathways (PPC/PPCK and ICL/MALS), and the simulation results present only one possibility.

The activities of the two measured transcription factors in the simulation and measurement are in agreement (Figure 4). FNR and ArcA activities follow the expected pattern of high activity at low aerobiosis values and low activity at high aerobiosis values (Sawers, 1999). The model provides predictions for the behavior of several other transcription factors that could not be measured. An indirect validation of these predictions is provided by the good fits of the transcript data. Some glycolytic enzymes are repressed by FruR and the slight increase of FruR activity across the aerobiosis scale is likely. The increase of CRP can be seen for example in the increased expression of the *mgl* operon that is activated by CRP. The *mgl* operon encodes a methyl-β-D-galactoside and galactose ABC transporter. Since this transporter is also able to transport glucose and is active under glucose-limited conditions (Death and Ferenci, 1993; Ferenci, 1996; Hua et al., 2004; Steinsiek and Bettenbrock, 2012) it is modeled as a glucose transporter (GLCabc). The simulated CRP activity is also consistent with the inferred CRP activity from Rolfe et al. (2012). Since FruR activity is controlled by the concentration of fructose 1,6-bisphosphate (fdp) and CRP activity is controlled by the ratio of phosphoenolpyruvate (pep) to pyruvate (pyr), these predictions follow directly from the distribution of concentrations in glycolysis. The observation that PdhR is more active under aerobic conditions than under anaerobic conditions (Supplementary Data Sheet 4) is consistent with the experimental observation of Ogasawara et al. (2007). AppY is known to be a regulator active under anaerobic conditions (Brøndsted and Atlung, 1996; Atlung et al., 1997) as it is in the model (Supplementary Data Sheet 4). The transcript levels of both target genes of IclR (MALS and ICL in Figure 2) match the predicted course of IclR activity (Supplementary Data Sheet 4).

### 3.2. Assessment of production capabilities

We use the above described model to assess the production capabilities of *E. coli* in a chemostat. For this purpose, we introduce a reaction into the model that represents the effect of a production pathway. For example, if a pathway uses the precursors A and B and 2 ATP to produce a product (possibly via some intermediates), we introduce the reaction A + B + 2·ATP → 2·ADP. For every production pathway, we show steady state values of several quantities depending on the enforced biomass-specific production flux *J*_prod_ given in mmol/g/h. These quantities are the biomass concentration, concentrations of precursors of the production pathway and the overall productivity per reactor volume *q*_prod_ in mmol/l/h. The productivity is given by *q*_prod_ = *J*_prod_ · *c*_X_ where *c*_X_ is the biomass concentration in g/l. In this way, we can assess the production capabilities independently of the kinetics of the production pathway.

**Figure 5:**
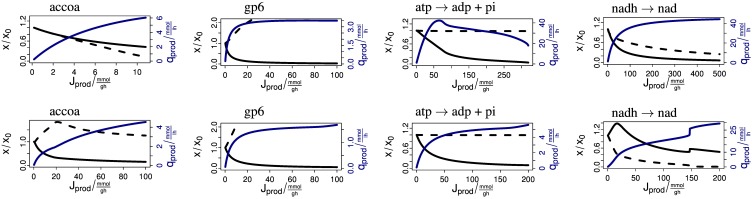
**Steady-state response of the model to enforced precursor consumption rates**. The upper and lower rows show the results for the aerobic and anaerobic case, respectively. The abscissas shows the biomass-specific production flux in mmol/g/h. The black solid lines show the biomass. The dashed lines show the intracellular concentration of the respective metabolite. The curves are scaled to the undisturbed case and start at unity. The blue lines show the productivity in mmol/l/h. Labels mean accoa acetyl-CoA, g6p glucose 6-phosphate, atp → adp + pi dephosphorylation of ATP, nadh → nad oxidation of NADH.

Figure 5 shows results for hypothetical production pathways where each pathway requires only a single precursor molecule. These results demonstrate the abilities and limitations of the central metabolism to provide biosynthesis with precursors. The aerobic and the anaerobic case are shown. For the aerobic case aeration is fixed to a level that results in 160% aerobiosis of the undisturbed case (*J*_prod_ = 0). Plots of all twelve precursor molecules are shown in the Supplementary Data Sheet 5.

The concentration of glucose 6-phosphate (g6p) increases with production flux, i.e., the consumption rate of glucose 6-phosphate by the production pathway, under aerobic and anaerobic conditions (Figure 5). This seemingly paradoxical behavior is explained by the autocatalytic structure of glycolysis. Glucose is phosphorylated to glucose 6-phosphate using ATP (or phosphoenolpyruvate). ATP is regained by metabolism of glucose 6-phosphate. An additional consumption of glucose 6-phosphate leads to an increase of the glucose uptake flux. An increased glucose uptake requires more ATP for phosphorylation of glucose. Since a fixed flux of ATP is required to form biomass in the steady state chemostat, the flux away from glucose 6-phosphate increases and provides a higher ATP production flux. Because the ATP concentration is kept nearly constant and since glycolytic enzymes are nearly constitutively expressed, an increase of the glucose 6-phosphate concentration is needed to drive this flux in order to achieve a steady state. Depending on the kinetics of the production pathway, this behavior may cause dynamic instabilities such as oscillations.

Aerobically, the concentration of acetyl-CoA (accoa) and the biomass concentration drop with an increasing consumption of acetyl-CoA (Figure 5 upper row). Anaerobically, the concentration of acetyl-CoA increases initially and then decreases slightly (Figure 5 lower row). However, the biomass concentration decreases rapidly. In the model, up to 6 mmol/l/h acetyl-CoA can be produced aerobically and up to 5 mmol/l/h anaerobically. Due to the much smaller biomass concentration in the anaerobic case, this corresponds to a much higher biomass-specific flux in the anaerobic case.

Aerobically and anaerobically, the concentration of ATP (atp) decreases only slightly with a consumption of ATP (Figure 5). This is in line with the observation that the energy charge is kept constant over a wide range of conditions (Chapman et al., 1971). In the model this behavior emerges from the activation of the phosphofructokinase by ADP and by the high sensitivity of growth and maintainenance with respect to the ATP/ADP ratio. Aerobically, we obtain the maximal volume-specific ATP production flux for intermediate values of the biomass-specific ATP production flux, whereas for the anaerobic case volume-specific and biomass-specific flux are related monotonously.

Aerobically, the oxidation of NADH (nadh) from the cell leads to a decrease of the (already low) NADH concentration and the biomass (Figure 5 upper row). But anaerobically, the oxidation of NADH has initially a positive effect on the population size (Figure 5 lower row), because it removes the requirement for the excretion of carbon in the form of ethanol to maintain the electron balance.

**Figure 6:**
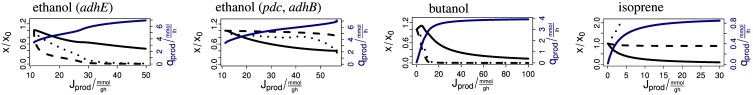
**Steady-state response of the model to enforced biofuel production**. The abscissas shows the biomass-specific production flux in mmol/g/h. The black solid lines show the biomass. The dashed and dotted lines show the intracellular concentration of the relevant redox and carbon precursor, respectively. From left to right: NADH and AcCoA for ethanol (*adhE*), ubiquinol and pyruvate for ethanol (*pdc, adhB*), NADH and AcCoA for butanol, and NADH and pyruvate for isoprene. The curves are scaled to the undisturbed case and start at unity. The blue lines show the productivity in mmol/l/h.

The above examples show that the model provides detailed predictions about the complex effects of production pathways on central metabolism. The synthesis of any biofuel requires a combination of precursor molecules. Figure 6 shows four anaerobic example cases. (1) *E. coli* naturally produces ethanol via the aldehyde-alcohol dehydrogenase AdhE with stoichiometry AcCoA + 2 NADH → CoA + 2 NAD + ethanol. If the flux is increased (for example by overexpression of AdhE), the AcCoA and NADH concentrations drop rapidly. (2) Ohta et al. (1991) expressed the ethanol production pathway from *Zymomonas mobilis* in *E. coli*. The pathway uses the pyruvate decarboxylase Pdc and the alcohol dehydrogenase II AdhB with the stoichiometry pyruvate + ubiquinol → ubiquinone + ethanol. When enforcing an increasing flux via this pathway, the concentrations of the precursors ubiquinol and pyruvate drop initially only slightly. Only immediately before reaching the maximum productivity do the concentrations decrease. This demonstrates the superior properties of this pathway over the native pathway. Figure 6 shows that pyruvate supply becomes limiting. (3) Inui et al. (2008) uses a pathway from *Clostridium acetobutylicum* for the production of butanol in *E. coli*. Its total stoichiometry is 2 AcCoA + 4 NADH → 2 CoA + 4 NAD + butanol. The concentrations of the precursors AcCoA and NADH decrease strongly with the production flux. The biomass concentration initially rises because the removal of reducing power is advantageous under anaerobic conditions. (4) The last example is the production of isoprene via the methylerythritol phosphate (MEP) pathway (Zhao et al., 2011). The overall stoichiometry is glyceraldehyde 3-phosphate + pyruvate + 2 NADPH + NADH + 1 ATP → isoprene + 2 NADP + NAD + AMP + ADP + diphosphate + CO_2_. When enforcing an increasing flux over this pathway, the biomass concentration decreases strongly until it approaches zero. The ATP concentration stays almost constant, the concentrations of NADH and NADPH (not shown) drop slightly and the concentrations of pyruvate and glyceraldehyde 3-phosphate (not shown) increase. Since biomass is clearly the limiting resource in this example, supporting biomass production by providing a complex medium may increase productivity.

The above examples demonstrate the use of the model for assessing the production capabilities of *E. coli*. We enforce the consumption rate of precursor molecules in stoichiometric relations corresponding to the requirements for the synthesis of biofuel. The analysis of the steady state response of the precursor concentrations, the biomass concentration and the volume-specific productivity gives a detailed picture of the capacity of the metabolism in chemostat conditions. In an ideal situation a high production flux is possible and biomass and precursor concentrations are stable over a wide range of production rates. In real examples, either the decrease in biomass concentration or the decrease in concentration of a precursor limits the productivity of the pathway. The model gives detailed predictions about the responses of metabolism to specific production pathways. For example, the model makes predictions for which biomass-specific production flux the highest volume-specific productivity occurs. The model predicts how precursor concentrations change with varying production rate. For pathways with several precursors, the model shows which precursor concentrations decrease strongly and become limiting. In addition, complex phenomena can be observed. For example, it is possible that, due to autocatalytic effects, the concentration of a precursor increases despite an enforced consumption rate of the precursor. Depending on the kinetics of the production pathway, this may lead to dynamic instabilities that could be manifest as oscillations.

These results are predictions of the model. The approach of dissecting the parameters into an experiment- and organism-independent class and a class that is variable within bounds, assures that the resulting model is generic but can be adapted to specific experimental situations. The validity of model predictions decreases when one departs from the experimental conditions that were used to determine the values of the free parameters. Under changed experimental conditions, other, currently unmodeled, regulatory systems may play a relevant role. Changed experimental conditions may be the introduction of production pathways, as discussed here, the use of different substrates, dilution rates or strains, However, since the model by construction cannot violate the basic mass balance and thermodynamic constraints, the predictions will share the features enforced by these constraints.

## 4. Conclusion

To our knowledge, this model presents the first comprehensive, integrated model of steady state metabolism and regulation of the oxygen response of *E. coli*. A model of metabolism describing metabolite concentrations and reaction fluxes is complemented with a model of genetic regulation describing gene expression and transcription factor activity. This model of cellular metabolism is embedded into a model of growth describing the requirement of precursor for biomass formation, a model of maintenance describing the non-growth-associated needs for ATP, and a model of the chemostat describing the concentrations of extracellular compounds and biomass. The model provides a thermodynamically consistent view that integrates experimental data at the metabolite, flux, transcript, and regulator levels.

The combined model reflects the major constraints that restrict the behavior of *E. coli*. The use of the thermokinetic modeling formalism and the availability of the Gibbs formation energies of the metabolites assures that the metabolic model is thermodynamically consistent. This means that the energetic constraints of the cellular metabolism are properly addressed. The mass balances of all relevant metabolites of central metabolism are considered. In particular the model includes also the balances of the biosynthetic precursors and of important energy and redox carriers, as ATP/ADP/AMP, NADH/NAD, and NADPH/NADP. Thus, the model integrates major mass balance constraints with the respective energetic constraints and the respective cellular regulation. The model is able to describe the competition of different pathways for energy and carbon and provides predictions of the effect of the introduction of production pathways on central metabolism.

## Funding

We thank the ERASysbio SysMO (Systems Biology of Microorganisms) initiative for funding the SUMO and SUMO2 consortia. The research was funded by the Biotechnology and Biological Sciences Research Council (BBSRC), the Bundesministerium für Bildung und Forschung (BMBF) and the Nederlandse Organisatie voor Wetenschappelijk Onderzoek (NWO). ME further acknowledges support by the Ministerium für Wissenschaft, Forschung und Kunst Baden-Württemberg within the Ideenwettbewerb Biotechnologie und Medizintechnik Baden-Württemberg.

### Conflict of interest statement

The authors declare that the research was conducted in the absence of any commercial or financial relationships that could be construed as a potential conflict of interest.

## Supplementary material

Supplementary Data Sheet 1 **Index of Model Elements**.

Supplementary Data Sheet 2 **Model Definition File**.

Supplementary Data Sheet 3 **Transcription Factors and Their Metabolic Signals**.

Supplementary Data Sheet 4 **Map of Measurement Data and Model Results**.

Supplementary Data Sheet 5 **Steady State Answer of the Model to enforced precursor effluxes**.
