# Supplementary material for: A mathematical model of metabolism and regulation provides a systems-level view of how Escherichia coli responds to oxygen
Source: Front Microbiol. 2014 Mar 27;5:124. doi: 10.3389/fmicb.2014.00124 (PMC3973912; doi:10.3389/fmicb.2014.00124)
Supplement: Supplementary Data Sheet 1 — Index of Model Elements. [file DataSheet1.PDF]

Index of Model Elements of the Model
Escherichia coli

February 26, 2014

This index provides basic information on the elements of the model `Escherichia coli`. In particular, it provides a reference for the short identifiers of the model elements. The index is ordered alphabetically with respect to the identifiers. It is generated automatically from the model definition file.

+

Type	Compound
Name	elementary charge
Charge	1
Compartment	e

Comment: This is a dummy compound.It is used for chargebalancing in de novosynthesis reactions.This avoids warningmessages. It has noinfluence on the modelresults.

13dpg

Type	Compound
Name	3-Phospho-D-glyceroyl phosphate
Formula	C3H4O10P2
Charge	-4
Compartment	c

2ddg6p

Type	Compound
Name	2-Dehydro-3-deoxy-D-gluconate 6-phosphate
Formula	C6H8O9P
Charge	-3
EcoCyc	`http://biocyc.org/ECOLI/NEW-IMAGE?type=COMPOUND-IN-PATHWAY&object=2-KETO-3-DEOXY-6-P-GLUCONATE`
Compartment	c

2pg

Type	Compound
Name	D-Glycerate 2-phosphate
Formula	C3H4O7P
Charge	-3
Compartment	c

3pg

Type	Compound
Name	3-Phospho-D-glycerate
Formula	C3H4O7P
Charge	-3
Compartment	c

6pgc

Type	Compound
Name	6-Phospho-D-gluconate
Formula	C6H10O10P
Charge	-3
Compartment	c

6pgl

Type	Compound
Name	6-phospho-D-glucono-1,5-lactone
Formula	C6H9O9P
Charge	-2
Compartment	c

ac

Type	Compound
Name	Acetate
Formula	C2H3O2
Charge	-1
Compartment	c

accoa

Type	Compound
Name	Acetyl-CoA
Formula	C23H34N7O17P3S
Charge	-4
Compartment	c

ac(e)

Type	Compound
Name	Acetate (e)
Formula	C2H3O2
Charge	-1
Compartment	e

AC:e->p

Type	Flux
Name	acetate transport through periplasmatic membrane
Reactants	ac(e)
Products	ac(p)
Subsystem	Transport, Extracellular
Compartment	om
Flux from	rapid equilibrium (R=0)

ACKr

Type	Flux
Name	acetate kinase
EC	2.7.2.1
Reactants	ac + atp
Products	actp + adp
Subsystem	Pyruvate metabolism
EcoCyc	http://biocyc.org/ECOLI/NEW-IMAGE?type=REACTION&object=ACETATEKIN-RXN
Compartment	c
Flux from	rapid equilibrium (R=0)

ACONT

Type	Flux
Name	aconitase
EC	4.2.1.3
Reactants	cit
Products	icit
Subsystem	Citrate Cycle (TCA)
EcoCyc	http://biocyc.org/ECOLI/NEW-IMAGE?type=REACTION&object=ACONITATEDEHYDR-RXN
Compartment	c
Flux from	rapid equilibrium (R=0)

ac(p)

Type	Compound
Name	Acetate (p)
Formula	C2H3O2
Charge	-1
Compartment	p

ACS

Type	Flux
Name	acetyl-CoA synthetase
EC	6.2.1.1
Reactants	ac + atp + coa
Products	accoa + amp + ppi
Subsystem	Pyruvate metabolism
EcoCyc	http://biocyc.org/ECOLI/NEW-IMAGE?type=REACTION&object=ACETATE--COA-LIGASE-RXN
Compartment	c
Flux from	thermokinetic law (R>0)

ACt

Type	Flux
Name	acetate transport through cytoplasmatic membrane
Reactants	ac(p) + hP(ACt_h)
Products	ac + h(p+)P(ACt_h)
Subsystem	Transport, Extracellular
Compartment	cm
Flux from	rapid equilibrium (R=0)

actp

Type	Compound
Name	Acetyl phosphate
Formula	C2H3O5P
Charge	-2
Compartment	c

ADHEr

Type	Flux
Name	Acetaldehyde dehydrogenase
EC	1.2.1.10
Reactants	accoa + 2(h + nadh)
Products	coa + etoh + 2nad
Subsystem	Pyruvate metabolism
EcoCyc	http://biocyc.org/ECOLI/NEW-IMAGE?type=REACTION&object=ACETALD-DEHYDROG-RXN
Compartment	c
Flux from	thermokinetic law (R>0)

ADK

Type	Flux
Name	adenylate kinase
EC	2.7.4.3
Reactants	amp + atp
Products	2adp
Subsystem	Nucleotide Salvage Pathways
EcoCyc	http://www.ecocyc.org/ECOLI/NEW-IMAGE?type=REACTION&object=ADENYL-KIN-RXN
Compartment	c
Flux from	rapid equilibrium (R=0)

adp

Type	Compound
Name	ADP
Formula	C10H12N5O10P2
Charge	-3
Compartment	c

akg

Type	Compound
Name	2-Oxoglutarate
Formula	C5H4O5
Charge	-2
Compartment	c

AKGDH

Type	Flux
Name	2-Oxogluterate dehydrogenase
Reactants	akg + coa + nad
Products	co2 + nadh + succoa
Subsystem	Citrate Cycle (TCA)
EcoCyc	`http://biocyc.org/ECOLI/NEW-IMAGE?type=REACTION&object=2OXOGLUTARATEDEH-RXN`
Compartment	c
Flux from	thermokinetic law (R>0)

amp

Type	Compound
Name	AMP
Formula	C10H12N5O7P
Charge	-2
Compartment	c

ampsyn

Type	Flux
Name	de novo synthesis of amp
Reactants	−2+
Products	amp
Compartment	c
Flux from	conventional kinetic law

Comment: de novo synthesis of amp is adjusted such that the total concentration amp+adp+atp is approx. constant

AppY

Type	Compound
Name	Transcription Factor AppY (phosphorylatd form)
Charge	0
EcoCyc	`http://biocyc.org/ECOLI/NEW-IMAGE?type=ENZYME&object=PD00967`
Compartment	c

ArcA

Type	Compound
Name	Transcription Factor ArcA (phosphorylatd form)
Charge	0
EcoCyc	`http://biocyc.org/ECOLI/NEW-IMAGE?type=ENZYME&object=PHOSPHO-ARCA`
Compartment	c

atp

Type	Compound
Name	ATP
Formula	C10H12N5O13P3
Charge	-4
Compartment	c

ATPM

Type	Flux
Name	ATP maintenance requirement
Reactants	atp + h2o
Products	adp + h + pi
Subsystem	Oxidative Phosphorylation
Compartment	c
Flux from	conventional kinetic law

ATPS

Type	Flux
Name	ATP synthase (four protons for one ATP)
EC	3.6.3.14
Reactants	adp + 4h(p+) + pi
Products	atp + 3h + h2o
Subsystem	Oxidative phosphorylation
EcoCyc	http://www.ecocyc.org/ECOLI/NEW-IMAGE?type=REACTION&object=ATPSYN-RXN
Compartment	cm
Flux from	rapid equilibrium (R=0)

c

Type	Compartment
Name	cytoplasm
Temperature	310.15 K
pH	7.6
IonicStrength	0.15 mM

cit

Type	Compound
Name	Citrate
Formula	C6H5O7
Charge	-3
Compartment	c

cm

Type	Compartment
Name	cytoplasmatic membrane
Temperature	310.15 K
pH	7.6
IonicStrength	0.15 mM

co2

Type	Compound
Name	CO2
Formula	CO2
Charge	0
Compartment	c

co2(e)

Type	Compound
Name	CO2 (e)
Formula	CO2
Charge	0
Compartment	e

CO2:e->p

Type	Flux
Name	co2 transport through periplasmatic membrane
Reactants	co2(e)
Products	co2(p)
Subsystem	Transport, Extracellular
Compartment	om
Flux from	thermokinetic law (R>0)

CO2:in

Type	Flux
Name	co2 in
Reactants	0
Products	co2(e)
Subsystem	in and outflow of reactor
Compartment	e
Flux from	conventional kinetic law

co2(p)

Type	Compound
Name	CO2 (p)
Formula	CO2
Charge	0
Compartment	p

CO2t

Type	Flux
Name	CO2 transport through cytoplasmatic membrane
Reactants	co2(p)
Products	co2
Subsystem	Transport, Extracellular
Compartment	cm
Flux from	thermokinetic law (R>0)

coa

Type	Compound
Name	Coenzyme A
Formula	C21H32N7O16P3S
Charge	-4
Compartment	c

coasyn

Type	Flux
Name	de novo synthesis of coa
Reactants	−4+
Products	coa
Compartment	c
Flux from	conventional kinetic law

Comment: de novo synthesis of coa is adjusted such that the total concentration coa+succoa+accoa is approx. constant

CRP

Type	Compound
Name	Transcription Factor CRP-cAMP
Charge	0
EcoCyc	http://biocyc.org/ECOLI/NEW-IMAGE?type=ENZYME&object=CPLX0-226
Compartment	c

CS

Type	Flux
Name	citrate synthase
EC	2.3.3.1
Reactants	accoa + h2o + oaa
Products	cit + coa + h
Subsystem	Citrate Cycle (TCA)
EcoCyc	http://biocyc.org/ECOLI/NEW-IMAGE?type=REACTION&object=CITSYN-RXN
Compartment	c
Flux from	thermokinetic law (R>0)

CYTBD

Type	Flux
Name	cytochrome oxidase bd
EC	1.10.3.-
Reactants	2hP(BD_H) + o2 + 2q8h2
Products	2(h(p+)P(BD_H) + h2o + q8)
Subsystem	Oxidative phosphorylation
EcoCyc	http://www.ecocyc.org/ECOLI/NEW-IMAGE?type=REACTION&object=RXN0-5266
Compartment	cm
Flux from	thermokinetic law (R>0)

CYTBD2

Type	Flux
Name	cytochrome oxidase bd2
EC	1.10.3.-
Reactants	2hP(BD2_H) + o2 + 2q8h2
Products	2(h(p+)P(BD2_H) + h2o + q8)
Subsystem	Oxidative phosphorylation
EcoCyc	http://www.ecocyc.org/ECOLI/NEW-IMAGE?type=REACTION&object=RXN0-5266
Compartment	cm
Flux from	thermokinetic law (R>0)

CYTBO3

Type	Flux
Name	cytochrome oxidase bo
EC	1.10.3.10
Reactants	2hP(BO_H) + o2 + 2q8h2
Products	2(h(p+)P(BO_H) + h2o + q8)
Subsystem	Oxidative phosphorylation
EcoCyc	http://www.ecocyc.org/ECOLI/NEW-IMAGE?type=REACTION&object=RXN0-5268
Compartment	cm
Flux from	thermokinetic law (R>0)

dhap

Type	Compound
Name	Dihydroxyacetone phosphate
Formula	C3H5O6P
Charge	-2
Compartment	c

e

Type	Compartment
Name	cell exterior (medium)
Temperature	310.15 K
pH	7.6
IonicStrength	0.15 mM

e4p

Type	Compound
Name	D-Erythrose 4-phosphate
Formula	C4H7O7P
Charge	-2
Compartment	c

E-ACKr

Type	Compound
Name	enzyme of ACKr
Charge	0
EcoCyc	http://biocyc.org/ECOLI/NEW-IMAGE?type=ENZYME&object=ACETATEKINA-MONOMER
Compartment	c

E-ACKr-syn

Type	Flux
Name	enzyme synthesis
Reactants	0
Products	E-ACKr
Compartment	c
Flux from	conventional kinetic law

E-ACONT

Type	Compound
Name	enzyme of ACONT
Charge	0
EcoCyc	http://biocyc.org/ECOLI/NEW-IMAGE?type=ENZYME&object=CPLX0-7761
Compartment	c

Comment: According to EcoCyc AcnB appears to be the main catabolic enzyme. Thus, we neglect AcnA

E-ACONT-syn

Type	Flux
Name	enzyme synthesis
Reactants	0
Products	E-ACONT
Compartment	c
Flux from	conventional kinetic law

E-ACS

Type	Compound
Name	enzyme of ACS
Charge	0
EcoCyc	http://biocyc.org/ECOLI/NEW-IMAGE?type=GENE&object=EG11448
Compartment	c

E-ACS-syn

Type	Flux
Name	enzyme synthesis
Reactants	0
Products	E-ACS
Compartment	c
Flux from	conventional kinetic law

E-ADHEr

Type	Compound
Name	enzyme of ADHEr
Charge	0
EcoCyc	http://biocyc.org/ECOLI/NEW-IMAGE?type=ENZYME&object=ADHE-CPLX
EcoCyc	http://biocyc.org/ECOLI/NEW-IMAGE?type=ENZYME&object=MHPF-MONOMER
Compartment	c

Comment: MhpF is neglected.

E-ADHEr-syn

Type	Flux
Name	enzyme synthesis
Reactants	0
Products	E-ADHEr
Compartment	c
Flux from	conventional kinetic law

E-ADK

Type	Compound
Name	enzyme of ADK
Charge	0
EcoCyc	http://www.ecocyc.org/ECOLI/NEW-IMAGE?type=ENZYME&object=ADENYL-KIN-MONOMER
Compartment	c

E-ADK-syn

Type	Flux
Name	enzyme synthesis of ADK
Reactants	0
Products	E-ADK
Compartment	c
Flux from	conventional kinetic law

E-AKGDH

Type	Compound
Name	enzyme of AKGDH
Charge	0
EcoCyc	http://biocyc.org/ECOLI/NEW-IMAGE?type=ENZYME&object=2OXOGLUTARATEDEH-CPLX
Compartment	c

E-AKGDH-syn

Type	Flux
Name	enzyme synthesis
Reactants	0
Products	E-AKGDH
Compartment	c
Flux from	conventional kinetic law

E-ATPS

Type	Compound
Name	enzyme of ATPS
Charge	0
EcoCyc	http://www.ecocyc.org/ECOLI/NEW-IMAGE?type=ENZYME&object=ATPSYN-CPLX
Compartment	cm

E-ATPS-syn

Type	Flux
Name	enzyme synthesis of ATPS
Reactants	0
Products	E-ATPS
Compartment	c
Flux from	conventional kinetic law

E-CS

Type	Compound
Name	enzyme of CS
Charge	0
EcoCyc	http://biocyc.org/ECOLI/NEW-IMAGE?type=GENE&object=EG10402
Compartment	c

E-CS-syn

Type	Flux
Name	enzyme synthesis
Reactants	0
Products	E-CS
Compartment	c
Flux from	conventional kinetic law

E-CYTBD

Type	Compound
Name	enzyme of bd
Charge	0
EcoCyc	http://www.ecocyc.org/ECOLI/NEW-IMAGE?type=ENZYME&object=CYT-D-UBIOX-CPLX
Compartment	cm

E-CYTBD2

Type	Compound
Name	enzyme of bd2
Charge	0
EcoCyc	http://www.ecocyc.org/ECOLI/NEW-IMAGE?type=ENZYME&object=APP-UBIOX-CPLX
Compartment	cm

E-CYTBD2-syn

Type	Flux
Name	enzyme synthesis
Reactants	0
Products	E-CYTBD2
Compartment	c
Flux from	conventional kinetic law

E-CYTBD-syn

Type	Flux
Name	enzyme synthesis
Reactants	0
Products	E-CYTBD
Compartment	c
Flux from	conventional kinetic law

E-CYTBO3

Type	Compound
Name	enzyme of bo
Charge	0
EcoCyc	http://www.ecocyc.org/ECOLI/NEW-IMAGE?type=ENZYME&object=CYT-O-UBIOX-CPLX
Compartment	cm

E-CYTBO3-syn

Type	Flux
Name	enzyme synthesis
Reactants	0
Products	E-CYTBO3
Compartment	c
Flux from	conventional kinetic law

EDA

Type	Flux
Name	2-dehydro-3-deoxy-phosphogluconate aldolase
EC	4.1.2.14
Reactants	2ddg6p
Products	g3p + pyr
Subsystem	Pentose Phosphate Cycle
EcoCyc	http://biocyc.org/ECOLI/NEW-IMAGE?type=REACTION&object=KDPGALDOL-RXN
Compartment	c
Flux from	rapid equilibrium (R=0)

EDD

Type	Flux
Name	6-phosphogluconate dehydratase
EC	4.2.1.12
Reactants	6pgc
Products	2ddg6p + h2o
Subsystem	Pentose Phosphate Cycle
EcoCyc	http://biocyc.org/ECOLI/NEW-IMAGE?type=REACTION-IN-PATHWAY&object=PGLUCONDEHYDRAT-RXN
Compartment	c
Flux from	thermokinetic law (R>0)

E-EDA

Type	Compound
Name	enzyme of EDA
Charge	0
EcoCyc	http://biocyc.org/ECOLI/NEW-IMAGE?type=GENE&object=EG10256
Compartment	c

E-EDA-syn

Type	Flux
Name	enzyme synthesis
Reactants	0
Products	E-EDA
Compartment	c
Flux from	conventional kinetic law

E-EDD

Type	Compound
Name	enzyme of EDD
Charge	0
EcoCyc	`http://biocyc.org/ECOLI/NEW-IMAGE?type=ENZYME&object=PGLUCONDEHYDRAT-MONOMER`
Compartment	c

E-EDD-syn

Type	Flux
Name	enzyme synthesis
Reactants	0
Products	E-EDD
Compartment	c
Flux from	conventional kinetic law

E-ENO

Type	Compound
Name	enzyme of ENO
Charge	0
EcoCyc	`http://biocyc.org/ECOLI/NEW-IMAGE?type=ENZYME&object=ENOLASE-CPLX`
Compartment	c

E-ENO-syn

Type	Flux
Name	enzyme synthesis
Reactants	0
Products	E-ENO
Compartment	c
Flux from	conventional kinetic law

E-FBA

Type	Compound
Name	enzyme of FBA
Charge	0
EcoCyc	http://biocyc.org/ECOLI/NEW-IMAGE?type=ENZYME&object=FRUCBISALD-CLASSII
Compartment	c

Comment: According to EcoCyc, FBA Class II has the main activity for glycolysis. Thus, we neglect Class I.

E-FBA-syn

Type	Flux
Name	enzyme synthesis
Reactants	0
Products	E-FBA
Compartment	c
Flux from	conventional kinetic law

E-FDH-H

Type	Compound
Name	enzyme of FDH-H
Charge	0
EcoCyc	http://www.ecocyc.org/ECOLI/NEW-IMAGE?type=ENZYME&object=FHLMULTI-CPLX
Compartment	cm

E-FDH-H-syn

Type	Flux
Name	enzyme synthesis
Reactants	0
Products	E-FDH-H
Compartment	c
Flux from	conventional kinetic law

E-FDH-N

Type	Compound
Name	enzyme of FDH-N
Charge	0
EcoCyc	http://www.ecocyc.org/ECOLI/NEW-IMAGE?type=ENZYME&object=FORMATEDEHYDROGN-CPLX
Compartment	cm

E-FDH-N-syn

Type	Flux
Name	enzyme synthesis
Reactants	0
Products	E-FDH-N
Compartment	c
Flux from	conventional kinetic law

E-FDH-O

Type	Compound
Name	enzyme of FDH-O
Charge	0
EcoCyc	http://www.ecocyc.org/ECOLI/NEW-IMAGE?type=ENZYME&object=FORMATEDEHYDROGO-CPLX
Compartment	cm

E-FDH-O-syn

Type	Flux
Name	enzyme synthesis
Reactants	0
Products	E-FDH-O
Compartment	c
Flux from	conventional kinetic law

E-FRD

Type	Compound
Name	enzyme of FRD
Charge	0
EcoCyc	http://biocyc.org/ECOLI/NEW-IMAGE?type=ENZYME&object=FUMARATE-REDUCTASE
Compartment	c

E-FRD-syn

Type	Flux
Name	enzyme synthesis of FRD
Reactants	0
Products	E-FRD
Compartment	c
Flux from	conventional kinetic law

E-FUM

Type	Compound
Name	enzyme of FUM
Charge	0
EcoCyc	http://biocyc.org/ECOLI/NEW-IMAGE?type=ENZYME&object=FUMARASE-A
Compartment	c

Comment: Because fumA mRNA shows the highest differential expression(compared with fumB and fumC), we assume that the main activity is due to fumA.

E-FUM-syn

Type	Flux
Name	enzyme synthesis of FUM
Reactants	0
Products	E-FUM
Compartment	c
Flux from	conventional kinetic law

E-G6PDH2r

Type	Compound
Name	enzyme of G6PDH2r
Charge	0
EcoCyc	http://biocyc.org/ECOLI/NEW-IMAGE?type=ENZYME&object=GLU6PDEHYDROG-MONOMER
Compartment	c

E-G6PDH2r-syn

Type	Flux
Name	enzyme synthesis
Reactants	0
Products	E-G6PDH2r
Compartment	c
Flux from	conventional kinetic law

E-GAPD

Type	Compound
Name	enzyme of GAPD
Charge	0
EcoCyc	http://biocyc.org/ECOLI/NEW-IMAGE?type=ENZYME&object=GAPDH-A-CPLX
Compartment	c

E-GAPD-syn

Type	Flux
Name	enzyme synthesis
Reactants	0
Products	E-GAPD
Compartment	c
Flux from	conventional kinetic law

E-GLCabc

Type	Compound
Name	enzyme of GLCabc
Formula	R3
Charge	0
EcoCyc	http://biocyc.org/ECOLI/NEW-IMAGE?type=ENZYME&object=ABC-18-CPLX
Compartment	cm

Comment: Because in the microarray data mglAC and mglB are differently expressed andbecause EcoCyc lists an promoter between mglB and mglAC, we distinguish between-these two genes here.

E-GLCabc-mglAC

Type	Compound
Name	enzyme of GLCabc (MglAC only)
Formula	R1
Charge	0
EcoCyc	http://biocyc.org/ECOLI/NEW-IMAGE?type=GENE&object=EG10592
EcoCyc	http://biocyc.org/ECOLI/NEW-IMAGE?type=ENZYME&object=MGLC-MONOMER
Compartment	cm

E-GLCabc-mglAC-syn

Type	Flux
Name	enzyme synthesis
Reactants	0
Products	E-GLCabc-mglAC
Compartment	c
Flux from	conventional kinetic law

E-GLCabc-mglB

Type	Compound
Name	enzyme of GLCabc (MglB only)
Formula	R1
Charge	0
EcoCyc	http://biocyc.org/ECOLI/NEW-IMAGE?type=ENZYME&object=MGLB-MONOMER
Compartment	cm

E-GLCabc-mglB-syn

Type	Flux
Name	enzyme synthesis
Reactants	0
Products	E-GLCabc-mglB
Compartment	c
Flux from	conventional kinetic law

E-GLCabc-syn

Type	Flux
Name	formation of E-GLCabc
Reactants	2E-GLCabc-mglAC + E-GLCabc-mglB
Products	E-GLCabc
Subsystem	Transport, Extracellular
EcoCyc	http://biocyc.org/ECOLI/NEW-IMAGE?type=REACTION&object=ABC-18-RXN
Compartment	cm
Flux from	thermokinetic law (R>0)

E-GLCpts

Type	Compound
Name	enzyme of GLCpts
Charge	0
EcoCyc	http://biocyc.org/ECOLI/NEW-IMAGE?type=ENZYME&object=CPLX-157
Compartment	cm

Comment: We assume that all PTS-Transport glucose occurs via the glucose PTS and none via the mannose PTS.

E-GLCpts-syn

Type	Flux
Name	enzyme synthesis
Reactants	0
Products	E-GLCpts
Compartment	c
Flux from	conventional kinetic law

E-GND

Type	Compound
Name	enzyme of GND
Charge	0
EcoCyc	http://biocyc.org/ECOLI/NEW-IMAGE?type=ENZYME&object=6PGLUCONDEHYDROG-CPLX
Compartment	c

E-GND-syn

Type	Flux
Name	enzyme synthesis
Reactants	0
Products	E-GND
Compartment	c
Flux from	conventional kinetic law

E-HEX1

Type	Compound
Name	enzyme of HEX1
Charge	0
EcoCyc	http://biocyc.org/ECOLI/NEW-IMAGE?type=ENZYME&object=GLUCOKIN-MONOMER
Compartment	c

E-HEX1-syn

Type	Flux
Name	enzyme synthesis
Reactants	0
Products	E-HEX1
Compartment	c
Flux from	conventional kinetic law

E-ICDHyr

Type	Compound
Name	enzyme of ICDHyr
Charge	0
EcoCyc	`http://biocyc.org/ECOLI/NEW-IMAGE?type=ENZYME&object=ISOCITHASE-CPLX`
Compartment	c

E-ICDHyr-syn

Type	Flux
Name	enzyme synthesis
Reactants	0
Products	E-ICDHyr
Compartment	c
Flux from	conventional kinetic law

E-ICL

Type	Compound
Name	enzyme of ICL
Charge	0
EcoCyc	`http://biocyc.org/ECOLI/NEW-IMAGE?type=ENZYME&object=ISOCIT-LYASE`
Compartment	c

E-ICL-syn

Type	Flux
Name	enzyme synthesis of ICL
Reactants	0
Products	E-ICL
Compartment	c
Flux from	conventional kinetic law

E-LDH

Type	Compound
Name	enzyme of LDH
Charge	0
EcoCyc	`http://www.ecocyc.org/ECOLI/NEW-IMAGE?type=ENZYME&object=DLACTDEHYDROGNAD-MONOMER`
Compartment	c

E-LDH-syn

Type	Flux
Name	enzyme synthesis
Reactants	0
Products	E-LDH
Compartment	c
Flux from	conventional kinetic law

E-MALS

Type	Compound
Name	enzyme of MALS
Charge	0
EcoCyc	http://biocyc.org/ECOLI/NEW-IMAGE?type=ENZYME&object=MALATE-SYNTHASE
Compartment	c

Comment: We assume that aceB carries the main activity under our conditions and glcBis mainly responsible for growth on glycolate (see EcoCyc)

E-MALS-syn

Type	Flux
Name	enzyme synthesis of MALS
Reactants	0
Products	E-MALS
Compartment	c
Flux from	conventional kinetic law

E-MDH

Type	Compound
Name	enzyme of MDH
Charge	0
EcoCyc	http://biocyc.org/ECOLI/NEW-IMAGE?type=ENZYME&object=MALATE-DEHASE
Compartment	c

E-MDH-syn

Type	Flux
Name	enzyme synthesis of MDH
Reactants	0
Products	E-MDH
Compartment	c
Flux from	conventional kinetic law

E-MQO

Type	Compound
Name	enzyme of MQO
Charge	0
EcoCyc	http://www.ecocyc.org/ECOLI/NEW-IMAGE?type=ENZYME&object=EG12069-MONOMER
Compartment	c

E-MQO-syn

Type	Flux
Name	enzyme synthesis of MQO
Reactants	0
Products	E-MQO
Compartment	c
Flux from	conventional kinetic law

E-NADHI

Type	Compound
Name	enzyme of NADH-DH I
Charge	0
EcoCyc	http://www.ecocyc.org/ECOLI/NEW-IMAGE?type=ENZYME&object=NADH-DHI-CPLX
Compartment	cm

E-NADHII

Type	Compound
Name	enzyme of NADH-DH II
Charge	0
EcoCyc	http://www.ecocyc.org/ECOLI/NEW-IMAGE?type=GENE&object=EG10649
Compartment	cm

E-NADHII-syn

Type	Flux
Name	enzyme synthesis
Reactants	0
Products	E-NADHII
Compartment	c
Flux from	conventional kinetic law

E-NADHI-syn

Type	Flux
Name	enzyme synthesis
Reactants	0
Products	E-NADHI
Compartment	c
Flux from	conventional kinetic law

ENO

Type	Flux
Name	enolase
EC	4.2.1.11
Reactants	2pg
Products	h2o + pep
Subsystem	Glycolysis/Gluconeogenesis
EcoCyc	`http://biocyc.org/ECOLI/NEW-IMAGE?type=REACTION&object=2PGADEHYDRAT-RXN`
Compartment	c
Flux from	rapid equilibrium (R=0)

E-PDH

Type	Compound
Name	enzyme of PDH
Charge	0
EcoCyc	`http://biocyc.org/ECOLI/NEW-IMAGE?type=ENZYME&object=PYRUVATEDEH-CPLX`
Compartment	c

E-PDH-syn

Type	Flux
Name	enzyme synthesis
Reactants	0
Products	E-PDH
Compartment	c
Flux from	conventional kinetic law

E-PFK

Type	Compound
Name	enzyme of PFK
Charge	0
EcoCyc	`http://biocyc.org/ECOLI/NEW-IMAGE?type=ENZYME&object=6PFK-1-CPX`
Compartment	c

Comment: According to EcoCyc, PFK1 has the main activity. Thus, we neglect PFK2

E-PFK-syn

Type	Flux
Name	enzyme synthesis
Reactants	0
Products	E-PFK
Compartment	c
Flux from	conventional kinetic law

E-PFL

Type	Compound
Name	enzyme of PFL
Charge	0
EcoCyc	http://biocyc.org/ECOLI/NEW-IMAGE?type=ENZYME&object=PYRUVFORMLY-CPLX
Compartment	c

Comment: TdcE is neglected.

E-PFL-syn

Type	Flux
Name	enzyme synthesis of PFL
Reactants	0
Products	E-PFL
Compartment	c
Flux from	conventional kinetic law

E-PGI

Type	Compound
Name	enzyme of PGI
Charge	0
EcoCyc	http://biocyc.org/ECOLI/NEW-IMAGE?type=GENE&object=EG10702
Compartment	c

E-PGI-syn

Type	Flux
Name	enzyme synthesis
Reactants	0
Products	E-PGI
Compartment	c
Flux from	conventional kinetic law

E-PGK

Type	Compound
Name	enzyme of PGK
Charge	0
EcoCyc	http://biocyc.org/ECOLI/NEW-IMAGE?type=ENZYME&object=PGK
Compartment	c

E-PGK-syn

Type	Flux
Name	enzyme synthesis
Reactants	0
Products	E-PGK
Compartment	c
Flux from	conventional kinetic law

E-PGL

Type	Compound
Name	enzyme of PGL
Charge	0
EcoCyc	http://biocyc.org/ECOLI/NEW-IMAGE?type=ENZYME&object=6PGLUCONOLACT-MONOMER
Compartment	c

E-PGL-syn

Type	Flux
Name	enzyme synthesis
Reactants	0
Products	E-PGL
Compartment	c
Flux from	conventional kinetic law

E-PGM

Type	Compound
Name	enzyme of PGM
Charge	0
EcoCyc	http://biocyc.org/ECOLI/NEW-IMAGE?type=ENZYME&object=PHOSGLYCMUTASE
EcoCyc	http://biocyc.org/ECOLI/NEW-IMAGE?type=ENZYME&object=PGMI-MONOMER
Compartment	c

E-PGM-syn

Type	Flux
Name	enzyme synthesis
Reactants	0
Products	E-PGM
Compartment	c
Flux from	conventional kinetic law

E-POX

Type	Compound
Name	enzyme of POX
Charge	0
EcoCyc	http://biocyc.org/ECOLI/NEW-IMAGE?type=ENZYME&object=PYRUVOXID-CPLX
Compartment	c

E-POX-syn

Type	Flux
Name	enzyme synthesis
Reactants	0
Products	E-POX
Compartment	c
Flux from	conventional kinetic law

E-PPA

Type	Compound
Name	enzyme of PPA
Charge	0
EcoCyc	http://www.ecocyc.org/ECOLI/NEW-IMAGE?type=ENZYME&object=CPLX0-243
Compartment	c

E-PPA-syn

Type	Flux
Name	enzyme synthesis of PPA
Reactants	0
Products	E-PPA
Compartment	c
Flux from	conventional kinetic law

E-PPC

Type	Compound
Name	enzyme of PPC
Charge	0
EcoCyc	http://biocyc.org/ECOLI/NEW-IMAGE?type=ENZYME&object=PEPCARBOX-CPLX
Compartment	c

E-PPCK

Type	Compound
Name	enzyme of PPCK
Charge	0
EcoCyc	http://biocyc.org/ECOLI/NEW-IMAGE?type=GENE&object=EG10688
Compartment	c

E-PPCK-syn

Type	Flux
Name	enzyme synthesis
Reactants	0
Products	E-PPCK
Compartment	c
Flux from	conventional kinetic law

E-PPC-syn

Type	Flux
Name	enzyme synthesis
Reactants	0
Products	E-PPC
Compartment	c
Flux from	conventional kinetic law

E-PPS

Type	Compound
Name	enzyme of PPS
Charge	0
EcoCyc	http://biocyc.org/ECOLI/NEW-IMAGE?type=ENZYME&object=PEPSYNTH-CPLX
Compartment	c

E-PPS-syn

Type	Flux
Name	enzyme synthesis
Reactants	0
Products	E-PPS
Compartment	c
Flux from	conventional kinetic law

E-PTAr

Type	Compound
Name	enzyme of PTAr
Charge	0
EcoCyc	http://biocyc.org/ECOLI/NEW-IMAGE?type=ENZYME&object=PHOSACETYLTRANS-CPLX
Compartment	c

E-PTAr-syn

Type	Flux
Name	enzyme synthesis
Reactants	0
Products	E-PTAr
Compartment	c
Flux from	conventional kinetic law

E-PYK

Type	Compound
Name	enzyme of PYK
Charge	0
EcoCyc	http://biocyc.org/ECOLI/NEW-IMAGE?type=ENZYME&object=PKI-COMPLEX
Compartment	c

Comment: According to Ponce et al. (http://www.ncbi.nlm.nih.gov/pubmed/7559366) PykF (PYKI)has a much higher activity than PYKII. Thus we neglect PYKII

E-PYK-syn

Type	Flux
Name	enzyme synthesis
Reactants	0
Products	E-PYK
Compartment	c
Flux from	conventional kinetic law

E-RPE

Type	Compound
Name	enzyme of RPE
Charge	0
EcoCyc	http://biocyc.org/ECOLI/NEW-IMAGE?type=ENZYME&object=RIBULP3EPIM-MONOMER
Compartment	c

E-RPE-syn

Type	Flux
Name	enzyme synthesis
Reactants	0
Products	E-RPE
Compartment	c
Flux from	conventional kinetic law

E-RPI

Type	Compound
Name	enzyme of RPI
Charge	0
EcoCyc	http://biocyc.org/ECOLI/NEW-IMAGE?type=ENZYME&object=RIB5PISOMA-CPLX
EcoCyc	http://biocyc.org/ECOLI/NEW-IMAGE?type=ENZYME&object=RIB5PISOMB-CPLX
Compartment	c

E-RPI-syn

Type	Flux
Name	enzyme synthesis
Reactants	0
Products	E-RPI
Compartment	c
Flux from	conventional kinetic law

E-SUCCt

Type	Compound
Name	enzyme of SUCCt
Charge	0
Compartment	cm

E-SUCCt-syn

Type	Flux
Name	enzyme synthesis
Reactants	0
Products	E-SUCCt
Compartment	c
Flux from	conventional kinetic law

E-SUCDH

Type	Compound
Name	enzyme of SUCDH
Charge	0
EcoCyc	`http://biocyc.org/ECOLI/NEW-IMAGE?type=ENZYME&object=SUCC-DEHASE`
Compartment	cm

E-SUCDH-syn

Type	Flux
Name	enzyme synthesis of SUCDH
Reactants	0
Products	E-SUCDH
Compartment	c
Flux from	conventional kinetic law

E-SUCOAS

Type	Compound
Name	enzyme of SUCOAS
Charge	0
EcoCyc	`http://biocyc.org/ECOLI/NEW-IMAGE?type=ENZYME&object=SUCCCOASYN`
Compartment	c

E-SUCOAS-syn

Type	Flux
Name	enzyme synthesis of SUCOAS
Reactants	0
Products	E-SUCOAS
Compartment	c
Flux from	conventional kinetic law

E-TALA

Type	Compound
Name	enzyme of TALA
Charge	0
EcoCyc	http://biocyc.org/ECOLI/NEW-IMAGE?type=ENZYME&object=TRANSALDOLA-MONOMER
EcoCyc	http://biocyc.org/ECOLI/NEW-IMAGE?type=ENZYME&object=TRANSALDOLB-CPLX
Compartment	c

E-TALA-syn

Type	Flux
Name	enzyme synthesis
Reactants	0
Products	E-TALA
Compartment	c
Flux from	conventional kinetic law

E-THD-PntAB

Type	Compound
Name	enzyme of THD-PntAB
Charge	0
EcoCyc	http://www.ecocyc.org/ECOLI/NEW-IMAGE?type=ENZYME&object=PYRNUTRANSHYDROGEN-CPLX
Compartment	cm

E-THD-PntAB-syn

Type	Flux
Name	enzyme synthesis of THD-PntAB
Reactants	0
Products	E-THD-PntAB
Compartment	c
Flux from	conventional kinetic law

E-THD-SthA

Type	Compound
Name	enzyme of THD-SthA
Charge	0
EcoCyc	http://www.ecocyc.org/ECOLI/NEW-IMAGE?type=ENZYME&object=UDHA-CPLX
Compartment	c

E-THD-SthA-syn

Type	Flux
Name	enzyme synthesis of THD-SthA
Reactants	0
Products	E-THD-SthA
Compartment	c
Flux from	conventional kinetic law

E-TKT1

Type	Compound
Name	enzyme of TKT1
Charge	0
EcoCyc	http://biocyc.org/ECOLI/NEW-IMAGE?type=ENZYME&object=TRANSKETOI-CPLX
EcoCyc	http://biocyc.org/ECOLI/NEW-IMAGE?type=ENZYME&object=CPLX0-1261
Compartment	c

E-TKT1-syn

Type	Flux
Name	enzyme synthesis
Reactants	0
Products	E-TKT1
Compartment	c
Flux from	conventional kinetic law

E-TKT2

Type	Compound
Name	enzyme of TKT2
Charge	0
EcoCyc	http://biocyc.org/ECOLI/NEW-IMAGE?type=ENZYME&object=TRANSKETOI-CPLX
EcoCyc	http://biocyc.org/ECOLI/NEW-IMAGE?type=ENZYME&object=CPLX0-1261
Compartment	c

E-TKT2-syn

Type	Flux
Name	enzyme synthesis
Reactants	0
Products	E-TKT2
Compartment	c
Flux from	conventional kinetic law

etoh

Type	Compound
Name	Ethanol
Formula	C2H6O
Charge	0
Compartment	c

etoh(e)

Type	Compound
Name	Ethanol
Formula	C2H6O
Charge	0
Compartment	e

ETOH:e->p

Type	Flux
Name	ethanol transport through periplasmatic membrane
Reactants	etoh(e)
Products	etoh(p)
Subsystem	Transport, Extracellular
Compartment	om
Flux from	thermokinetic law (R>0)

etoh(p)

Type	Compound
Name	Ethanol
Formula	C2H6O
Charge	0
Compartment	p

ETOHt

Type	Flux
Name	ethanol transport through cytoplasmatic membrane
Reactants	etoh(p) + hP(ETOHt_h)
Products	etoh + h(p+)P(ETOHt_h)
Subsystem	Transport, Extracellular
Compartment	cm
Flux from	rapid equilibrium (R=0)

E-TPI

Type	Compound
Name	enzyme of TPI
Charge	0
EcoCyc	http://biocyc.org/ECOLI/NEW-IMAGE?type=ENZYME&object=TPI
Compartment	c

E-TPI-syn

Type	Flux
Name	enzyme synthesis
Reactants	0
Products	E-TPI
Compartment	c
Flux from	conventional kinetic law

f6p

Type	Compound
Name	D-Fructose 6-phosphate
Formula	C6H11O9P
Charge	-2
Compartment	c

FBA

Type	Flux
Name	fructose-bisphosphate aldolase
EC	4.1.2.13
Reactants	fdp
Products	dhap + g3p
Subsystem	Glycolysis/Gluconeogenesis
EcoCyc	http://biocyc.org/ECOLI/NEW-IMAGE?type=REACTION&object=F16ALDOLASE-RXN
Compartment	c
Flux from	rapid equilibrium (R=0)

FDH-H

Type	Flux
Name	Formate Dehydrogenase H
Reactants	for + h
Products	co2 + h2
Subsystem	Oxidative Phosphorylation
EcoCyc	http://www.ecocyc.org/ECOLI/NEW-IMAGE?type=REACTION&object=FHLMULTI-RXN
Compartment	cm
Flux from	thermokinetic law (R>0)

FDH-N

Type	Flux
Name	Formate Dehydrogenase N
EC	1.1.5.6
Reactants	for + 3h + mqn8
Products	co2 + 2h(e) + mql8
Subsystem	Oxidative Phosphorylation
EcoCyc	http://www.ecocyc.org/ECOLI/NEW-IMAGE?type=REACTION&object=FORMATEDEHYDROG-RXN
Compartment	cm
Flux from	thermokinetic law (R>0)

FDH-O

Type	Flux
Name	Formate Dehydrogenase
EC	1.1.5.6
Reactants	for + 3h + mqn8
Products	co2 + 2h(e) + mql8
Subsystem	Oxidative Phosphorylation
EcoCyc	http://www.ecocyc.org/ECOLI/NEW-IMAGE?type=REACTION&object=FORMATEDEHYDROG-RXN
Compartment	cm
Flux from	thermokinetic law (R>0)

fdp

Type	Compound
Name	D-Fructose 1,6-bisphosphate
Formula	C6H10O12P2
Charge	-4
Compartment	c

FNR

Type	Compound
Name	Transcription Factor FNR (reduced form)
Charge	0
EcoCyc	http://biocyc.org/ECOLI/NEW-IMAGE?type=ENZYME&object=CPLX0-7797
Compartment	c

for

Type	Compound
Name	Formate
Formula	CH1O2
Charge	-1
Compartment	c

for(e)

Type	Compound
Name	Formate (e)
Formula	CH1O2
Charge	-1
Compartment	e

FOR:e->p

Type	Flux
Name	formate transport through periplasmatic membrane
Reactants	for(e)
Products	for(p)
Subsystem	Transport, Extracellular
Compartment	om
Flux from	rapid equilibrium (R=0)

for(p)

Type	Compound
Name	Formate (p)
Formula	CH1O2
Charge	-1
Compartment	p

FORt

Type	Flux
Name	formate transport through cytoplasmatic membrane
Reactants	for(p) + hP(FORt_h)
Products	for + h(p+)P(FORt_h)
Subsystem	Transport, Extracellular
Compartment	cm
Flux from	rapid equilibrium (R=0)

FRD

Type	Flux
Name	succinate dehydrogenase
EC	1.3.5.4
Reactants	mqn8 + succ
Products	fum + mql8
Subsystem	Citrate Cycle (TCA)
EcoCyc	http://biocyc.org/ECOLI/NEW-IMAGE?type=REACTION&object=R601-RXN
Compartment	cm
Flux from	thermokinetic law (R>0)

FruR

Type	Compound
Name	Transcription Factor FruR
Charge	0
EcoCyc	http://biocyc.org/ECOLI/NEW-IMAGE?type=ENZYME&object=CPLX-128
Compartment	c

fum

Type	Compound
Name	Fumarate
Formula	C4H2O4
Charge	-2
Compartment	c

FUM

Type	Flux
Name	fumarase
EC	4.2.1.2
Reactants	fum + h2o
Products	mal
Subsystem	Citrate Cycle (TCA)
EcoCyc	http://biocyc.org/ECOLI/NEW-IMAGE?type=REACTION&object=FUMHYDR-RXN
Compartment	c
Flux from	rapid equilibrium (R=0)

g3p

Type	Compound
Name	Glyceraldehyde 3-phosphate
Formula	C3H5O6P
Charge	-2
Compartment	c

g6p

Type	Compound
Name	D-Glucose 6-phosphate
Formula	C6H11O9P
Charge	-2
Compartment	c

G6PDH2r

Type	Flux
Name	glucose 6-phosphate dehydrogenase
EC	1.1.1.49
Reactants	g6p + nadp
Products	6pgl + h + nadph
Subsystem	Pentose Phosphate Cycle
EcoCyc	http://biocyc.org/ECOLI/NEW-IMAGE?type=REACTION&object=GLU6PDEHYDROG-RXN
Compartment	c
Flux from	thermokinetic law (R>0)

GAPD

Type	Flux
Name	glyceraldehyde-3-phosphate dehydrogenase
EC	1.2.1.12
Reactants	g3p + nad + pi
Products	13dpg + h + nadh
Subsystem	Glycolysis/Gluconeogenesis
EcoCyc	http://biocyc.org/ECOLI/NEW-IMAGE?type=REACTION&object=GAPOXNPHOSPHN-RXN
Compartment	c
Flux from	rapid equilibrium (R=0)

GLCabc

Type	Flux
Name	glucose transport via ABC (mgl)
EC	3.6.3.17
Reactants	atp + glc-D(p) + h2o
Products	adp + glc-D + h + pi
Subsystem	Transport, Extracellular
EcoCyc	http://biocyc.org/ECOLI/NEW-IMAGE?type=REACTION&object=ABC-18-RXN
PMID	8310178
PMID	8703508
PMID	15066832
PMID	22923596
Compartment	cm
Flux from	thermokinetic law (R>0)

glc-D

Type	Compound
Name	D-Glucose (c)
Formula	C6H12O6
Charge	0
Compartment	c

glc-D(e)

Type	Compound
Name	D-Glucose (e)
Formula	C6H12O6
Charge	0
Compartment	e

glc-D(p)

Type	Compound
Name	D-Glucose (p)
Formula	C6H12O6
Charge	0
Compartment	p

GLC:e->p

Type	Flux
Name	glucose transport through periplasmatic membrane
Reactants	glc-D(e)
Products	glc-D(p)
Subsystem	Transport, Extracellular
Compartment	om
Flux from	thermokinetic law (R>0)

GLC:in

Type	Flux
Name	glucose in/out
Reactants	0
Products	glc-D(e)
Subsystem	in- and outflow of glucose into/out of the reactor
Compartment	e
Flux from	conventional kinetic law

Comment: This flux is given by the chemostat equation

GLCpts

Type	Flux
Name	glucose transport via PEP:Pyr PTS
Reactants	glc-D(p) + pep
Products	g6p + pyr
Subsystem	Transport, Extracellular
EcoCyc	`http://biocyc.org/ECOLI/NEW-IMAGE?type=NIL&object=TRANS-RXN-157`
Compartment	cm
Flux from	thermokinetic law (R>0)

glx

Type	Compound
Name	glyoxylate
Formula	C2H1O3
Charge	-1
Compartment	c

GND

Type	Flux
Name	phosphogluconate dehydrogenase
EC	1.1.1.44
Reactants	6pgc + nadp
Products	co2 + nadph + ru5p-D
Subsystem	Pentose Phosphate Cycle
EcoCyc	http://biocyc.org/ECOLI/NEW-IMAGE?type=REACTION&object=6PGLUCONDEHYDROG-RXN
Compartment	c
Flux from	thermokinetic law (R>0)

h

Type	Compound
Name	H+
Formula	H
Charge	1
Compartment	c

h2

Type	Compound
Name	Hydrogen
Formula	H2
Charge	0
Compartment	c

h2(e)

Type	Compound
Name	Hydrogen(e)
Formula	H2
Charge	0
Compartment	e

H2:e->p

Type	Flux
Name	h2 transport through periplasmatic membrane
Reactants	h2(e)
Products	h2(p)
Subsystem	Transport, Extracellular
Compartment	om
Flux from	thermokinetic law (R>0)

H2:in

Type	Flux
Name	h2 in
Reactants	0
Products	h2(e)
Subsystem	in and outflow of reactor
Compartment	e
Flux from	conventional kinetic law

h2o

Type	Compound
Name	H2O
Formula	H2O
Charge	0
Compartment	c

h2o(e)

Type	Compound
Name	H2O (e)
Formula	H2O
Charge	0
Compartment	e

h2o(p)

Type	Compound
Name	H2O (p)
Formula	H2O
Charge	0
Compartment	p

h2(p)

Type	Compound
Name	Hydrogen(p)
Formula	H2
Charge	0
Compartment	p

H2t

Type	Flux
Name	H2 transport through cytoplasmatic membrane
Reactants	h2(p)
Products	h2
Subsystem	Transport, Extracellular
Compartment	cm
Flux from	thermokinetic law (R>0)

h(e)

Type	Compound
Name	H+ (e)
Formula	H
Charge	1
Compartment	e

HEX1

Type	Flux
Name	hexokinase (D-glucose:ATP)
EC	2.7.1.1
Reactants	atp + glc-D
Products	adp + g6p + h
Subsystem	Glycolysis/Gluconeogenesis
EcoCyc	http://biocyc.org/ECOLI/NEW-IMAGE?type=REACTION&object=GLUCOKIN-RXN
Compartment	c
Flux from	rapid equilibrium (R=0)

h(p)

Type	Compound
Name	H+ (p)
Formula	H
Charge	1
Compartment	p

h(p+)

Type	Compound
Name	H+ (p+)
Formula	H
Charge	1
Compartment	p+

ICDHyr

Type	Flux
Name	isocitrate dehydrogenase (NADP)
EC	1.1.1.42
Reactants	icit + nadp
Products	akg + co2 + nadph
Subsystem	Citrate Cycle (TCA)
EcoCyc	http://biocyc.org/ECOLI/NEW-IMAGE?type=REACTION&object=ISOCITDEH-RXN
Compartment	c
Flux from	rapid equilibrium (R=0)

icit

Type	Compound
Name	Isocitrate
Formula	C6H5O7
Charge	-3
Compartment	c

ICL

Type	Flux
Name	isocitrate lyase
EC	4.1.3.1
Reactants	icit
Products	glx + succ
Subsystem	Anaplerotic Reactions
EcoCyc	http://biocyc.org/ECOLI/NEW-IMAGE?type=REACTION&object=ISOCIT-CLEAV-RXN
Compartment	c
Flux from	rapid equilibrium (R=0)

IclR

Type	Compound
Name	Transcription Factor IcR (active form)
Charge	0
EcoCyc	http://biocyc.org/ECOLI/NEW-IMAGE?type=ENZYME&object=PD04099
Compartment	c

kprod

Type	Compound
Charge	0
Compartment	c

lac

Type	Compound
Name	D-Lactate
Formula	C3H5O3
Charge	-1
Compartment	c

lac(e)

Type	Compound
Name	D-Lactate (e)
Formula	C3H5O3
Charge	-1
Compartment	e

LAC:e->p

Type	Flux
Name	lactate transport through periplasmatic membrane
Reactants	lac(e)
Products	lac(p)
Subsystem	Transport, Extracellular
Compartment	om
Flux from	rapid equilibrium (R=0)

lac(p)

Type	Compound
Name	D-Lactate (p)
Formula	C3H5O3
Charge	-1
Compartment	p

LACt

Type	Flux
Name	D-lactate transport through cytoplasmatic membrane
Reactants	hP(LACt_h) + lac
Products	h(p+)P(LACt_h) + lac(p)
Subsystem	Transport, Extracellular
Compartment	cm
Flux from	rapid equilibrium (R=0)

LDH

Type	Flux
Name	D-lactate dehydrogenase
EC	1.1.1.28
Reactants	lac + nad
Products	h + nadh + pyr
Subsystem	Pyruvate metabolism
EcoCyc	http://www.ecocyc.org/ECOLI/NEW-IMAGE?type=REACTION&object=DLACTDEHYDROGNAD-RXN
Compartment	c
Flux from	thermokinetic law (R>0)

mal

Type	Compound
Name	L-Malate
Formula	C4H4O5
Charge	-2
Compartment	c

MALS

Type	Flux
Name	malate synthase
EC	2.3.3.9
Reactants	accoa + glx + h2o
Products	coa + h + mal
EcoCyc	http://biocyc.org/ECOLI/NEW-IMAGE?type=REACTION&object=MALSYN-RXN
Compartment	c
Flux from	thermokinetic law (R>0)

MDH

Type	Flux
Name	malate dehydrogenase
EC	1.1.1.37
Reactants	mal + nad
Products	h + nadh + oaa
Subsystem	Citrate Cycle (TCA)
EcoCyc	http://biocyc.org/ECOLI/NEW-IMAGE?type=REACTION&object=MALATE-DEH-RXN
Compartment	c
Flux from	rapid equilibrium (R=0)

mql8

Type	Compound
Name	Menaquinol 8
Formula	C51H74O2
Charge	0
Compartment	cm

mqn8

Type	Compound
Name	Menaquinone 8
Formula	C51H72O2
Charge	0
Compartment	cm

mqn8syn

Type	Flux
Name	de novo synthesis of mqn8
Reactants	0
Products	mqn8
Compartment	cm
Flux from	conventional kinetic law

Comment: de novo synthesis of mqn8 decreases linearily with aerobiosis

MQO(mqn8)

Type	Flux
Name	malate dehydrogenase
EC	1.1.5.4
Reactants	mal + mqn8
Products	mql8 + oaa
Subsystem	Citrate Cycle (TCA)
EcoCyc	http://www.ecocyc.org/ECOLI/NEW-IMAGE?type=REACTION&object=MALATE-DEHYDROGENASE-ACCEPTOR-RXN
Compartment	c
Flux from	thermokinetic law (R>0)

MQO(q8)

Type	Flux
Name	malate dehydrogenase
EC	1.1.5.4
Reactants	mal + q8
Products	oaa + q8h2
Subsystem	Citrate Cycle (TCA)
EcoCyc	http://www.ecocyc.org/ECOLI/NEW-IMAGE?type=REACTION&object=MALATE-DEHYDROGENASE-ACCEPTOR-RXN
Compartment	c
Flux from	thermokinetic law (R>0)

nad

Type	Compound
Name	Nicotinamide adenine dinucleotide
Formula	C21H26N7O14P2
Charge	-1
Compartment	c

nadh

Type	Compound
Name	Nicotinamide adenine dinucleotide - reduced
Formula	C21H27N7O14P2
Charge	-2
Compartment	c

NADHII(q8)

Type	Flux
Name	NADH dehydrogenase (ubiquinone-8) ndh
EC	1.6.5.9
Reactants	h + nadh + q8
Products	nad + q8h2
Subsystem	Oxidative phosphorylation
EcoCyc	http://www.ecocyc.org/ECOLI/NEW-IMAGE?type=REACTION&object=RXN0-5330
Compartment	cm
Flux from	thermokinetic law (R>0)

NADHI(mqn8)

Type	Flux
Name	NADH dehydrogenase (menaquinone-8) nuo
EC	1.6.5.-
Reactants	5h + mqn8 + nadh
Products	4h(p+) + mql8 + nad
Subsystem	Oxidative phosphorylation
EcoCyc	http://www.ecocyc.org/ECOLI/NEW-IMAGE?type=REACTION&object=RXN0-5388
Compartment	cm
Flux from	thermokinetic law (R>0)

NADHI(q8)

Type	Flux
Name	NADH dehydrogenase (ubiquinone-8) nuo
EC	1.6.5.3
Reactants	5h + nadh + q8
Products	4h(p+) + nad + q8h2
Subsystem	Oxidative phosphorylation
EcoCyc	http://www.ecocyc.org/ECOLI/NEW-IMAGE?type=REACTION&object=NADH-DEHYDROG-A-RXN
Compartment	cm
Flux from	thermokinetic law (R>0)

nadp

Type	Compound
Name	Nicotinamide dinucleotide phosphate
Formula	C21H25N7O17P3
Charge	-3
Compartment	c

nadph

Type	Compound
Name	Nicotinamide adenine dinucleotide phosphate - reduced
Formula	C21H26N7O17P3
Charge	-4
Compartment	c

nadpsyn

Type	Flux
Name	de novo synthesis of nadp
Reactants	−3+
Products	nadp
Compartment	c
Flux from	conventional kinetic law

Comment: de novo synthesis of nadp is adjusted such that the total concentration nadp+nadph is approx. constant

nadsyn

Type	Flux
Name	de novo synthesis of nad
Reactants	−+
Products	nad
Compartment	c
Flux from	conventional kinetic law

Comment: de novo synthesis of nad is adjusted such that the total concentration nadh+nad is approx. constant

o2

Type	Compound
Name	O2
Formula	O2
Charge	0
Compartment	c

o2(e)

Type	Compound
Name	O2 (e)
Formula	O2
Charge	0
Compartment	e

O2:e->p

Type	Flux
Name	oxygen transport through periplasmatic membrane
Reactants	o2(e)
Products	o2(p)
Subsystem	Transport, Extracellular
Compartment	om
Flux from	thermokinetic law (R>0)

O2:in

Type	Flux
Name	oxygen in/out
Reactants	0
Products	o2(e)
Subsystem	in- and outflow of oxygen into/out of the reactor
Compartment	e
Flux from	conventional kinetic law

o2(p)

Type	Compound
Name	O2 (p)
Formula	O2
Charge	0
Compartment	p

O2t

Type	Flux
Name	o2 transport through cytoplasmatic membrane
Reactants	o2(p)
Products	o2
Subsystem	Transport, Extracellular
Compartment	cm
Flux from	thermokinetic law (R>0)

oaa

Type	Compound
Name	Oxaloacetate
Formula	C4H2O5
Charge	-2
Compartment	c

om

Type	Compartment
Name	outer membrane
Temperature	310.15 K
pH	7.6
IonicStrength	0.15 mM

p

Type	Compartment
Name	periplasm
Temperature	310.15 K
pH	7.6
IonicStrength	0.15 mM

p+

Type	Compartment
Name	charged boundary layerin periplasm atcytoplasmatic membrane
Temperature	310.15 K
pH	7.6
IonicStrength	0.15 mM

PDH

Type	Flux
Name	pyruvate dehydrogenase
EC	1.2.1
Reactants	coa + nad + pyr
Products	accoa + co2 + nadh
Subsystem	Glycolysis/Gluconeogenesis
EcoCyc	http://biocyc.org/ECOLI/NEW-IMAGE?type=REACTION&object=PYRUVDEH-RXN
Compartment	c
Flux from	thermokinetic law (R>0)

PdhR

Type	Compound
Name	Transcription Factor PdhR (unmodified form)
Charge	0
EcoCyc	http://biocyc.org/ECOLI/NEW-IMAGE?type=ENZYME&object=EG11088-MONOMER
Compartment	c

Comment: PdhR + pyruvate = PdhR-pyruvate; PdhR is Repressor

pep

Type	Compound
Name	Phosphoenolpyruvate
Formula	C3H2O6P
Charge	-3
Compartment	c

PFK

Type	Flux
Name	phosphofructokinase
EC	2.7.1.11
Reactants	atp + f6p
Products	adp + fdp + h
Subsystem	Glycolysis/Gluconeogenesis
EcoCyc	http://biocyc.org/ECOLI/NEW-IMAGE?type=REACTION&object=6PFRUCTPHOS-RXN
Compartment	c
Flux from	thermokinetic law (R>0)

PFL

Type	Flux
Name	pyruvate formate lyase
EC	2.3.1.54
Reactants	coa + pyr
Products	accoa + for
Subsystem	Pyruvate metabolism
EcoCyc	http://biocyc.org/ECOLI/NEW-IMAGE?type=REACTION&object=PYRUVFORMLY-RXN
Compartment	c
Flux from	thermokinetic law (R>0)

PGI

Type	Flux
Name	glucose-6-phosphate isomerase
EC	5.3.1.9
Reactants	g6p
Products	f6p
Subsystem	Glycolysis/Gluconeogenesis
EcoCyc	http://biocyc.org/ECOLI/NEW-IMAGE?type=REACTION&object=PGLUCISOM-RXN
Compartment	c
Flux from	rapid equilibrium (R=0)

PGK

Type	Flux
Name	phosphoglycerate kinase
EC	2.7.2.3
Reactants	3pg + atp
Products	13dpg + adp
Subsystem	Glycolysis/Gluconeogenesis
EcoCyc	http://biocyc.org/ECOLI/NEW-IMAGE?type=REACTION&object=PHOSGLYPHOS-RXN
Compartment	c
Flux from	rapid equilibrium (R=0)

PGL

Type	Flux
Name	6-phosphogluconolactonase
EC	3.1.1.31
Reactants	6pgl + h2o
Products	6pgc + h
Subsystem	Pentose Phosphate Cycle
EcoCyc	http://biocyc.org/ECOLI/NEW-IMAGE?type=REACTION&object=6PGLUCONOLACT-RXN
Compartment	c
Flux from	rapid equilibrium (R=0)

PGM

Type	Flux
Name	phosphoglycerate mutase
EC	5.4.2.1
Reactants	2pg
Products	3pg
Subsystem	Glycolysis/Gluconeogenesis
EcoCyc	http://biocyc.org/ECOLI/NEW-IMAGE?type=REACTION&object=3PGAREARR-RXN
Compartment	c
Flux from	rapid equilibrium (R=0)

pi

Type	Compound
Name	Phosphate
Formula	HO4P
Charge	-2
Compartment	c

POX

Type	Flux
Name	pyruvate oxidase
EC	1.2.5.1
Reactants	h2o + pyr + q8
Products	ac + co2 + q8h2
Subsystem	oxidative phophorylation
EcoCyc	http://biocyc.org/ECOLI/NEW-IMAGE?type=REACTION&object=RXN-11496
Compartment	c
Flux from	thermokinetic law (R>0)

PPA

Type	Flux
Name	inorganic pyrophosphatase
EC	3.6.1.1
Reactants	h2o + ppi
Products	h + 2pi
EcoCyc	http://biocyc.org/ECOLI/NEW-IMAGE?type=REACTION&object=INORGPYROPHOSPHAT-RXN
Compartment	c
Flux from	rapid equilibrium (R=0)

PPC

Type	Flux
Name	phosphoenolpyruvate carboxylase
EC	4.1.1.31
Reactants	co2 + h2o + pep
Products	h + oaa + pi
Subsystem	Anaplerotic reactions
EcoCyc	http://biocyc.org/ECOLI/NEW-IMAGE?type=REACTION&object=PEPCARBOX-RXN
Compartment	c
Flux from	thermokinetic law (R>0)

PPCK

Type	Flux
Name	phosphoenolpyruvate carboxykinase
EC	4.1.1.49
Reactants	atp + oaa
Products	adp + co2 + pep
Subsystem	Anaplerotic reactions
EcoCyc	http://biocyc.org/ECOLI/NEW-IMAGE?type=REACTION&object=PEPCARBOXYKIN-RXN
Compartment	c
Flux from	thermokinetic law (R>0)

ppi

Type	Compound
Name	diphosphate
Formula	HO7P2
Charge	-3
Compartment	c

PPS

Type	Flux
Name	phosphoenolpyruvate synthase
EC	2.7.9.2
Reactants	atp + h2o + pyr
Products	amp + 2h + pep + pi
Subsystem	Glycolysis/Gluconeogenesis
EcoCyc	http://biocyc.org/ECOLI/NEW-IMAGE?type=REACTION&object=PEPSYNTH-RXN
Compartment	c
Flux from	thermokinetic law (R>0)

PTAr

Type	Flux
Name	phosphotransacetylase
EC	2.3.1.8
Reactants	accoa + pi
Products	actp + coa
Subsystem	Pyruvate metabolism
EcoCyc	http://biocyc.org/ECOLI/NEW-IMAGE?type=REACTION&object=PHOSACETYLTRANS-RXN
Compartment	c
Flux from	thermokinetic law (R>0)

PYK

Type	Flux
Name	pyruvate kinase
EC	2.7.1.40
Reactants	adp + h + pep
Products	atp + pyr
Subsystem	Glycolysis/Gluconeogenesis
EcoCyc	http://biocyc.org/ECOLI/NEW-IMAGE?type=REACTION&object=PEPDEPHOS-RXN
Compartment	c
Flux from	thermokinetic law (R>0)

pyr

Type	Compound
Name	Pyruvate
Formula	C3H3O3
Charge	-1
Compartment	c

q8

Type	Compound
Name	Ubiquinone-8
Formula	C49H74O4
Charge	0
Compartment	cm

q8*

Type	Compound
Name	Ubiquinone-8 (active and inactive)
Formula	C49H74O4
Charge	0
Compartment	cm

Comment: In order to reproduce the observation that even in the complete anaerobiccase a substantial part of the quinone pool is oxidized, we need tointroduce a constant pool of oxidized quinones that does not participatein any reaction.Concentration is calculated as the sum of the active and an assumed inactive formwith constant concentration. The inactive form does not participate in any reactions.

q8h2

Type	Compound
Name	Ubiquinol-8
Formula	C49H76O4
Charge	0
Compartment	cm

q8h2*

Type	Compound
Name	Ubiquinol-8 (active and inactive)
Formula	C49H74O4
Charge	0
Compartment	cm

Comment: Concentration is calculated as the sum of the active and an assumed inactive form.

q8syn

Type	Flux
Name	de novo synthesis of q8
Reactants	0
Products	q8h2
Compartment	cm
Flux from	conventional kinetic law

Comment: de novo synthesis of q8 inreases linearily with aerobiosis

r5p

Type	Compound
Name	alpha-D-Ribose 5-phosphate
Formula	C5H9O8P
Charge	-2
Compartment	c

RPE

Type	Flux
Name	ribulose 5-phosphate 3-epimerase
EC	5.1.3.1
Reactants	ru5p-D
Products	xu5p-D
Subsystem	Pentose Phosphate Cycle
EcoCyc	http://biocyc.org/ECOLI/NEW-IMAGE?type=REACTION&object=RIBULP3EPIM-RXN
Compartment	c
Flux from	rapid equilibrium (R=0)

RPI

Type	Flux
Name	ribose-5-phosphate isomerase
EC	5.3.1.6
Reactants	r5p
Products	ru5p-D
Subsystem	Pentose Phosphate Cycle
EcoCyc	http://biocyc.org/ECOLI/NEW-IMAGE?type=REACTION&object=RIB5PISOM-RXN
Compartment	c
Flux from	rapid equilibrium (R=0)

ru5p-D

Type	Compound
Name	D-Ribulose 5-phosphate
Formula	C5H9O8P
Charge	-2
Compartment	c

s7p

Type	Compound
Name	Sedoheptulose 7-phosphate
Formula	C7H13O10P
Charge	-2
Compartment	c

succ

Type	Compound
Name	Succinate
Formula	C4H4O4
Charge	-2
Compartment	c

succ(e)

Type	Compound
Name	Succinate (e)
Formula	C4H4O4
Charge	-2
Compartment	e

SUCC:e->p

Type	Flux
Name	succinate transport through periplasmatic membrane
Reactants	succ(e)
Products	succ(p)
Subsystem	Transport, Extracellular
Compartment	om
Flux from	rapid equilibrium (R=0)

succoa

Type	Compound
Name	Succinyl-CoA
Formula	C25H35N7O19P3S
Charge	-5
Compartment	c

succ(p)

Type	Compound
Name	Succinate (periplasm)
Formula	C4H4O4
Charge	-2
Compartment	p

SUCCt

Type	Flux
Name	succinate transport through cytoplasmatic membrane
Reactants	h(p+)P(SUCCt_h) + succ
Products	hP(SUCCt_h) + succ(p)
Subsystem	Transport, Extracellular
DOI	http://dx.doi.org/10.1111/j.1432-1033.1994.tb18903.x
Compartment	cm
Flux from	thermokinetic law (R>0)

SUCDH

Type	Flux
Name	succinate dehydrogenase
EC	1.3.5.1
Reactants	q8 + succ
Products	fum + q8h2
Subsystem	Citrate Cycle (TCA)
EcoCyc	http://biocyc.org/ECOLI/NEW-IMAGE?type=REACTION&object=SUCCINATE-DEHYDROGENASE-UBIQUINONE-RXN
Compartment	cm
Flux from	thermokinetic law (R>0)

SUCOAS

Type	Flux
Name	succinyl-CoA synthetase (ADP-forming)
EC	6.2.1.5
Reactants	atp + coa + succ
Products	adp + pi + succoa
Subsystem	Citrate Cycle (TCA)
EcoCyc	http://biocyc.org/ECOLI/NEW-IMAGE?type=REACTION&object=SUCCCOASYN-RXN
Compartment	c
Flux from	rapid equilibrium (R=0)

TALA

Type	Flux
Name	transaldolase
EC	2.2.1.2
Reactants	g3p + s7p
Products	e4p + f6p
Subsystem	Pentose Phosphate Cycle
EcoCyc	http://biocyc.org/ECOLI/NEW-IMAGE?type=REACTION&object=TRANSALDOL-RXN
Compartment	c
Flux from	rapid equilibrium (R=0)

THD-PntAB

Type	Flux
Name	NADH transhydrogenase
EC	1.6.1.2
Reactants	2h + nad + nadph
Products	2h(e) + nadh + nadp
Subsystem	Oxidative Phosphorylation
EcoCyc	http://www.ecocyc.org/ECOLI/NEW-IMAGE?type=REACTION&object=TRANS-RXN0-277
Compartment	cm
Flux from	thermokinetic law (R>0)

THD-SthA

Type	Flux
Name	NADH transhydrogenase
EC	1.6.1.1
Reactants	nad + nadph
Products	nadh + nadp
Subsystem	Oxidative Phosphorylation
EcoCyc	http://www.ecocyc.org/ECOLI/NEW-IMAGE?type=REACTION&object=PYRNUTRANSHYDROGEN-RXN
Compartment	c
Flux from	thermokinetic law (R>0)

TKT1

Type	Flux
Name	transketolase
EC	2.2.1.1
Reactants	r5p + xu5p-D
Products	g3p + s7p
Subsystem	Pentose Phosphate Cycle
EcoCyc	http://biocyc.org/ECOLI/NEW-IMAGE?type=REACTION&object=1TRANSKETO-RXN
Compartment	c
Flux from	rapid equilibrium (R=0)

TKT2

Type	Flux
Name	transketolase
EC	2.2.1.1
Reactants	e4p + xu5p-D
Products	f6p + g3p
Subsystem	Pentose Phosphate Cycle
EcoCyc	http://biocyc.org/ECOLI/NEW-IMAGE?type=REACTION&object=2TRANSKETO-RXN
Compartment	c
Flux from	rapid equilibrium (R=0)

TPI

Type	Flux
Name	triose-phosphate isomerase
EC	5.3.1.1
Reactants	dhap
Products	g3p
Subsystem	Glycolysis/Gluconeogenesis
EcoCyc	http://biocyc.org/ECOLI/NEW-IMAGE?type=REACTION&object=TRIOSEPISOMERIZATION-RXN
Compartment	c
Flux from	rapid equilibrium (R=0)

xu5p-D

Type	Compound
Name	D-Xylulose 5-phosphate
Formula	C5H9O8P
Charge	-2
Compartment	c
