# Supplementary material for: A mathematical model of metabolism and regulation provides a systems-level view of how Escherichia coli responds to oxygen
Source: Front Microbiol. 2014 Mar 27;5:124. doi: 10.3389/fmicb.2014.00124 (PMC3973912; doi:10.3389/fmicb.2014.00124)
Supplement: Supplementary Data Sheet 2 — Model Definition File. [file DataSheet2.PDF]

```
(* ************************************************************************************ *)
(* Thermokinetic Model of the oxygen response of Escherichia coli                       *)
(* ************************************************************************************ *)
(*                                                                                      *)
(* Author: Michael Ederer, ederer@isys.uni-stuttgart.de                                 *)
(*                                                                                      *)
(*    This file is an input file for the  Mathematica package TKMOD.                    *)
(*    It should be viewed and edited with a text editor                                 *)
(*                                                                                      *)
(* ************************************************************************************ *)

Needs["Units`"];

(* *** Define aliases for greek letters for easier typing                           *** *)
Phi=\[Phi];
Phi0=\[Phi]0
Mu=\[Mu];
data`Mu0=data`\[Mu]0;
Mu0=\[Mu]0;
Mu01=\[Mu]01;
Mu02=\[Mu]02;
Xi=\[Xi];

Module[{net},
       (* *** Temperature, presssure, pH and ionic strength                         *** *)
       p = 101325  (* Pascal *);
       pH = 7.6;
       is = 0.15;


       (* *** Geometry of an Escherichia coli cell                                  *** *)
       (* Source: http://redpoll.pharmacy.ualberta.ca/CCDB/cgi-bin/STAT_NEW.cgi *)
       (* volume of cytoplasm:               *)  Vc  =  6.7*10^(-16) (*Liter*);
       (* volume of periplasm:               *)  Vp  =  6.5*10^(-17) (*Liter*);
       (* surface of cytoplasmatic membrane: *)  Acm =  1.0*6  *10^(-16) (*Meter^2*);
       (* Inner Membrane thickness           *)  hcm =  8*10^(-9) (*Meter*);
       (* surface of periplasmatic membrane: *)  Apm =  6  *10^(-16) (*Meter^2*);
       (* dry cell weight (single cell):     *)  DCW =  P["DCW"] (*Gram*);


       (* *** Thermodynamic data of metabolites                                     *** *)
       (* Thermodynamic data for many metabolites *)
       (* Source:  Alberty R.A.; 2003. Thermodynamics of Biochemical Reactions.  Wiley. *)
       (* Data extracted from  http://library.wolfram.com/infocenter/Demos/5704/ *)
       <<"MetaboliteMu0.mma";

       (* Thermodynamic data for quinones (from [Alvarez2013])*)

       data`Mu0["mqn8"] = 0;
       data`Mu0["mql8"] = 0.074*F/1000; (* Em = -74mV *)
       data`Mu0["q8"] = 0;
       data`Mu0["q8h2"] = -0.100*F/1000; (* Em = +100mV *)
       (* Thermodynamic data for compounds in the PPP *)
       (* Source: Kümmel A., Panke S. and Heinemann M.; 2006.
          Systematic assignment of thermodynamic constraints in metabolic network models.
          BMC Bioinformatics. 7: 512. http://dx.doi.org/10.1186/1471-2105-7-512 *)
       data`Mu0["6pgc"] = -1535.64;
       data`Mu0["e4p"] = -1125.83;
       data`Mu0["2ddg6p"] = -1428.76;
       data`Mu0["s7p"] = -1336.14;
       data`Mu0["xu5p-D"] = -1203.1; (* The value is for xu5p-L*)

       (* **** Legendre Transformation for constant water activity                  *** *)
```

```
(* Source:  Alberty R.A.; 2003. Thermodynamics of Biochemical Reactions.  Wiley. *)
Mu0[n_]  := Mu02[n];
Mu02[n_] := Mu01[n] - NO[n] (Mu01["h2o"]);
Mu01[n_] := data‘Mu0[n] 10^3 ;
NO[n_String] := NE[n, "O"];

(* *** Computation of thermokinetic capacities                              *** *)
(*Unit of conc: mol/L*)
Cap[m_] := 1*Exp[-Mu0[m] / (R*T[Compartment[m]][t])];

(* *** Initial estimates for electrical potentials                          *** *)
Phi0["c"] := 0;
Phi0["cm"] := 0.7005;
Phi0["pm"] := 0;
Phi0["p"] := 0;
Phi0["p+"] := 0.1401;
Phi0["e"] := 0;


(* *** Conversion factors from concentrations into thermokinetic potentials      *** *)
unit[m_] :=  1/Cap[m]*Exp[Charge[m]*F*Phi0[Compartment[m]]/(R*T[Compartment[m]][t])];
mM[m_] := 1/1000*unit[m];
Molar[m_] := unit[m];


(* *** Initial conditions of population size                                *** *)
(* initial number of cells*)
n0 = (10^12);
n0 = P["DCW_IC"]/DCW;
Vc0  = n0*Vc;
Vp0  = n0*Vp;
Acm0 = n0*Acm;
Apm0 = n0*Apm;
DCW0 = n0*DCW;


(* *** Auxiliary function for gene regulation                               *** *)
(* Parameter GENREG allows to switch of gene regulation completely *)
s[a_,x_]:=(2^(-a)+((1-2^(-a)) /. P[n_,___]:>P[n])*x)*P["GENREG"]+0.5*(1-P["GENREG"]);


(* *** Parameters for Quasi-steady trajectory                               *** *)
t0=200.;
t1=50200.0;
t2=100200;

(* *** Constants                                                            *** *)
L=(AvogadroConstant*Mole);

(* *** Network description                                                  *** *)
net=
NetworkDescription["Escherichia coli",
                   {
                       (* *** Some central parameters                       *** *)
                       P["DIL","Value"->0.2],
                       P["GENREG","Value"-> 1.0],
                       TEMP=Exp[P["ln(T)"]];
                       P["ln(T)", "Value"->Log[273.15 + 37],"TeXExport"->False],
                       P["DCW_IC", "Value"->1.64],
                       P["DCW", "Value" -> 2.5*^-13],
                       P["MolarMassO2", "Value"-> 31.999],
                       P["Salinity", "Value" -> 6,
                         "Comment"-> "Salinity of medium in g/kg"],
```

```
(* *** Compartments                                         *** *)
Compartment["c",
            "Name"                 -> "cytoplasm",
            "SizeDot"              -> (J["GROWTH"][t]*V["c"][t]
                                       -P["DIL"]*V["c"][t]),
            "Size"                 -> Vc0,
            "Dilution"             -> J["GROWTH"][t],
            "ElectricalPotential" -> 0,
            "pH"                   -> 7.6,
            "IonicStrength"        -> 0.15,
            "Temperature"          -> TEMP],
Compartment["cm",
            "Name"                 -> "cytoplasmatic membrane",
            "SizeDot"              -> (J["GROWTH"][t]*V["cm"][t]
                                       -P["DIL"]*V["cm"][t]),
            "Size"                 -> Acm0,
            "Dilution"             -> J["GROWTH"][t],
            "ElectricalPotential" -> (Phi["c"][t]+
                                       Phi["p+"][t])/2,
            "pH"                   -> 7.6,
            "IonicStrength"        -> 0.15,
            "Temperature"          -> TEMP],
Compartment["p+",
            "Name"                 -> ("charged boundary layer"
                                       <> "in periplasm at" <>
                                       "cytoplasmatic membrane"),
            "SizeDot"              -> (J["GROWTH"][t]*V["p+"][t]
                                       -P["DIL"]*V["p+"][t]),
            "Size"                 -> Acm0,
            "Dilution"             -> J["GROWTH"][t],
            "ElectricalPotential" -> ((c["h(p+)"][t]-
                                        c["h"][t])/
                                       P["Cel",
                                         "Value"-> 0.2*^-8]),
            "pH"                   -> 7.6,
            "IonicStrength"        -> 0.15,
            "Temperature"          -> TEMP],
Compartment["p",
            "Name"                 -> "periplasm",
            "SizeDot"              -> (J["GROWTH"][t]*V["p"][t]
                                       -P["DIL"]*V["p"][t]),
            "Size"                 -> Vp0,
            "Dilution"             -> J["GROWTH"][t],
            "ElectricalPotential" -> 0,
            "pH"                   -> 7.6,
            "IonicStrength"        -> 0.15,
            "Temperature"          -> TEMP],
Compartment["om",
            "Name"                 -> "outer membrane",
            "SizeDot"              -> (J["GROWTH"][t]*V["om"][t]
                                       -P["DIL"]*V["om"][t]),
            "Size"                 -> Apm0,
            "Dilution"             -> J["GROWTH"][t],
            "ElectricalPotential" -> 0,
            "pH"                   -> 7.6,
            "IonicStrength"        -> 0.15,
            "Temperature"          -> TEMP],
Compartment["e",
            "Name"                 -> "cell exterior (medium)",
            "SizeDot"              -> 0,
            "Size"                 -> 1, (*Liter*)
            "ElectricalPotential" -> 0,
```

```
            "pH"                    -> 7.6,
            "IonicStrength"         -> 0.15,
            "Dilution"              -> P["DIL"],
            "Temperature"           -> TEMP],
(* *** Compounds                                        *** *)
Compound["+",
         "Compartment"          -> "e",
         "Name"                 -> "elementary charge",
         "Comment"              -> ("This is a dummy compound."<>
                                     "It is used for charge" <>
                                     "balancing in de novo" <>
                                     "synthesis reactions."<>
                                     "This avoids warning" <>
                                     "messages. It has no" <>
                                     "influence on the model" <>
                                     "results."),
         "Formula"              -> "",
         "Charge"               -> 1,
         "Capacity"             -> 1,
         "Potential"            -> 1,
         "Scale"                -> 1,
         "Clamped"              -> True],
Compound["13dpg",
         "Compartment"          -> "c",
         "Name"                 -> "3-Phospho-D-glyceroyl phosphate",
         "Formula"              -> "C3H4O10P2",
         "Charge"               -> -4,
         "Capacity"             -> Cap["13dpg"],
         "Potential"            -> P["13dpg_IC", "Value"->0.12797877832902533547*^-00003] Molar["13dpg"],
         "Scale"                -> 0.3 *10^(-3) Molar["13dpg"] ],
Compound["2ddg6p",
         "Compartment"          -> "c",
         "Name"                 -> "2-Dehydro-3-deoxy-D-gluconate 6-phosphate",
         "Formula"              -> "C6H8O9P",
         "Charge"               -> -3,
         "Link"                 -> {"EcoCyc" ->
                                     "http://biocyc.org/ECOLI/NEW-IMAGE?type=COMPOUND-IN-PATHWAY&object=2-KETO-3-DEOXY-6-P-GLUCONATE"},
         "Capacity"             -> Cap["2ddg6p"],
         "Potential"            -> P["2ddg6p_IC", "Value"->0.12933583344651234008*^-00008] Molar["2ddg6p"],
         "Scale"                -> 1 *10^(-3) Molar["2ddg6p"]
        ],
Compound["2pg",
         "Compartment"          -> "c",
         "Name"                 -> "D-Glycerate 2-phosphate",
         "Formula"              -> "C3H4O7P",
         "Charge"               -> -3,
         "Capacity"             -> Cap["2pg"],
         "Potential"            -> P["2pg_IC", "Value"->0.88835804944886731776*^-00004] Molar["2pg"],
         "Scale"                -> 0.3 *10^(-3) Molar["2pg"] ],
Compound["3pg",
         "Compartment"          -> "c",
         "Name"                 -> "3-Phospho-D-glycerate",
         "Formula"              -> "C3H4O7P",
         "Charge"               -> -3,
         "Capacity"             -> Cap["3pg"],
         "Scale"                -> 3.5 *10^(-3) Molar["3pg"],
         "Potential"            -> P["3pg_IC", "Value"->0.93304302219623903251*^-00003] Molar["3pg"]],
Compound["6pgc",
         "Compartment"          -> "c",
         "Name"                 -> "6-Phospho-D-gluconate",
         "Formula"              -> "C6H10O10P",
         "Charge"               -> -3,
         "Capacity"             -> Cap["6pgc"],
```

```
          "Scale"              -> 3.5 *10^(-3) Molar["6pgc"],
          "Potential"          -> P["6pgc_IC", "Value"->0.16042670085310667497*^-00004] Molar["6pgc"]],
Compound["6pgl",
          "Compartment"        -> "c",
          "Name"               -> "6-phospho-D-glucono-1,5-lactone",
          "Formula"            -> "C6H9O9P",
          "Charge"             -> -2,
          "Capacity"           -> Cap["6pgl"],
          "Scale"              -> 3.5 *10^(-3) Molar["6pgl"],
          "Potential"          -> P["6pgl_IC", "Value"->0.25927944215064626529*^-00010] Molar["6pgl"]],
Compound["ac",
          "Compartment"        -> "c",
          "Name"               -> "Acetate",
          "Formula"            -> "C2H3O2",
          "Charge"             -> -1,
          "Capacity"           -> Cap["ac"],
          "Scale"              -> 1 *10^(-3) Molar["ac"]  ,
          "Potential"          -> P["ac_IC", "Value"->0.53857964730750542955*^-00005] Molar["ac"]],
Compound["ac(e)",
          "Compartment"        -> "e",
          "Name"               -> "Acetate (e)",
          "Formula"            -> "C2H3O2",
          "Charge"             -> -1,
          "Capacity"           -> Cap["ac(e)"],
          "ReductionPriority"  -> 100,
          "Scale"              -> 0.1 *10^(-3) Molar["ac(e)"]  ,
          "Potential"          -> P["ac(e)_IC", "Value"->0.10298228079130351993*^-00002] Molar["ac(e)"]],
Compound["ac(p)",
          "Compartment"        -> "p",
          "Name"               -> "Acetate (p)",
          "Formula"            -> "C2H3O2",
          "Charge"             -> -1,
          "Capacity"           -> Cap["ac(p)"],
          "Scale"              -> 0.001 *10^(-3) Molar["ac(p)"]  ,
          "Potential"          -> P["ac(p)_IC", "Value"->0.10298228079130351993*^-00002] Molar["ac(p)"]],
Compound["accoa",
          "Compartment"        -> "c",
          "Name"               -> "Acetyl-CoA",
          "Formula"            -> "C23H34N7O17P3S",
          "Charge"             -> -4,
          "Capacity"           -> Cap["accoa"],
          "Scale"              -> 0.5 *10^(-3) Molar["accoa"],
          "ReductionPriority"  -> 100,
          "Potential"          -> P["accoa_IC", "Value"->0.44020487937556502653*^-00007] Molar["accoa"]],
Compound["actp",
          "Compartment"        -> "c",
          "Name"               -> "Acetyl phosphate",
          "Formula"            -> "C2H3O5P",
          "Charge"             -> -2,
          "Capacity"           -> Cap["actp"],
          "Scale"              -> 1 *10^(-3) Molar["actp"]  ,
          "Potential"          -> P["actp_IC", "Value"->0.29082142460690300784*^-00006] Molar["actp"]],
Compound["akg",
          "Compartment"        -> "c",
          "Name"               -> "2-Oxoglutarate",
          "Formula"            -> "C5H4O5",
          "Charge"             -> -2,
          "Capacity"           -> Cap["akg"],
          "Scale"              -> 0.5 *10^(-3) Molar["akg"],
          "Potential"          -> P["akg_IC", "Value"->0.14818657699202068252*^-00005] Molar["akg"]],
Compound["amp",
          "Compartment"        -> "c",
          "Name"               -> "AMP",
```

```
                 "Formula"              -> "C10H12N5O7P",
                 "Charge"               -> -2,
                 "Capacity"             -> Cap["amp"],
                 "Scale"                -> 1 *10^(-3) Molar["amp"],
                 "ReductionPriority"    -> 0,
                 "Potential"            -> P["amp_IC", "Value"->0.26223220151889393769*^-00004] Molar["amp"]],
        Compound["adp",
                 "Compartment"          -> "c",
                 "Name"                 -> "ADP",
                 "Formula"              -> "C10H12N5O10P2",
                 "Charge"               -> -3,
                 "Capacity"             -> Cap["adp"],
                 "Scale"                -> 1 *10^(-3) Molar["adp"],
                 "ReductionPriority"    -> 100,
                 "Potential"            -> P["adp_IC", "Value"->0.21703182383337656113*^-00003] Molar["adp"]],
        Compound["atp",
                 "Compartment"          -> "c",
                 "Name"                 -> "ATP",
                 "Formula"              -> "C10H12N5O13P3",
                 "Charge"               -> -4,
                 "Capacity"             -> Cap["atp"],
                 "Scale"                -> 1.9 *10^(-3) Molar["atp"],
                 "ReductionPriority"    -> 100,
                 "Potential"            -> P["atp_IC", "Value"->0.73713702675401232209*^-00003] Molar["atp"]],
        Flux["ampsyn",
             "Reactants"                -> -2 "+",
             "Products"                 -> "amp",
             "Compartment"              -> "c",
             "Name"                     -> "de novo synthesis of amp",
             "Clamped"                  -> True,
             "Comment"                  -> "de novo synthesis of amp is adjusted such that the total concentration amp+adp+atp is approx. constant",
             "Flux"                     -> -P["ampsyn_vmax","Value"-> 10]*(c["amp"][t]+c["adp"][t]+c["atp"][t]-10^(-3)*P["amp_tot","Value"-> 1.0])],
        Compound["cit",
                 "Compartment"          -> "c",
                 "Name"                 -> "Citrate",
                 "Formula"              -> "C6H5O7",
                 "Charge"               -> -3,
                 "Capacity"             -> Cap["cit"],
                 "Potential"            -> P["cit_IC", "Value"->0.13503583242722769391*^-00007] Molar["cit"],
                 "Scale"                -> 10 *10^(-3) Molar["cit"]],
        Compound["co2",
                 "Compartment"          -> "c",
                 "Name"                 -> "CO2",
                 "Formula"              -> "CO2",
                 "Charge"               -> 0,
                 "Capacity"             -> Cap["co2"],
                 "Scale"                -> 20 *10^(-3) Molar["co2"],
                 "Potential"            -> P["co2_IC", "Value"->0.10975297480813888014*^-00002] Molar["co2"]],
        Compound["co2(e)",
                 "Compartment"          -> "e",
                 "Name"                 -> "CO2 (e)",
                 "Formula"              -> "CO2",
                 "Charge"               -> 0,
                 "Capacity"             -> Cap["co2(e)"],
                 "Clamped"              -> False,
                 "ReductionPriority"    -> 100,
                 "Scale"                -> 20 *10^(-3) Molar["co2(e)"],
                 "Potential"            -> P["co2(e)_IC", "Value"->0.10975297480813888014*^-00002] Molar["co2(e)"]],
        Compound["co2(p)",
                 "Compartment"          -> "p",
                 "Name"                 -> "CO2 (p)",
                 "Formula"              -> "CO2",
                 "Charge"               -> 0,
```

```
          "Capacity"           -> Cap["co2(p)"],
          "Scale"              -> 20 *10^(-3) Molar["co2(p)"]  ,
          "Potential"          -> P["co2(p)_IC", "Value"->0.10975297480813877172*^-00002] Molar["co2(p)"]],
Compound["coa",
          "Compartment"        -> "c",
          "Name"               -> "Coenzyme A",
          "Formula"            -> "C21H32N7O16P3S",
          "Charge"             -> -4,
          "Capacity"           -> Cap["coa"],
          "Scale"              -> 0.1 *10^(-3) Molar["coa"],
          "ReductionPriority"  -> 100,
          "Potential"          -> P["coa_IC", "Value"->0.96816481143433127596*^-00003] Molar["coa"]],
Flux["coasyn",
     "Reactants"               -> -4"+",
     "Products"                -> "coa",
     "Compartment"             -> "c",
     "Comment"                 -> "de novo synthesis of coa is adjusted such that the total concentration coa+succoa+accoa is approx. constant"
,
     "Name"                    -> "de novo synthesis of coa",
     "Clamped"                 -> True,
     "Flux"                    -> -P["coasyn_vmax","Value"-> 10]*(c["coa"][t]+c["accoa"][t]+c["succoa"][t]-10^(-3)*P["coa_tot","Value"-> 1])],
Compound["dhap",
          "Compartment"        -> "c",
          "Name"               -> "Dihydroxyacetone phosphate",
          "Formula"            -> "C3H5O6P",
          "Charge"             -> -2,
          "Capacity"           -> Cap["dhap"],
          "Scale"              -> 0.6 *10^(-3) Molar["dhap"],
          "Potential"          -> P["dhap_IC", "Value"->0.13976378787801187913*^-00003] Molar["dhap"]],
Compound["e4p",
          "Compartment"        -> "c",
          "Name"               -> "D-Erythrose 4-phosphate",
          "Formula"            -> "C4H7O7P",
          "Charge"             -> -2,
          "Capacity"           -> Cap["e4p"],
          "Scale"              -> 1 *10^(-3) Molar["e4p"]  ,
          "Potential"          -> P["e4p_IC", "Value"->0.16271158025916373924*^-00007] Molar["e4p"]],
Compound["etoh",
          "Compartment"        -> "c",
          "Name"               -> "Ethanol",
          "Formula"            -> "C2H6O",
          "Charge"             -> 0,
          "Capacity"           -> Cap["etoh"],
          "Scale"              -> 1 *10^(-3) Molar["etoh"]  ,
          "Potential"          -> P["etoh_IC", "Value"->0.28009631859145161360*^-00011] Molar["etoh"]],
Compound["etoh(p)",
          "Compartment"        -> "p",
          "Name"               -> "Ethanol",
          "Formula"            -> "C2H6O",
          "Charge"             -> 0,
          "Capacity"           -> Cap["etoh(p)"],
          "Scale"              -> 1 *10^(-3) Molar["etoh(p)"]  ,
          "Potential"          -> P["etoh(p)_IC", "Value"->0.53557459651509610347*^-00009] Molar["etoh(p)"]],
Compound["etoh(e)",
          "Compartment"        -> "e",
          "Name"               -> "Ethanol",
          "Formula"            -> "C2H6O",
          "Charge"             -> 0,
          "ReductionPriority"  -> 100,
          "Capacity"           -> Cap["etoh(e)"],
          "Scale"              -> 1 *10^(-3) Molar["etoh(e)"]  ,
          "Potential"          -> P["etoh(e)_IC", "Value"->0.53557459651509558648*^-00009]Molar["etoh(e)"]],
Compound["f6p",
```

```
              "Compartment"         -> "c",
              "Name"                -> "D-Fructose 6-phosphate",
              "Formula"             -> "C6H11O9P",
              "Charge"              -> -2,
              "Capacity"            -> Cap["f6p"],
              "Scale"               -> 0.1 *10^(-3) Molar["f6p"]  ,
              "Potential"           -> P["f6p_IC", "Value"->0.16384349886873104090*^-00005] Molar["f6p"]],
     Compound["fdp",
              "Compartment"         -> "c",
              "Name"                -> "D-Fructose 1,6-bisphosphate",
              "Formula"             -> "C6H10O12P2",
              "Charge"              -> -4,
              "Capacity"            -> Cap["fdp"],
              "Scale"               -> 1 *10^(-3) Molar["fdp"],
              "ReductionPriority"   -> 100,
              "Potential"           -> P["fdp_IC", "Value"->0.53321119491905173921*^-00005] Molar["fdp"]],
     Compound["for",
              "Compartment"         -> "c",
              "Name"                -> "Formate",
              "Formula"             -> "CH1O2",
              "Charge"              -> -1,
              "Capacity"            -> Cap["for"],
              "Scale"               -> 1 *10^(-3) Molar["for"]  ,
              "Potential"           -> P["for_IC", "Value"->0.17806976303432372678*^-00005] Molar["for"]],
     Compound["for(e)",
              "Compartment"         -> "e",
              "Name"                -> "Formate (e)",
              "Formula"             -> "CH1O2",
              "Charge"              -> -1,
              "ReductionPriority"   -> 100,
              "Capacity"            -> Cap["for(e)"],
              "Scale"               -> 1 *10^(-3) Molar["for(e)"]  ,
              "Potential"           -> P["for(e)_IC", "Value"->0.34048873604709063755*^-00003] Molar["for(e)"]],
     Compound["for(p)",
              "Compartment"         -> "p",
              "Name"                -> "Formate (p)",
              "Formula"             -> "CH1O2",
              "Charge"              -> -1,
              "Capacity"            -> Cap["for(p)"],
              "Scale"               -> 1 *10^(-3) Molar["for(p)"]  ,
              "Potential"           -> P["for(p)_IC", "Value"->0.34048873604709042071*^-00003] Molar["for(p)"]],
     Compound["fum",
              "Compartment"         -> "c",
              "Name"                -> "Fumarate",
              "Formula"             -> "C4H2O4",
              "Charge"              -> -2,
              "Capacity"            -> Cap["fum"],
              "Potential"           -> P["fum_IC", "Value"->0.12873477029466405042*^-00003] Molar["fum"],
              "Scale"               -> 1 *10^(-3) Molar["fum"]  ],
     Compound["g3p",
              "Compartment"         -> "c",
              "Name"                -> "Glyceraldehyde 3-phosphate",
              "Formula"             -> "C3H5O6P",
              "Charge"              -> -2,
              "Capacity"            -> Cap["g3p"],
              "Potential"           -> P["g3p_IC", "Value"->0.71671159898047073856*^-00005] Molar["g3p"],
              "Scale"               -> 0.04 *10^(-3) Molar["g3p"]],
     Compound["g6p",
              "Compartment"         -> "c",
              "Name"                -> "D-Glucose 6-phosphate",
              "Formula"             -> "C6H11O9P",
              "Charge"              -> -2,
              "Capacity"            -> Cap["g6p"],
```

```
          "Potential"           -> P["g6p_IC", "Value"->0.55693784632831613165*^-00005] Molar["g6p"],
          "Scale"               -> 0.4 *10^(-3) Molar["g6p"]],
Compound["glc-D(e)",
          "Compartment"         -> "e",
          "Name"                -> "D-Glucose (e)",
          "Formula"             -> "C6H12O6",
          "Charge"              -> 0,
          "ReductionPriority"   -> 100,
          "Capacity"            -> Cap["glc-D(e)"],
          "Potential"           -> P["glc-D(e)_IC", "Value"->0.29844620512880920583*^-00005] Molar["glc-D(e)"],
          "Scale"               -> 10 *10^(-3) Molar["glc-D(e)"]],
Compound["glc-D(p)",
          "Compartment"         -> "p",
          "Name"                -> "D-Glucose (p)",
          "Formula"             -> "C6H12O6",
          "Charge"              -> 0,
          "Capacity"            -> Cap["glc-D(p)"],
          "Potential"           -> P["glc-D(p)_IC", "Value"->0.29844620512880840114*^-00005] Molar["glc-D(p)"],
          "Scale"               -> 10 *10^(-3) Molar["glc-D(p)"]],
Compound["glc-D",
          "Compartment"         -> "c",
          "Name"                -> "D-Glucose (c)",
          "Formula"             -> "C6H12O6",
          "Charge"              -> 0,
          "Capacity"            -> Cap["glc-D"],
          "Potential"           -> P["glc-D_IC", "Value"->0.31210766336169054892*^-00010] Molar["glc-D"],
          "Scale"               -> 10 *10^(-3) Molar["glc-D"]],
Compound["h",
          "Compartment"         -> "c",
          "Name"                -> "H+",
          "Formula"             -> "H",
          "Charge"              -> 1,
          "Capacity"            -> 10^(-pH),
          "Scale"               -> 1,
          "Potential"           -> 1,
          "Clamped"             -> True],
Compound["h(e)",
          "Compartment"         -> "e",
          "Name"                -> "H+ (e)",
          "Formula"             -> "H",
          "Charge"              -> 1,
          "Capacity"            -> 10^(-pH),
          "Clamped"             -> True,
          "Potential"           -> 1,
          "Scale"               -> 1],
Compound["h(p)",
          "Compartment"         -> "p",
          "Name"                -> "H+ (p)",
          "Formula"             -> "H",
          "Charge"              -> 1,
          "Capacity"            -> 10^(-pH),
          "Clamped"             -> True,
          "Potential"           -> 1,
          "Scale"               -> 1],
Compound["h(p+)",
          "Compartment"         -> "p+",
          "Name"                -> "H+ (p+)",
          "Formula"             -> "H",
          "Charge"              -> 1,
          "Capacity"            -> 10^(-pH),
          "Potential"           -> P["h(p+)_IC", "Value"->0.25399082023307277200*^-00007],
          "Scale"               -> 80],
Compound["h2o",
```

```
                  "Compartment"          -> "c",
                  "Name"                 -> "H2O",
                  "Formula"              -> "H2O",
                  "Charge"               -> 0,
                  "Capacity"             -> 1000/18.0153,
                  "Potential"            -> 1,
                  "Scale"                -> 1,
                  "Clamped"              -> True],
         Compound["h2o(e)",
                  "Compartment"          -> "e",
                  "Name"                 -> "H2O (e)",
                  "Formula"              -> "H2O",
                  "Charge"               -> 0,
                  "Capacity"             -> 1000/18.0153,
                  "Clamped"              -> True,
                  "Potential"            -> 1,
                  "Scale"                -> 1],
         Compound["h2o(p)",
                  "Compartment"          -> "p",
                  "Name"                 -> "H2O (p)",
                  "Formula"              -> "H2O",
                  "Charge"               -> 0,
                  "Capacity"             -> 1000/18.0153,
                  "Clamped"              -> True,
                  "Potential"            -> 1,
                  "Scale"                -> 1],
         Compound["h2",
                  "Compartment"          -> "c",
                  "Name"                 -> "Hydrogen",
                  "Formula"              -> "H2",
                  "Charge"               -> 0,
                  "Capacity"             -> Cap["h2"],
                  "Scale"                -> 1 *10^(-3) Molar["h2"]  ,
                  "Potential"            -> P["h2_IC", "Value"->0.10541389274229731684*^-00005] Molar["h2"]],
         Compound["h2(e)",
                  "Compartment"          -> "e",
                  "Name"                 -> "Hydrogen(e)",
                  "Formula"              -> "H2",
                  "Charge"               -> 0,
                  "Capacity"             -> Cap["h2(e)"],
                  "ReductionPriority"    -> 100,
                  "Scale"                -> 0.1 *10^(-3) Molar["h2(e)"]  ,
                  "Potential"            -> P["h2(e)_IC", "Value"->0.10541389274229750742*^-00005] Molar["h2(e)"]],
         Compound["h2(p)",
                  "Compartment"          -> "p",
                  "Name"                 -> "Hydrogen(p)",
                  "Formula"              -> "H2",
                  "Charge"               -> 0,
                  "Capacity"             -> Cap["h2(p)"],
                  "Scale"                -> 0.001 *10^(-3) Molar["h2(p)"]  ,
                  "Potential"            -> P["h2(p)_IC", "Value"->0.10541389274229742272*^-00005] Molar["h2(p)"]],
         Compound["glx",
                  "Compartment"          -> "c",
                  "Name"                 -> "glyoxylate",
                  "Formula"              -> "C2H1O3",
                  "Charge"               -> -1,
                  "Capacity"             -> Cap["glx"],
                  "Potential"            -> P["glx_IC", "Value"->0.43703485154296773470*^-00004] Molar["glx"],
                  "Scale"                -> 5 *10^(-3) Molar["glx"]],
         Compound["lac",
                  "Compartment"          -> "c",
                  "Name"                 -> "D-Lactate",
                  "Formula"              -> "C3H5O3",
```

```
          "Charge"              -> -1,
          "Capacity"            -> Cap["lac"],
          "Scale"               -> 1 *10^(-3) Molar["lac"]  ,
          "Potential"           -> P["lac_IC", "Value"->0.29711255298134422644*^-00011] Molar["lac"]],
Compound["lac(e)",
          "Compartment"         -> "e",
          "Name"                -> "D-Lactate (e)",
          "Formula"             -> "C3H5O3",
          "Charge"              -> -1,
          "Capacity"            -> Cap["lac(e)"],
          "ReductionPriority"   -> 100,
          "Scale"               -> 0.1 *10^(-3) Molar["lac(e)"]  ,
          "Potential"           -> P["lac(e)_IC", "Value"->0.15538476402519336819*^-00013] Molar["lac(e)"]],
Compound["lac(p)",
          "Compartment"         -> "p",
          "Name"                -> "D-Lactate (p)",
          "Formula"             -> "C3H5O3",
          "Charge"              -> -1,
          "Capacity"            -> Cap["lac(p)"],
          "Scale"               -> 0.001 *10^(-3) Molar["lac(p)"]  ,
          "Potential"           -> P["lac(p)_IC", "Value"->0.15538476402519292643*^-00013] Molar["lac(p)"]],
Compound["mal",
          "Compartment"         -> "c",
          "Name"                -> "L-Malate",
          "Formula"             -> "C4H4O5",
          "Charge"              -> -2,
          "Capacity"            -> Cap["mal"],
          "Potential"           -> P["mal_IC", "Value"->0.52055632663692533342*^-00003] Molar["mal"],
          "Scale"               -> 5 *10^(-3) Molar["mal"]  ],
Compound["mqn8",
          "Compartment"         -> "cm",
          "Name"                -> "Menaquinone 8",
          "Formula"             -> "C51H72O2",
          "Charge"              -> 0,
          "Charge"              -> 0,
          "Capacity"            -> Cap["mqn8"],
          "Potential"           -> P["mqn8_IC", "Value"->0.62283403153823128917*^-00003] Molar["mqn8"],
          "Scale"               -> 1.9 *10^(-3) Molar["mqn8"]],
Compound["mql8",
          "Compartment"         -> "cm",
          "Name"                -> "Menaquinol 8",
          "Formula"             -> "C51H74O2",
          "Charge"              -> 0,
          "Capacity"            -> Cap["mql8"],
          "Potential"           -> P["mql8_IC", "Value" ->0.21681237817381661508*^-00005] Molar["mql8"],
          "Scale"               -> 1 *10^(-3) Molar["mql8"]],
Flux["mqn8syn",
      "Reactants"               -> 0,
      "Products"                -> "mqn8",
      "Compartment"             -> "cm",
      "Name"                    -> "de novo synthesis of mqn8",
      "Clamped"                 -> True,
      "Comment"                 -> "de novo synthesis of mqn8 decreases linearily with aerobiosis",
      "Flux"                    -> P["mqn8syn_k","Value"-> 0.5*^-4]*(P["mqn8syn_k0","Value"-> 0.1]+(1-P["mqn8syn_k0"])*(1-TH[c["a"][t],0,1]))],
Compound["nad",
          "Compartment"         -> "c",
          "Name"                -> "Nicotinamide adenine dinucleotide",
          "Formula"             -> "C21H26N7O14P2",
          "Charge"              -> -1,
          "Capacity"            -> Cap["nad"],
          "Potential"           -> P["nad_IC", "Value"->0.15674537113365594361*^-00002]Molar["nad"],
          "Scale"               -> 0.5  *10^(-3) Molar["nad"],
          "ReductionPriority"   -> 100],
```

```
Compound["nadh",
         "Compartment"         -> "c",
         "Name"                -> "Nicotinamide adenine dinucleotide - reduced",
         "Formula"             -> "C21H27N7O14P2",
         "Charge"              -> -2,
         "Capacity"            -> Cap["nadh"],
         "Potential"           -> P["nadh_IC", "Value"->0.11736010442374816240*^-00005] Molar["nadh"],
         "Scale"               -> 0.5  *10^(-3) Molar["nadh"],
         "ReductionPriority"   -> 100],
Flux["nadsyn",
     "Reactants"               -> -"+",
     "Products"                -> "nad",
     "Compartment"             -> "c",
     "Name"                    -> "de novo synthesis of nad",
     "Comment"                 -> "de novo synthesis of nad is adjusted such that the total concentration nadh+nad is approx. constant",
     "Clamped"                 -> True,
     "Flux"                    -> -P["nadsyn_vmax","Value"-> 10]*(c["nadh"][t]+c["nad"][t]-10^(-3)*P["nad_tot","Value"-> 1.6])],
Compound["nadp",
         "Compartment"         -> "c",
         "Name"                -> "Nicotinamide dinucleotide phosphate",
         "Formula"             -> "C21H25N7O17P3",
         "Charge"              -> -3,
         "Capacity"            -> Cap["nadp"],
         "Potential"           -> P["nadp_IC", "Value"->0.72345028962637084003*^-00004] Molar["nadp"],
         "Scale"               -> 0.5  *10^(-3) Molar["nadp"]
        ],
Compound["nadph",
         "Compartment"         -> "c",
         "Name"                -> "Nicotinamide adenine dinucleotide phosphate - reduced",
         "Formula"             -> "C21H26N7O17P3",
         "Charge"              -> -4,
         "Capacity"            -> Cap["nadph"],
         "Potential"           -> P["nadph_IC", "Value"->0.90804704146177351014*^-00003] Molar["nadph"],
         "Scale"               -> 0.5  *10^(-3) Molar["nadph"]
        ],
Flux["nadpsyn",
     "Reactants"              -> -3 "+",
     "Products"               -> "nadp",
     "Compartment"            -> "c",
     "Name"                   -> "de novo synthesis of nadp",
     "Comment"                -> "de novo synthesis of nadp is adjusted such that the total concentration nadp+nadph is approx. constant",
     "Clamped"                -> True,
     "Flux"                   -> -P["nadpsyn_vmax","Value"-> 10]*(c["nadph"][t]+c["nadp"][t]-10^(-3)*P["nadp_tot","Value"-> 1])],
Compound["o2",
         "Compartment"         -> "c",
         "Name"                -> "O2",
         "Formula"             -> "O2",
         "Charge"              -> 0,
         "Capacity"            -> Cap["o2"],
         "Potential"           -> P["o2_IC", "Value"->0.28559219566216671152*^-00004] Molar["o2"],
         "Scale"               -> 0.001  *10^(-3) Molar["o2"]],
Compound["o2(e)",
         "Compartment"         -> "e",
         "Name"                -> "O2 (e)",
         "Formula"             -> "O2",
         "Charge"              -> 0,
         "ReductionPriority"   -> 100,
         "Capacity"            -> Cap["o2(e)"],
         "Potential"           -> P["o2(e)_IC", "Value"->0.28559219566216671152*^-00004] Molar["o2(e)"],
         "Scale"               -> 0.001  *10^(-3) Molar["o2(e)"]],
Compound["o2(p)",
         "Compartment"         -> "p",
         "Name"                -> "O2 (p)",
```

```
          "Formula"            -> "O2",
          "Charge"             -> 0,
          "Capacity"           -> Cap["o2(p)"],
          "Potential"          -> P["o2(p)_IC", "Value"->0.28559219566216671152*^-00004] Molar["o2(p)"],
          "Scale"              -> 0.001  *10^(-3) Molar["o2(p)"]],
Compound["icit",
          "Compartment"        -> "c",
          "Name"               -> "Isocitrate",
          "Formula"            -> "C6H5O7",
          "Charge"             -> -3,
          "Capacity"           -> Cap["icit"],
          "Scale"              -> 0.0001  *10^(-3) Molar["icit"]  ,
          "Potential"          -> P["icit_IC", "Value"->0.10246456976427944174*^-00008] Molar["icit"]],
Compound["oaa",
          "Compartment"        -> "c",
          "Name"               -> "Oxaloacetate",
          "Formula"            -> "C4H2O5",
          "Charge"             -> -2,
          "Capacity"           -> Cap["oaa"],
          "Potential"          -> P["oaa_IC", "Value"->0.30751954567482429014*^-00004] Molar["oaa"],
          "Scale"              -> 0.05  *10^(-3) Molar["oaa"]],
Compound["pep",
          "Compartment"        -> "c",
          "Name"               -> "Phosphoenolpyruvate",
          "Formula"            -> "C3H2O6P",
          "Capacity"           -> Cap["pep"],
          "ReductionPriority"  -> 100,
          "Charge"             -> -3,
          "Potential"          -> P["pep_IC", "Value"->0.43808611217223609232*^-00003] Molar["pep"],
          "Scale"              -> 1.8  *10^(-3) Molar["pep"]],
Compound["pyr",
          "Compartment"        -> "c",
          "Name"               -> "Pyruvate",
          "Formula"            -> "C3H3O3",
          "Charge"             -> -1,
          "Capacity"           -> Cap["pyr"],
          "Potential"          -> P["pyr_IC", "Value"->0.24547273017158144295*^-00006] Molar["pyr"],
          "Scale"              -> 1.9  *10^(-3) Molar["pyr"]],
Compound["pi",
          "Compartment"        -> "c",
          "Name"               -> "Phosphate",
          "Formula"            -> "HO4P",
          "Charge"             -> -2,
          "Capacity"           -> Cap["pi"],
          "Potential"          -> P["pi_IC", "Value"->0.10000000000000008882*^-00001] Molar["pi"],
          "Scale"              -> 10  *10^(-3) Molar["pi"]  ,
          "Clamped"            -> True],
Compound["ppi",
          "Compartment"        -> "c",
          "Name"               -> "diphosphate",
          "Formula"            -> "HO7P2",
          "Charge"             -> -3,
          "Capacity"           -> Cap["ppi"],
          "Potential"          -> P["ppi_IC", "Value"->0.66569856464137874502*^-00008] Molar["pi"],
          "Scale"              -> 10  *10^(-3) Molar["pi"],
          "ReductionPriority"  -> 100],
Compound["q8*",
          "Compartment"        -> "cm",
          "Name"               -> "Ubiquinone-8 (active and inactive)",
          "Comment"            -> ("In order to reproduce the observation that even in the complete anaerobic" <>
                                   "case a substantial part of the quinone pool is oxidized, we need to" <>
                                   "introduce a constant pool of oxidized quinones that does not participate"<>
                                   "in any reaction." <>
```

```
                                  "Concentration is calculated as the sum of the active and an assumed inactive form"<>
                                  "with constant concentration. The inactive form does not participate in any reactions."),
         "Formula"              -> "C49H74O4",
         "Charge"               -> 0,
         "Capacity"             -> 1,
         "Potential"            -> c["q8"][t]+P["q8-inactive","Value"-> 0.03*^-2],
         "Scale"                -> 0.5  *10^(-3),
         "Clamped"              -> True,
         "ReductionPriority"    -> 100],
Compound["q8h2*",
         "Compartment"          -> "cm",
         "Name"                 -> "Ubiquinol-8 (active and inactive)",
         "Comment"              -> "Concentration is calculated as the sum of the active and an assumed inactive form.",
         "Formula"              -> "C49H74O4",
         "Charge"               -> 0,
         "Capacity"             -> 1,
         "Potential"            -> c["q8h2"][t]+P["q8h2-inactive","Value"->0],
         "Scale"                -> 0.5  *10^(-3),
         "Clamped"              -> True,
         "ReductionPriority"    -> 100],
Compound["q8",
         "Compartment"          -> "cm",
         "Name"                 -> "Ubiquinone-8",
         "Formula"              -> "C49H74O4",
         "Charge"               -> 0,
         "Capacity"             -> Cap["q8"],
         "Potential"            -> P["q8_IC", "Value"->0.23429890437561234733*^-00002] Molar["q8"],
         "Scale"                -> 0.5  *10^(-3) Molar["q8"],
         "ReductionPriority"    -> 100],
Compound["q8h2",
         "Compartment"          -> "cm",
         "Name"                 -> "Ubiquinol-8",
         "Formula"              -> "C49H76O4",
         "Charge"               -> 0,
         "Capacity"             -> Cap["q8h2"],
         "Potential"            -> P["q8h2_IC", "Value"->0.30508716345764719252*^-00004] Molar["q8h2"],
         "Scale"                -> 0.5  *10^(-3) Molar["q8h2"],
         "ReductionPriority"    -> 100],
Flux["q8syn",
     "Reactants"                -> 0,
     "Products"                 -> "q8h2",
     "Compartment"              -> "cm",
     "Name"                     -> "de novo synthesis of q8",
     "Comment"                  -> "de novo synthesis of q8 inreases linearily with aerobiosis",
     "Clamped"                  -> True,
     "Flux"                     -> P["q8syn_k","Value"-> 2.0*^-4]*(P["q8syn_k0","Value"-> 0.1]+(1-P["q8syn_k0"])*TH[c["a"][t],0,1])],
Compound["r5p",
         "Compartment"          -> "c",
         "Name"                 -> "alpha-D-Ribose 5-phosphate",
         "Formula"              -> "C5H9O8P",
         "Charge"               -> -2,
         "Capacity"             -> Cap["r5p"],
         "Potential"            -> P["r5p_IC", "Value"->0.14331233272875701831*^-00006] Molar["r5p"],
         "Scale"                -> 0.05  *10^(-3) Molar["r5p"]  ],
Compound["ru5p-D",
         "Compartment"          -> "c",
         "Name"                 -> "D-Ribulose 5-phosphate",
         "Formula"              -> "C5H9O8P",
         "Charge"               -> -2,
         "Capacity"             -> Cap["ru5p-D"],
         "Potential"            -> P["ru5p-D_IC", "Value"->0.11989834429487965431*^-00006] Molar["ru5p-D"],
         "Scale"                -> 1  *10^(-3) Molar["ru5p-D"]],
Compound["s7p",
```

```
          "Compartment"         -> "c",
          "Name"                -> "Sedoheptulose 7-phosphate",
          "Formula"             -> "C7H13O10P",
          "Charge"              -> -2,
          "Capacity"            -> Cap["s7p"],
          "Potential"           -> P["s7p_IC", "Value"->0.10004005624979777423*^-00007] Molar["s7p"],
          "Scale"               -> 1  *10^(-3) Molar["s7p"]],
Compound["succ",
          "Compartment"         -> "c",
          "Name"                -> "Succinate",
          "Formula"             -> "C4H4O4",
          "Charge"              -> -2,
          "Capacity"            -> Cap["succ"],
          "Potential"           -> P["succ_IC", "Value"->0.25257814906474049076*^-00004] Molar["succ"],
          "Scale"               -> 1  *10^(-3) Molar["succ"]],
Compound["succ(e)",
          "Compartment"         -> "e",
          "Name"                -> "Succinate (e)",
          "Formula"             -> "C4H4O4",
          "Charge"              -> -2,
          "ReductionPriority"   -> 100,
          "Capacity"            -> Cap["succ(e)"],
          "Potential"           -> P["succ(e)_IC", "Value"->0.10352347695864204716*^-00005] Molar["succ(e)"],
          "Scale"               -> 1  *10^(-3) Molar["succ(e)"]  ],
Compound["succ(p)",
          "Compartment"         -> "p",
          "Name"                -> "Succinate (periplasm)",
          "Formula"             -> "C4H4O4",
          "Charge"              -> -2,
          "Capacity"            -> Cap["succ(p)"],
          "Potential"           -> P["succ(p)_IC", "Value"->0.10352347695864196246*^-00005] Molar["succ(p)"],
          "Scale"               -> 1  *10^(-3) Molar["succ(p)"]  ],
Compound["succoa",
          "Compartment"         -> "c",
          "Name"                -> "Succinyl-CoA",
          "Formula"             -> "C25H35N7O19P3S",
          "Charge"              -> -5,
          "Capacity"            -> Cap["succoa"],
          "ReductionPriority"   -> 100,
          "Potential"           -> P["succoa_IC", "Value"->0.12183237985245739214*^-00004] Molar["succoa"],
          "Scale"               -> 0.5  *10^(-3) Molar["succoa"]],
Compound["xu5p-D",
          "Compartment"         -> "c",
          "Name"                -> "D-Xylulose 5-phosphate",
          "Formula"             -> "C5H9O8P",
          "Charge"              -> -2,
          "Capacity"            -> Cap["xu5p-D"],
          "Potential"           -> P["xu5p-D_IC", "Value"->0.22282795379954100447*^-00006] Molar["xu5p-D"],
          "Scale"               -> 1  *10^(-3) Molar["xu5p-D"]],

(* *** In and out-fluxes to and from the reactor          *** *)
Flux["GLC:in",
     "Reactants"                -> 0,
     "Products"                 -> "glc-D(e)",
     "Name"                     -> "glucose in/out",
     "Subsystem"                -> "in- and outflow of glucose into/out of the reactor",
     "Comment"                  -> "This flux is given by the chemostat equation",
     "Compartment"              -> "e",
     "Flux"                     -> P["DIL"]*P["GLCin","Value"->20*10^(-3)],
     "Clamped"                  -> True],
Compound["DO2sat",
          "Name"                -> "oxygen tension in saturated water",
          "TeXExport"           -> False,
```

```
          "Link"                 -> {"DOI"->  "http://dx.doi.org/10.1016/0011-7471(70)90037-9"},
          "Comment"              -> ("This concentration is computed as described in"<>
                                     "''The solubility of nitrogen, oxygen and argon in water and seawater''"<>
                                     "Weiss, R.F. (1970) Deep Sea Research and Oceanographic Abstracts."<>
                                     "vol. 17 (4) p. 721-735."),
          "Clamped"              -> True,
          "Compartment"          -> "e",
          "Capacity"             -> 1,
          "Potential"            -> Module[{A1 = -173.4292, A2 = 249.6339, A3 = 143.3483, A4 = -21.8492,
                                            B1 = -0.033096, B2 = 0.014259, B3 = -0.001700},
                                           1/1000 *    (* mM/L -> M/L*)
                                           1/P["MolarMassO2"] *     (* mg/L -> mM/L*)
                                           1.4276 *    (* ml/L -> mg/L*)
                                           p/101325 * (* p0   -> p   *)
                                           Exp[A1 + A2*100/TEMP + A3*Log[TEMP/100] + A4*TEMP/100 +
                                               P["Salinity"]*(B1 + B2*TEMP/100 + B3*(TEMP/100)^2)]]],
Flux["O2:in",
     "Reactants"                 -> 0,
     "Products"                  -> "o2(e)",
     "Name"                      -> "oxygen in/out",
     "Subsystem"                 -> "in- and outflow of oxygen into/out of the reactor",
     "Compartment"               -> "e",
     "Flux"                      -> P["amax", "Value"->45]*c["DO2sat"][t]*c["a"][t]-P["o2_out","Value"->100]*c["o2(e)"][t],
     "Clamped"                   -> True],
Flux["CO2:in",
     "Reactants"                 -> 0,
     "Products"                  -> "co2(e)",
     "Name"                      -> "co2 in",
     "Subsystem"                 -> "in and outflow of reactor",
     "Compartment"               -> "e",
     "Flux"                      -> P["co2_in","Value"->1*^-1]-P["co2_out","Value"-> 0.5*^2] c["co2(e)"][t],
     "Clamped"                   -> True],
Flux["H2:in",
     "Reactants"                 -> 0,
     "Products"                  -> "h2(e)",
     "Name"                      -> "h2 in",
     "Subsystem"                 -> "in and outflow of reactor",
     "Compartment"               -> "e",
     "Flux"                      -> P["h2_in","Value"->0]-P["h2_out","Value"-> 10.0*^2] c["h2(e)"][t],
     "Clamped"                   -> True],
Compound["a",
          "Name"                 -> "aerobiosis",
          "Comment"              -> "This is an auxiliary variable modeling aerobiosis.",
          "TeXExport"            -> False,
          "Clamped"              -> True,
          "Compartment"          -> "e",
          "Capacity"             -> 1,
          "Potential"            ->
          P["a_x_0","Value"->1.6]+
          (P["a_x_1","Value"->1.6]-P["a_x_0"])*TH[t,0          ,P["a_t_1","Value"->t0]]+
          (P["a_x_2","Value"->0.0]-P["a_x_1"])*TH[t,P["a_t_1"],P["a_t_2","Value"->t1]]+
          (P["a_x_3","Value"->1.6]-P["a_x_2"])*TH[t,P["a_t_2"],P["a_t_3","Value"->t2]]],

(* *** Transport across periplasmatic membrane            *** *)
Flux["AC:e->p",
     "Reactants"                 -> "ac(e)",
     "Products"                  -> "ac(p)",
     "Name"                      -> "acetate transport through periplasmatic membrane",
     "Subsystem"                 -> "Transport, Extracellular",
     "Compartment"               -> "om",
     "Resistance"                -> 0],
Flux["CO2:e->p",
     "Reactants"                 -> "co2(e)",
```

```
     "Products"                -> "co2(p)",
     "Name"                    -> "co2 transport through periplasmatic membrane",
     "Subsystem"               -> "Transport, Extracellular",
     "Compartment"             -> "om",
     "Resistance"              -> 0],
Flux["H2:e->p",
     "Reactants"               -> "h2(e)",
     "Products"                -> "h2(p)",
     "Name"                    -> "h2 transport through periplasmatic membrane",
     "Subsystem"               -> "Transport, Extracellular",
     "Compartment"             -> "om",
     "Resistance"              -> 0],
Flux["ETOH:e->p",
     "Reactants"               -> "etoh(e)",
     "Products"                -> "etoh(p)",
     "Name"                    -> "ethanol transport through periplasmatic membrane",
     "Subsystem"               -> "Transport, Extracellular",
     "Compartment"             -> "om",
     "Resistance"              -> 0],
Flux["FOR:e->p",
     "Reactants"               -> "for(e)",
     "Products"                -> "for(p)",
     "Name"                    -> "formate transport through periplasmatic membrane",
     "Subsystem"               -> "Transport, Extracellular",
     "Compartment"             -> "om",
     "Resistance"              -> 0],
Flux["LAC:e->p",
     "Reactants"               -> "lac(e)",
     "Products"                -> "lac(p)",
     "Name"                    -> "lactate transport through periplasmatic membrane",
     "Subsystem"               -> "Transport, Extracellular",
     "Compartment"             -> "om",
     "Resistance"              -> 0],
Flux["GLC:e->p",
     "Reactants"               -> "glc-D(e)",
     "Products"                -> "glc-D(p)",
     "Name"                    -> "glucose transport through periplasmatic membrane",
     "Subsystem"               -> "Transport, Extracellular",
     "Compartment"             -> "om",
     "Resistance"              -> 0],
Flux["O2:e->p",
     "Reactants"               -> "o2(e)",
     "Products"                -> "o2(p)",
     "Name"                    -> "oxygen transport through periplasmatic membrane",
     "Subsystem"               -> "Transport, Extracellular",
     "Compartment"             -> "om",
     "Resistance"              -> 0],
Flux["SUCC:e->p",
     "Reactants"               -> "succ(e)",
     "Products"                -> "succ(p)",
     "Name"                    -> "succinate transport through periplasmatic membrane",
     "Subsystem"               -> "Transport, Extracellular",
     "Compartment"             -> "om",
     "Resistance"              -> 0],

(* *** Transport across cytoplasmatic membrane            *** *)
(* organic compounds *)
Flux["GLCpts",
     "Reactants"               -> "pep" + "glc-D(p)",
     "Products"                -> "pyr" + "g6p",
     "Name"                    -> "glucose transport via PEP:Pyr PTS",
     "Subsystem"               -> "Transport, Extracellular",
     "Compartment"             -> "cm",
```

```
      "Link"                 -> {"EcoCyc" -> "http://biocyc.org/ECOLI/NEW-IMAGE?type=NIL&object=TRANS-RXN-157"},
      "Resistance"           -> P["GLCpts_k","Value"-> 1*^-6]/(Cap["h2o"]*Cap["pep"]*Cap["glc-D(p)"])/c["E-GLCpts"][t]
     ],
Compound["E-GLCpts",
         "Compartment"       -> "cm",
         "Name"              -> "enzyme of GLCpts",
         "Capacity"          -> 1,
         "Link"              -> {"EcoCyc" -> "http://biocyc.org/ECOLI/NEW-IMAGE?type=ENZYME&object=CPLX-157"},
         "Comment"           -> {"We assume that all PTS-Transport glucose occurs via the glucose PTS and none via the mannose PTS."},
         "Potential"         -> P["E-GLCpts_IC", "Value"->0.29321788673989548357*^+00001],
         "Scale"             -> 1],
Flux["E-GLCpts-syn",
      "Reactants"            -> 0 ,
      "Products"             -> "E-GLCpts",
      "Name"                 -> "enzyme synthesis",
      "Compartment"          -> "c",
      "Flux"                 -> s[P["GLCpts_CRP","Value"->   1.0],c["CRP"][t]]*s[P["GLCpts_FruR","Value"->  1.0],1-c["FruR"][t]],
      "Clamped"              -> True],

Flux["GLCabc",
      "Reactants"            -> "h2o" + "atp" + "glc-D(p)",
      "Products"             -> "adp" + "glc-D" + "h" + "pi",
      "Name"                 -> "glucose transport via ABC (mgl)",
      "Subsystem"            -> "Transport, Extracellular",
      "Compartment"          -> "cm",
      "EC"                   -> "3.6.3.17",
      "Link"                 -> {"EcoCyc" -> "http://biocyc.org/ECOLI/NEW-IMAGE?type=REACTION&object=ABC-18-RXN",
                                 "PMID"-> "8310178", "PMID"-> "8703508", "PMID"-> "15066832", "PMID"-> "22923596"},
      "Resistance"           -> P["GLCabc_k","Value"-> 1*^-4]/(Cap["h2o"]*Cap["atp"]*Cap["glc-D(p)"])/c["E-GLCabc"][t]
     ],
Compound["E-GLCabc",
         "Compartment"       -> "cm",
         "Name"              -> "enzyme of GLCabc",
         "Capacity"          -> 1,
         "Formula"           -> "R3",
         "Potential"         -> P["E-GLCabc_IC", "Value"->0.27903721897532660634*^+00001],
         "Link"              -> {"EcoCyc" -> "http://biocyc.org/ECOLI/NEW-IMAGE?type=ENZYME&object=ABC-18-CPLX"},
         "Comment"           -> ("Because in the microarray data mglAC and mglB are differently expressed and"<>
                                 "because EcoCyc lists an promoter between mglB and mglAC, we distinguish between"<>
                                 "these two genes here."),
         "Scale"             -> 1],
Compound["E-GLCabc-mglB",
         "Compartment"       -> "cm",
         "Name"              -> "enzyme of GLCabc (MglB only)",
         "Capacity"          -> 0.01,
         "Formula"           -> "R1",
         "Potential"         -> P["E-GLCabc-mglB_IC", "Value"->0.27905902278788969895*^+00001],
         "Link"              -> {"EcoCyc" -> "http://biocyc.org/ECOLI/NEW-IMAGE?type=ENZYME&object=MGLB-MONOMER"},
         "Scale"             -> 1],
Flux["E-GLCabc-mglB-syn",
      "Reactants"            -> 0 ,
      "Products"             -> "E-GLCabc-mglB",
      "Name"                 -> "enzyme synthesis",
      "Compartment"          -> "c",
      "Flux"                 -> s[P["GLCabc-mglB_CRP","Value"-> 1],c["CRP"][t]],
      "Clamped"              -> True],
Compound["E-GLCabc-mglAC",
         "Compartment"       -> "cm",
         "Name"              -> "enzyme of GLCabc (MglAC only)",
         "Capacity"          -> 0.01,
         "Formula"           -> "R1",
         "Potential"         -> P["E-GLCabc-mglAC_IC", "Value"->0.99996093256856169451*^-00003],
         "Link"              -> {"EcoCyc" -> "http://biocyc.org/ECOLI/NEW-IMAGE?type=GENE&object=EG10592",
```

```
                                  "EcoCyc" -> "http://biocyc.org/ECOLI/NEW-IMAGE?type=ENZYME&object=MGLC-MONOMER"},
         "Scale"                 -> 1],
Flux["E-GLCabc-mglAC-syn",
     "Reactants"                 -> 0 ,
     "Products"                  -> "E-GLCabc-mglAC",
     "Name"                      -> "enzyme synthesis",
     "Compartment"               -> "c",
     "Flux"                      -> s[P["GLCabc-mglAC_CRP","Value"-> 1],c["CRP"][t]],
     "Clamped"                   -> True],
Flux["E-GLCabc-syn",
     "Reactants"                 -> 2 "E-GLCabc-mglAC" + "E-GLCabc-mglB" ,
     "Products"                  -> "E-GLCabc",
     "Name"                      -> "formation of E-GLCabc",
     "Subsystem"                 -> "Transport, Extracellular",
     "Compartment"               -> "cm",
     "Link"                      -> {"EcoCyc" -> "http://biocyc.org/ECOLI/NEW-IMAGE?type=REACTION&object=ABC-18-RXN"},
     "Resistance"                -> 0
    ],
Flux["HEX1",
     "Reactants"                 -> "atp" + "glc-D",
     "Products"                  -> "adp" + "g6p" + "h",
     "Name"                      -> "hexokinase (D-glucose:ATP)",
     "Subsystem"                 -> "Glycolysis/Gluconeogenesis",
     "Compartment"               -> "c",
     "EC"                        -> "2.7.1.1",
     "Link"                      -> {"EcoCyc" -> "http://biocyc.org/ECOLI/NEW-IMAGE?type=REACTION&object=GLUCOKIN-RXN"},
     "Resistance"                -> 0
    ],
Compound["E-HEX1",
         "Compartment"           -> "c",
         "Name"                  -> "enzyme of HEX1",
         "Capacity"              -> 1,
         "Potential"             -> P["E-HEX1_IC", "Value"->0.14398625135581835099*^+00001],
         "Link"                  -> {"EcoCyc" -> "http://biocyc.org/ECOLI/NEW-IMAGE?type=ENZYME&object=GLUCOKIN-MONOMER"},
         "Scale"                 -> 1],
Flux["E-HEX1-syn",
     "Reactants"                 -> 0 ,
     "Products"                  -> "E-HEX1",
     "Name"                      -> "enzyme synthesis",
     "Compartment"               -> "c",
     "Flux"                      -> s[P["HEX1_FruR","Value"-> 4],1-c["FruR"][t]],
     "Clamped"                   -> True],
Flux["SUCCt",
     "Reactants"                 -> "succ" + P["SUCCt_h","Value"-> 0] "h(p+)",
     "Products"                  -> "succ(p)" + P["SUCCt_h"] "h" ,
     "Name"                      -> "succinate transport through cytoplasmatic membrane",
     "Subsystem"                 -> "Transport, Extracellular",
     "Compartment"               -> "cm",
     "Link"                      -> {"DOI" -> "http://dx.doi.org/10.1111/j.1432-1033.1994.tb18903.x"},
     "Resistance"                -> P["SUCCt_k","Value"-> 1.0]/(Cap["succ"]*Cap["h"]^1)/c["E-SUCCt"][t]],
Compound["E-SUCCt",
         "Compartment"           -> "cm",
         "Name"                  -> "enzyme of SUCCt",
         "Capacity"              -> 1,
         "Potential"             -> P["E-SUCCt_IC", "Value"->0.28455751392653877777*^-00002],
         "Scale"                 -> 1],
Flux["E-SUCCt-syn",
     "Reactants"                 -> 0 ,
     "Products"                  -> "E-SUCCt",
     "Name"                      -> "enzyme synthesis",
     "Compartment"               -> "c",
     "Flux"                      -> s[P["SUCCt_FNR","Value"-> 10],c["FNR"][t]],
     "Clamped"                   -> True],
```

```
Flux["ACt",
     "Reactants"              -> "ac(p)" + P["ACt_h","Value"-> 1] "h" ,
     "Products"               -> "ac" + P["ACt_h"] "h(p+)",
     "Name"                   -> "acetate transport through cytoplasmatic membrane",
     "Subsystem"              -> "Transport, Extracellular",
     "Compartment"            -> "cm",
     "Resistance"             -> 0],
Flux["ETOHt",
     "Reactants"              -> "etoh(p)"+ P["ETOHt_h","Value"-> 1] "h",
     "Products"               -> "etoh"+ P["ETOHt_h"] "h(p+)",
     "Name"                   -> "ethanol transport through cytoplasmatic membrane",
     "Subsystem"              -> "Transport, Extracellular",
     "Compartment"            -> "cm",
     "Resistance"             -> 0],
Flux["FORt",
     "Reactants"              -> "for(p)" + P["FORt_h","Value"-> 1] "h",
     "Products"               -> "for" + P["FORt_h"] "h(p+)",
     "Name"                   -> "formate transport through cytoplasmatic membrane",
     "Subsystem"              -> "Transport, Extracellular",
     "Compartment"            -> "cm",
     "Resistance"             -> 0],
Flux["LACt",
     "Reactants"              -> "lac" + P["LACt_h","Value"-> 1] "h",
     "Products"               -> "lac(p)" + P["LACt_h"] "h(p+)",
     "Name"                   -> "D-lactate transport through cytoplasmatic membrane",
     "Subsystem"              -> "Transport, Extracellular",
     "Compartment"            -> "cm",
     "Resistance"             -> 0],

(* mineralic compounds *)
Flux["O2t",
     "Reactants"              -> "o2(p)",
     "Products"               -> "o2",
     "Name"                   -> "o2 transport through cytoplasmatic membrane",
     "Subsystem"              -> "Transport, Extracellular",
     "Compartment"            -> "cm",
     "Resistance"             -> 0],
Flux["CO2t",
     "Reactants"              -> "co2(p)",
     "Products"               -> "co2",
     "Name"                   -> "CO2 transport through cytoplasmatic membrane",
     "Subsystem"              -> "Transport, Extracellular",
     "Compartment"            -> "cm",
     "Resistance"             -> 0],
Flux["H2t",
     "Reactants"              -> "h2(p)",
     "Products"               -> "h2",
     "Name"                   -> "H2 transport through cytoplasmatic membrane",
     "Subsystem"              -> "Transport, Extracellular",
     "Compartment"            -> "cm",
     "Resistance"             -> 0],

(* *** glycolyis                                         *** *)
Flux["PGI",
     "Reactants"              -> "g6p",
     "Products"               -> "f6p",
     "Name"                   -> "glucose-6-phosphate isomerase",
     "Subsystem"              -> "Glycolysis/Gluconeogenesis",
     "EC"                     -> "5.3.1.9",
     "Compartment"            -> "c",
     "Link"                   -> {"EcoCyc" -> "http://biocyc.org/ECOLI/NEW-IMAGE?type=REACTION&object=PGLUCISOM-RXN"},
     "Resistance"             -> 0],
Compound["E-PGI",
```

```
            "Compartment"         -> "c",
            "Name"                -> "enzyme of PGI",
            "Capacity"            -> 1,
            "Potential"           -> P["E-PGI_IC", "Value"->0.49999774764485973577*^+00001],
            "Link"                -> {"EcoCyc"->"http://biocyc.org/ECOLI/NEW-IMAGE?type=GENE&object=EG10702"},
            "Scale"               -> 1],
Flux["E-PGI-syn",
        "Reactants"               -> 0 ,
        "Products"                -> "E-PGI",
        "Name"                    -> "enzyme synthesis",
        "Compartment"             -> "c",
        "Flux"                    -> 1,
        "Clamped"                 -> True],
Flux["PFK",
        "Reactants"               -> "atp" + "f6p",
        "Products"                -> "adp" + "fdp" + "h",
        "Name"                    -> "phosphofructokinase",
        "Subsystem"               -> "Glycolysis/Gluconeogenesis",
        "EC"                      -> "2.7.1.11",
        "Compartment"             -> "c",
        "Link"                    -> {"EcoCyc" -> "http://biocyc.org/ECOLI/NEW-IMAGE?type=REACTION&object=6PFRUCTPHOS-RXN"},
        "Resistance"              -> (P["PFK_k","Value"-> 1*^-15]/(Cap["atp"]*Cap["f6p"])/c["E-PFK"][t])*c["adp"][t]^P["PFK_n_adp","Value"-> -2]],
Compound["E-PFK",
            "Compartment"         -> "c",
            "Name"                -> "enzyme of PFK",
            "Capacity"            -> 1,
            "Potential"           -> P["E-PFK_IC", "Value"->0.14398625135581835099*^+00001],
            "Link"                -> {"EcoCyc"->{"http://biocyc.org/ECOLI/NEW-IMAGE?type=ENZYME&object=6PFK-1-CPX"}},
            "Comment"             -> {"According to EcoCyc, PFK1 has the main activity. Thus, we neglect PFK2"},
            "Scale"               -> 1],
Flux["E-PFK-syn",
        "Reactants"               -> 0 ,
        "Products"                -> "E-PFK",
        "Name"                    -> "enzyme synthesis",
        "Compartment"             -> "c",
        "Flux"                    -> s[P["PFK_FruR","Value"-> 2],1-c["FruR"][t]],
        "Clamped"                 -> True],
Flux["FBA",
        "Reactants"               -> "fdp",
        "Products"                -> "dhap" + "g3p",
        "Name"                    -> "fructose-bisphosphate aldolase",
        "Subsystem"               -> "Glycolysis/Gluconeogenesis",
        "EC"                      -> "4.1.2.13",
        "Compartment"             -> "c",
        "Link"                    -> {"EcoCyc"->"http://biocyc.org/ECOLI/NEW-IMAGE?type=REACTION&object=F16ALDOLASE-RXN"},
        "Resistance"              -> 0/c["E-FBA"][t]],
Compound["E-FBA",
            "Compartment"         -> "c",
            "Name"                -> "enzyme of FBA",
            "Capacity"            -> 1,
            "Potential"           -> P["E-FBA_IC", "Value"->0.14398084985807837466*^+00001],
            "Link"                -> {"EcoCyc"->{"http://biocyc.org/ECOLI/NEW-IMAGE?type=ENZYME&object=FRUCBISALD-CLASSII"}},
            "Comment"             -> {"According to EcoCyc, FBA Class II has the main activity for glycolysis. Thus, we neglect Class I."},
            "Scale"               -> 1],
Flux["E-FBA-syn",
        "Reactants"               -> 0 ,
        "Products"                -> "E-FBA",
        "Name"                    -> "enzyme synthesis",
        "Compartment"             -> "c",
        "Flux"                    -> s[P["FBA_FruR","Value"-> 2],1-c["FruR"][t]]*s[P["FBA_CRP","Value"-> 0],c["CRP"][t]],
        "Clamped"                 -> True],
Flux["TPI",
        "Reactants"               -> "dhap",
```

```
        "Products"                -> "g3p",
        "Name"                    -> "triose-phosphate isomerase",
        "Subsystem"               -> "Glycolysis/Gluconeogenesis",
        "EC"                      -> "5.3.1.1",
        "Compartment"             -> "c",
        "Link"                    -> {"EcoCyc"->"http://biocyc.org/ECOLI/NEW-IMAGE?type=REACTION&object=TRIOSEPISOMERIZATION-RXN"},
        "Resistance"              -> 0/c["E-TPI"][t]],
    Compound["E-TPI",
            "Compartment"         -> "c",
            "Name"                -> "enzyme of TPI",
            "Capacity"            -> 1,
            "Link"                -> {"EcoCyc"->"http://biocyc.org/ECOLI/NEW-IMAGE?type=ENZYME&object=TPI"},
            "Potential"           -> P["E-TPI_IC", "Value"->0.26265675028677777370*^+00001],
            "Scale"               -> 1],
    Flux["E-TPI-syn",
        "Reactants"               -> 0 ,
        "Products"                -> "E-TPI",
        "Name"                    -> "enzyme synthesis",
        "Compartment"             -> "c",
        "Flux"                    -> s[P["TPI_FruR","Value"-> 2],1-c["FruR"][t]],
        "Clamped"                 -> True],
    Flux["GAPD",
        "Reactants"               -> "g3p" + "nad" + "pi",
        "Products"                -> "13dpg" + "h" + "nadh",
        "Name"                    -> "glyceraldehyde-3-phosphate dehydrogenase",
        "Subsystem"               -> "Glycolysis/Gluconeogenesis",
        "EC"                      -> "1.2.1.12",
        "Link"                    -> {"EcoCyc"->"http://biocyc.org/ECOLI/NEW-IMAGE?type=REACTION&object=GAPOXNPHOSPHN-RXN"},
        "Compartment"             -> "c",
        "Resistance"              -> 0/c["E-GAPD"][t]],
    Compound["E-GAPD",
            "Compartment"         -> "c",
            "Name"                -> "enzyme of GAPD",
            "Capacity"            -> 1,
            "Potential"           -> P["E-GAPD_IC", "Value"->0.84651001525639313350*^+00000],
            "Link"                -> {"EcoCyc"->"http://biocyc.org/ECOLI/NEW-IMAGE?type=ENZYME&object=GAPDH-A-CPLX"},
            "Scale"               -> 1],
    Flux["E-GAPD-syn",
        "Reactants"               -> 0 ,
        "Products"                -> "E-GAPD",
        "Name"                    -> "enzyme synthesis",
        "Compartment"             -> "c",
        "Flux"                    -> s[P["GAPD_CRP","Value"-> 0],c["CRP"][t]]*s[P["GAPD_FruR","Value"-> 3],1-c["FruR"][t]],
        "Clamped"                 -> True],
    Flux["PGK",
        "Reactants"               -> "3pg" + "atp" ,
        "Products"                -> "13dpg" + "adp",
        "Name"                    -> "phosphoglycerate kinase",
        "Subsystem"               -> "Glycolysis/Gluconeogenesis",
        "EC"                      -> "2.7.2.3",
        "Link"                    -> {"EcoCyc"->"http://biocyc.org/ECOLI/NEW-IMAGE?type=REACTION&object=PHOSGLYPHOS-RXN"},
        "Compartment"             -> "c",
        "Resistance"              -> 0/c["E-PGK"][t]],
    Compound["E-PGK",
            "Compartment"         -> "c",
            "Name"                -> "enzyme of PGK",
            "Capacity"            -> 1,
            "Link"                -> {"EcoCyc"->"http://biocyc.org/ECOLI/NEW-IMAGE?type=ENZYME&object=PGK"},
            "Potential"           -> P["E-PGK_IC", "Value"->0.84651001525639313350*^+00000],
            "Scale"               -> 1],
    Flux["E-PGK-syn",
        "Reactants"               -> 0 ,
        "Products"                -> "E-PGK",
```

```
          "Name"                    -> "enzyme synthesis",
          "Compartment"             -> "c",
          "Flux"                    -> s[P["PGK_CRP","Value"-> 0],c["CRP"][t]]*s[P["PGK_FruR","Value"-> 3],1-c["FruR"][t]],
          "Clamped"                 -> True],
     Flux["PGM",
          "Reactants"               -> "2pg",
          "Products"                -> "3pg",
          "Name"                    -> "phosphoglycerate mutase",
          "Subsystem"               -> "Glycolysis/Gluconeogenesis",
          "EC"                      -> "5.4.2.1",
          "Link"                    -> {"EcoCyc" -> "http://biocyc.org/ECOLI/NEW-IMAGE?type=REACTION&object=3PGAREARR-RXN"},
          "Compartment"             -> "c",
          "Resistance"              -> 0/c["E-PGM"][t]],
     Compound["E-PGM",
              "Compartment"         -> "c",
              "Name"                -> "enzyme of PGM",
              "Capacity"            -> 1,
              "Link"                -> {"EcoCyc" -> "http://biocyc.org/ECOLI/NEW-IMAGE?type=ENZYME&object=PHOSGLYCMUTASE",
                                         "EcoCyc" -> "http://biocyc.org/ECOLI/NEW-IMAGE?type=ENZYME&object=PGMI-MONOMER"},

              "Potential"           -> P["E-PGM_IC", "Value"->0.4999977476448597357*^+00001],
              "Scale"               -> 1],
     Flux["E-PGM-syn",
          "Reactants"               -> 0 ,
          "Products"                -> "E-PGM",
          "Name"                    -> "enzyme synthesis",
          "Compartment"             -> "c",
          "Flux"                    -> 1,
          "Clamped"                 -> True],
     Flux["ENO",
          "Reactants"               -> "2pg",
          "Products"                -> "h2o" + "pep",
          "Name"                    -> "enolase",
          "Subsystem"               -> "Glycolysis/Gluconeogenesis",
          "EC"                      -> "4.2.1.11",
          "Link"                    -> {"EcoCyc" -> "http://biocyc.org/ECOLI/NEW-IMAGE?type=REACTION&object=2PGADEHYDRAT-RXN"},
          "Compartment"             -> "c",
          "Resistance"              -> 0/c["E-ENO"][t]],
     Compound["E-ENO",
              "Compartment"         -> "c",
              "Name"                -> "enzyme of ENO",
              "Capacity"            -> 1,
              "Potential"           -> P["E-ENO_IC", "Value"->0.1439862513558183509*^+00001],
              "Link"                -> {"EcoCyc" -> "http://biocyc.org/ECOLI/NEW-IMAGE?type=ENZYME&object=ENOLASE-CPLX"},
              "Scale"               -> 1],
     Flux["E-ENO-syn",
          "Reactants"               -> 0 ,
          "Products"                -> "E-ENO",
          "Name"                    -> "enzyme synthesis",
          "Compartment"             -> "c",
          "Flux"                    -> s[P["ENO_FruR","Value"-> 2],1-c["FruR"][t]],
          "Clamped"                 -> True],
     Flux["PYK",
          "Reactants"               -> "adp" + "h" + "pep",
          "Products"                -> "atp" + "pyr",
          "Name"                    -> "pyruvate kinase",
          "Subsystem"               -> "Glycolysis/Gluconeogenesis",
          "EC"                      -> "2.7.1.40",
          "Link"                    -> {"EcoCyc" -> "http://biocyc.org/ECOLI/NEW-IMAGE?type=REACTION&object=PEPDEPHOS-RXN"},
          "Compartment"             -> "c",
          "Resistance"              -> P["PYK_k","Value"-> 1*^-20]/(Cap["adp"]*Cap["h"]*Cap["pep"])/c["E-PYK"][t]*c["fdp"][t]^P["PYK_n_fdp","Value"-
> -2]],

     Compound["E-PYK",
              "Compartment"         -> "c",
```

```
         "Name"                   -> "enzyme of PYK",
         "Capacity"               -> 1,
         "Potential"              -> P["E-PYK_IC", "Value"->0.14398625135581835099*^+00001],
         "Link"                   -> {"EcoCyc" -> {"http://biocyc.org/ECOLI/NEW-IMAGE?type=ENZYME&object=PKI-COMPLEX"}},
         "Comment"                -> ("According to Ponce et al. (http://www.ncbi.nlm.nih.gov/pubmed/7559366) PykF (PYKI)"<>
                                      "has a much higher activity than PYKII. Thus we neglect PYKII"),
         "Scale"                  -> 1],
Flux["E-PYK-syn",
     "Reactants"                  -> 0 ,
     "Products"                   -> "E-PYK",
     "Name"                       -> "enzyme synthesis",
     "Compartment"                -> "c",
     "Flux"                       -> s[P["PYK_FruR","Value"-> 2],1-c["FruR"][t]],
     "Clamped"                    -> True],
Flux["PPS",
     "Reactants"                  -> "atp" + "pyr" +"h2o",
     "Products"                   -> "amp" + 2 "h" + "pep" + "pi",
     "Name"                       -> "phosphoenolpyruvate synthase",
     "Subsystem"                  -> "Glycolysis/Gluconeogenesis",
     "EC"                         -> "2.7.9.2",
     "Link"                       -> {"EcoCyc"->"http://biocyc.org/ECOLI/NEW-IMAGE?type=REACTION&object=PEPSYNTH-RXN"},
     "Compartment"                -> "c",
     "Resistance"                 -> P["PPS_k","Value"-> 2.5*^-5]/(Cap["atp"]*Cap["pyr"]*Cap["h2o"])/c["E-PPS"][t]
    ],
Compound["E-PPS",
         "Compartment"            -> "c",
         "Name"                   -> "enzyme of PPS",
         "Link"                   -> {"EcoCyc"->"http://biocyc.org/ECOLI/NEW-IMAGE?type=ENZYME&object=PEPSYNTH-CPLX"},
         "Capacity"               -> 1,
         "Potential"              -> P["E-PPS_IC", "Value"->0.48101093269028538657*^+00001],
         "Scale"                  -> 1],
Flux["E-PPS-syn",
     "Reactants"                  -> 0 ,
     "Products"                   -> "E-PPS",
     "Name"                       -> "enzyme synthesis",
     "Compartment"                -> "c",
     "Flux"                       -> s[P["PPS_FruR","Value"-> 2],c["FruR"][t]],
     "Clamped"                    -> True],
Flux["PDH",
     "Reactants"                  -> "coa" + "nad" + "pyr",
     "Products"                   -> "accoa" + "co2" + "nadh",
     "Name"                       -> "pyruvate dehydrogenase",
     "Subsystem"                  -> "Glycolysis/Gluconeogenesis",
     "Compartment"                -> "c",
     "Link"                       -> {"EcoCyc" -> "http://biocyc.org/ECOLI/NEW-IMAGE?type=REACTION&object=PYRUVDEH-RXN"},
     "EC"                         -> "1.2.1",
     "Resistance"                 -> ((P["PDH_k","Value"-> 1.0*^-5]/(Cap["coa"]*Cap["nad"]*Cap["pyr"])/c["E-PDH"][t])
                                      *c["nadh"][t]^P["PDH_n_nadh","Value"-> 1])],
Compound["E-PDH",
         "Compartment"            -> "c",
         "Link"                   -> {"EcoCyc" -> "http://biocyc.org/ECOLI/NEW-IMAGE?type=ENZYME&object=PYRUVATEDEH-CPLX"},
         "Name"                   -> "enzyme of PDH",
         "Capacity"               -> 1,
         "Potential"              -> P["E-PDH_IC", "Value"->0.25004278565895567965*^+00001],
         "Scale"                  -> 1],
Flux["E-PDH-syn",
     "Reactants"                  -> 0 ,
     "Products"                   -> "E-PDH",
     "Name"                       -> "enzyme synthesis",
     "Compartment"                -> "c",
     "Flux"                       -> (s[P["PDH_FNR","Value"-> 1],1-c["FNR"][t]]*s[P["PDH_PdhR","Value"-> 2],1-c["PdhR"][t]]*
                                      s[P["PDH_ArcA","Value"-> 1],1-c["ArcA"][t]]*s[P["PDH_CRP","Value"-> 1],c["CRP"][t]]),
     "Clamped"                    -> True],
```

```
(* ***  Entner-Doudoroff pathway                                                *** *)
Flux["EDD",
     "Reactants"              -> "6pgc",
     "Products"               -> "2ddg6p" + "h2o",
     "Name"                   -> "6-phosphogluconate dehydratase",
     "Compartment"            -> "c",
     "Subsystem"              -> "Pentose Phosphate Cycle",
     "Link"                   -> {"EcoCyc" -> "http://biocyc.org/ECOLI/NEW-IMAGE?type=REACTION-IN-PATHWAY&object=PGLUCONDEHYDRAT-RXN"},
     "EC"                     -> "4.2.1.12",
     "Resistance"             -> P["EDD_k","Value"-> 1.0*^-7]/(Cap["6pgc"])],
Compound["E-EDD",
         "Compartment"        -> "c",
         "Link"               -> {"EcoCyc" -> "http://biocyc.org/ECOLI/NEW-IMAGE?type=ENZYME&object=PGLUCONDEHYDRAT-MONOMER"},
         "Name"               -> "enzyme of EDD",
         "Capacity"           -> 1,
         "Potential"          -> P["E-EDD_IC", "Value"->0.26265675028677777370*^+00001],
         "Scale"              -> 1],
Flux["E-EDD-syn",
     "Reactants"              -> 0 ,
     "Products"               -> "E-EDD",
     "Name"                   -> "enzyme synthesis",
     "Compartment"            -> "c",
     "Flux"                   -> s[P["EDD_FruR","Value"-> 2],1-c["FruR"][t]],
     "Clamped"                -> True],

Flux["EDA",
     "Reactants"              -> "2ddg6p",
     "Products"               -> "g3p" + "pyr",
     "Name"                   -> "2-dehydro-3-deoxy-phosphogluconate aldolase",
     "Subsystem"              -> "Pentose Phosphate Cycle",
     "Link"                   -> {"EcoCyc" -> "http://biocyc.org/ECOLI/NEW-IMAGE?type=REACTION&object=KDPGALDOL-RXN"},
     "EC"                     -> "4.1.2.14",
     "Resistance"             -> 0/Cap["2ddg6p"],
     "Compartment"            -> "c"],
Compound["E-EDA",
         "Compartment"        -> "c",
         "Link"               -> {"EcoCyc" -> "http://biocyc.org/ECOLI/NEW-IMAGE?type=GENE&object=EG10256"},
         "Name"               -> "enzyme of EDA",
         "Capacity"           -> 1,
         "Potential"          -> P["E-EDA_IC", "Value"->0.26265675028677777370*^+00001],
         "Scale"              -> 1],
Flux["E-EDA-syn",
     "Reactants"              -> 0 ,
     "Products"               -> "E-EDA",
     "Name"                   -> "enzyme synthesis",
     "Compartment"            -> "c",
     "Flux"                   -> s[P["EDA_FruR","Value"-> 2],1-c["FruR"][t]],
     "Clamped"                -> True],

(* *** Citrate Cyle (TCA)                                                       *** *)
Flux["CS",
     "Reactants"              -> "accoa" + "h2o" + "oaa",
     "Products"               -> "cit" + "coa" + "h",
     "Name"                   -> "citrate synthase",
     "Subsystem"              -> "Citrate Cycle (TCA)",
     "EC"                     -> "2.3.3.1",
     "Link"                   -> {"EcoCyc" -> "http://biocyc.org/ECOLI/NEW-IMAGE?type=REACTION&object=CITSYN-RXN"},
     "Compartment"            -> "c",
     "Resistance"             -> P["CS_k","Value"-> 1.0*^-10]/(Cap["accoa"]*Cap["h2o"]*Cap["oaa"])/c["E-CS"][t],
     "Link"                   -> {"EcoCyc" -> "http://biocyc.org/ECOLI/NEW-IMAGE?type=REACTION&object=CITSYN-RXN"}],
Compound["E-CS",
         "Compartment"        -> "c",
```

```
          "Link"                -> {"EcoCyc" -> "http://biocyc.org/ECOLI/NEW-IMAGE?type=GENE&object=EG10402"},
          "Name"                -> "enzyme of CS",
          "Capacity"            -> 1,
          "Potential"           -> P["E-CS_IC", "Value"->0.499857701747602067 85*^+00001],
          "Scale"               -> 1],
Flux["E-CS-syn",
     "Reactants"                -> 0 ,
     "Products"                 -> "E-CS",
     "Name"                     -> "enzyme synthesis",
     "Compartment"              -> "c",
     "Flux"                     -> s[P["CS_ArcA","Value"-> 1],1-c["ArcA"][t]]*s[P["CS_CRP","Value"-> 1],c["CRP"][t]],
     "Clamped"                  -> True],

Flux["ACONT",
     "Reactants"                -> "cit",
     "Products"                 -> "icit",
     "Name"                     -> "aconitase",
     "Subsystem"                -> "Citrate Cycle (TCA)",
     "EC"                       -> "4.2.1.3",
     "Link"                     -> {"EcoCyc" -> "http://biocyc.org/ECOLI/NEW-IMAGE?type=REACTION&object=ACONITATEDEHYDR-RXN"},
     "Compartment"              -> "c",
     "Resistance"               -> 0/c["E-ACONT"][t]],
Compound["E-ACONT",
          "Compartment"         -> "c",
          "Name"                -> "enzyme of ACONT",
          "Link"                -> {"EcoCyc" -> "http://biocyc.org/ECOLI/NEW-IMAGE?type=ENZYME&object=CPLX0-7761"},
          "Comment"             -> {"According to EcoCyc AcnB appears to be the main catabolic enzyme. Thus, we neglect AcnA"},
          "Capacity"            -> 1,
          "Potential"           -> P["E-ACONT_IC", "Value"->0.49985770174760206785*^+00001],
          "Scale"               -> 1],
Flux["E-ACONT-syn",
     "Reactants"                -> 0 ,
     "Products"                 -> "E-ACONT",
     "Name"                     -> "enzyme synthesis",
     "Compartment"              -> "c",
     "Flux"                     -> s[P["ACONT_ArcA","Value"-> 1],1-c["ArcA"][t]]*s[P["ACONT_CRP","Value"-> 1],c["CRP"][t]],
     "Clamped"                  -> True],
Flux["ICDHyr",
     "Reactants"                -> "icit" + "nadp",
     "Products"                 -> "akg" + "co2" + "nadph",
     "Name"                     -> "isocitrate dehydrogenase (NADP)",
     "Subsystem"                -> "Citrate Cycle (TCA)",
     "EC"                       -> "1.1.1.42",
     "Link"                     -> {"EcoCyc"->"http://biocyc.org/ECOLI/NEW-IMAGE?type=REACTION&object=ISOCITDEH-RXN"},
     "Compartment"              -> "c",
     "Resistance"               -> 0/c["E-ICDHyr"][t]],
Compound["E-ICDHyr",
          "Compartment"         -> "c",
          "Name"                -> "enzyme of ICDHyr",
          "Capacity"            -> 1,
          "Link"                -> {"EcoCyc" -> "http://biocyc.org/ECOLI/NEW-IMAGE?type=ENZYME&object=ISOCITHASE-CPLX"},
          "Potential"           -> P["E-ICDHyr_IC", "Value"->0.49999774760284241282*^+00001],
          "Scale"               -> 1],
Flux["E-ICDHyr-syn",
     "Reactants"                -> 0 ,
     "Products"                 -> "E-ICDHyr",
     "Name"                     -> "enzyme synthesis",
     "Compartment"              -> "c",
     "Flux"                     -> s[P["ICDHyr_ArcA","Value"-> 2],1-c["ArcA"][t]]*s[P["ICDHyr_FruR","Value"-> 0],c["FruR"][t]],
     "Clamped"                  -> True],
Flux["AKGDH",
     "Reactants"                -> "akg" + "coa" + "nad",
     "Products"                 -> "co2" + "nadh" + "succoa",
```

```
      "Name"                    -> "2-Oxogluterate dehydrogenase",
      "Subsystem"               -> "Citrate Cycle (TCA)",
      "Compartment"             -> "c",
      "Resistance"              -> P["AKGDH_k","Value"-> 1*^-9]/(Cap["akg"]*Cap["coa"]*Cap["nad"])/c["E-AKGDH"][t],
      "Link"                    -> {"EcoCyc" -> "http://biocyc.org/ECOLI/NEW-IMAGE?type=REACTION&object=2OXOGLUTARATEDEH-RXN"}],
Compound["E-AKGDH",
          "Compartment"         -> "c",
          "Name"                -> "enzyme of AKGDH",
          "Link"                -> {"EcoCyc" -> "http://biocyc.org/ECOLI/NEW-IMAGE?type=ENZYME&object=2OXOGLUTARATEDEH-CPLX"},
          "Capacity"            -> 1,
          "Potential"           -> P["E-AKGDH_IC", "Value"->0.25014788266203766831*^+00001],
          "Scale"               -> 1],
Flux["E-AKGDH-syn",
      "Reactants"               -> 0 ,
      "Products"                -> "E-AKGDH",
      "Name"                    -> "enzyme synthesis",
      "Compartment"             -> "c",
      "Flux"                    -> (s[P["AKGDH_ArcA","Value"-> 2],1-c["ArcA"][t]]*s[P["AKGDH_FNR","Value"-> 2],1-c["FNR"][t]]*
                                    s[P["AKGDH_PdhR","Value"-> 1],1-c["PdhR"][t]]),
      "Clamped"                 -> True],
Flux["SUCOAS",
      "Reactants"               -> "atp" + "coa" + "succ",
      "Products"                -> "adp" + "pi" + "succoa",
      "Name"                    -> "succinyl-CoA synthetase (ADP-forming)",
      "Subsystem"               -> "Citrate Cycle (TCA)",
      "EC"                      -> "6.2.1.5",
      "Link"                    -> {"EcoCyc" -> "http://biocyc.org/ECOLI/NEW-IMAGE?type=REACTION&object=SUCCCOASYN-RXN"},
      "Compartment"             -> "c",
      "Resistance"              -> 0/c["E-SUCOAS"][t]],
Compound["E-SUCOAS",
          "Compartment"         -> "c",
          "Name"                -> "enzyme of SUCOAS",
          "Link"                -> {"EcoCyc" -> "http://biocyc.org/ECOLI/NEW-IMAGE?type=ENZYME&object=SUCCCOASYN"},
          "Capacity"            -> 1,
          "Potential"           -> P["E-SUCOAS_IC", "Value"->0.49978304187044528817*^+00001],
          "Scale"               -> 1],
Flux["E-SUCOAS-syn",
      "Reactants"               -> 0 ,
      "Products"                -> "E-SUCOAS",
      "Name"                    -> "enzyme synthesis of SUCOAS",
      "Compartment"             -> "c",
      "Flux"                    -> (s[P["SUCOAS_FNR","Value"-> 1],1-c["FNR"][t]]*s[P["SUCOAS_ArcA","Value"-> 1],1-c["ArcA"][t]]*
                                    s[P["SUCOAS_CRP","Value"-> 1],c["CRP"][t]]),
      "Clamped"                 -> True],

Flux["SUCDH",
      "Reactants"               -> "q8" + "succ",
      "Products"                -> "q8h2" + "fum",
      "Name"                    -> "succinate dehydrogenase",
      "Subsystem"               -> "Citrate Cycle (TCA)",
      "EC"                      -> "1.3.5.1",
      "Link"                    -> {"EcoCyc" -> "http://biocyc.org/ECOLI/NEW-IMAGE?type=REACTION&object=SUCCINATE-DEHYDROGENASE-UBIQUINONE-RXN"}
,
      "Compartment"             -> "cm",
      "Resistance"              -> P["SUCDH_k","Value"-> 1.0*^1]*(c["q8h2"][t]+c["q8"][t])/c["E-SUCDH"][t]/(Cap["q8"]*Cap["succ"])],
Compound["E-SUCDH",
          "Compartment"         -> "cm",
          "Name"                -> "enzyme of SUCDH",
          "Link"                -> {"EcoCyc" -> "http://biocyc.org/ECOLI/NEW-IMAGE?type=ENZYME&object=SUCC-DEHASE"},
          "Capacity"            -> 1,
          "Potential"           -> P["E-SUCDH_IC", "Value"->0.55816839228624992586*^+00001],
          "Scale"               -> 1],
Flux["E-SUCDH-syn",
```

```
      "Reactants"             -> 0 ,
      "Products"              -> "E-SUCDH",
      "Name"                  -> "enzyme synthesis of SUCDH",
      "Compartment"           -> "c",
      "Flux"                  -> (s[P["SUCDH_FNR","Value"-> 3],1-c["FNR"][t]]*s[P["SUCDH_ArcA","Value"-> 3],1-c["ArcA"][t]]*
                                  s[P["SUCDH_CRP","Value"-> 3],c["CRP"][t]]),
      "Clamped"               -> True],

Flux["FRD",
      "Reactants"             -> "mqn8" + "succ",
      "Products"              -> "mql8" + "fum",
      "Name"                  -> "succinate dehydrogenase",
      "Subsystem"             -> "Citrate Cycle (TCA)",
      "EC"                    -> "1.3.5.4",
      "Link"                  -> {"EcoCyc" -> "http://biocyc.org/ECOLI/NEW-IMAGE?type=REACTION&object=R601-RXN"},
      "Compartment"           -> "cm",
      "Comment"               -> "",
      "Resistance"            -> P["FRD_k","Value"-> 1.0*^0]*(c["mql8"][t]+c["mqn8"][t])/c["E-FRD"][t]/(Cap["mqn8"]*Cap["succ"])],
Compound["E-FRD",
          "Compartment"       -> "c",
          "Name"              -> "enzyme of FRD",
          "Capacity"          -> 1,
          "Link"              -> {"EcoCyc" -> "http://biocyc.org/ECOLI/NEW-IMAGE?type=ENZYME&object=FUMARATE-REDUCTASE"},
          "Potential"         -> P["E-FRD_IC", "Value"->0.19437993052318582876*^+00001],
          "Scale"             -> 1],
Flux["E-FRD-syn",
      "Reactants"             -> 0 ,
      "Products"              -> "E-FRD",
      "Name"                  -> "enzyme synthesis of FRD",
      "Compartment"           -> "c",
      "Flux"                  -> s[P["FRD_FNR","Value"-> 1],c["FNR"][t]],
      "Clamped"               -> True],

Flux["FUM",
      "Reactants"             -> "fum" + "h2o",
      "Products"              -> "mal",
      "Name"                  -> "fumarase",
      "Subsystem"             -> "Citrate Cycle (TCA)",
      "EC"                    -> "4.2.1.2",
      "Link"                  -> {"EcoCyc" -> "http://biocyc.org/ECOLI/NEW-IMAGE?type=REACTION&object=FUMHYDR-RXN"},
      "Compartment"           -> "c",
      "Resistance"            -> 0],
Compound["E-FUM",
          "Compartment"       -> "c",
          "Name"              -> "enzyme of FUM",
          "Link"              -> {"EcoCyc" -> "http://biocyc.org/ECOLI/NEW-IMAGE?type=ENZYME&object=FUMARASE-A"},
          "Comment"           -> ("Because fumA mRNA shows the highest differential expression" <>
                                  "(compared with fumB and fumC), we assume that the main activity is due to fumA."),

          "Capacity"          -> 1,
          "Potential"         -> P["E-FUM_IC", "Value"->0.49978304187044528817*^+00001],
          "Scale"             -> 1],
Flux["E-FUM-syn",
      "Reactants"             -> 0 ,
      "Products"              -> "E-FUM",
      "Name"                  -> "enzyme synthesis of FUM",
      "Compartment"           -> "c",
      "Flux"                  -> (s[P["FUM_FNR","Value"-> 2],1-c["FNR"][t]]*s[P["FUM_ArcA","Value"-> 2],1-c["ArcA"][t]]*
                                  s[P["FUM_CRP","Value"-> 2],c["CRP"][t]]),
      "Clamped"               -> True],

Flux["MDH",
      "Reactants"             -> "mal" + "nad",
      "Products"              -> "h" + "nadh" + "oaa",
```

```
      "Name"                     -> "malate dehydrogenase",
      "Subsystem"                -> "Citrate Cycle (TCA)",
      "EC"                       -> "1.1.1.37",
      "Link"                     -> {"EcoCyc" -> "http://biocyc.org/ECOLI/NEW-IMAGE?type=REACTION&object=MALATE-DEH-RXN"},
      "Compartment"              -> "c",
      "Resistance"               -> 0/c["E-MDH"][t]],
Compound["E-MDH",
          "Compartment"          -> "c",
          "Name"                 -> "enzyme of MDH",
          "Capacity"             -> 1,
          "Link"                 -> {"EcoCyc" -> "http://biocyc.org/ECOLI/NEW-IMAGE?type=ENZYME&object=MALATE-DEHASE"},
          "Potential"            -> P["E-MDH_IC", "Value"->0.49975266734090304510*^+00001],
          "Scale"                -> 1],
Flux["E-MDH-syn",
      "Reactants"                -> 0 ,
      "Products"                 -> "E-MDH",
      "Name"                     -> "enzyme synthesis of MDH",
      "Compartment"              -> "c",
      "Flux"                     -> s[P["MDH_CRP","Value"-> 1],c["CRP"][t]]*s[P["MDH_ArcA","Value"-> 0],1-c["ArcA"][t]],
      "Clamped"                  -> True],

Flux["MQO(q8)",
      "Reactants"                -> "mal" + "q8",
      "Products"                 -> "q8h2" + "oaa",
      "Name"                     -> "malate dehydrogenase",
      "Subsystem"                -> "Citrate Cycle (TCA)",
      "EC"                       -> "1.1.5.4",
      "Link"                     -> {"EcoCyc" -> "http://www.ecocyc.org/ECOLI/NEW-IMAGE?type=REACTION&object=MALATE-DEHYDROGENASE-ACCEPTOR-RXN"},
      "Compartment"              -> "c",
      "Resistance"               -> P["MQO(q8)_k","Value"-> 1]*(c["q8h2"][t]+c["q8"][t])/(Cap["mal"]*Cap["q8"])/c["E-MQO"][t]],
Flux["MQO(mqn8)",
      "Reactants"                -> "mal" + "mqn8",
      "Products"                 -> "mql8" + "oaa",
      "Name"                     -> "malate dehydrogenase",
      "Subsystem"                -> "Citrate Cycle (TCA)",
      "EC"                       -> "1.1.5.4",
      "Link"                     -> {"EcoCyc" -> "http://www.ecocyc.org/ECOLI/NEW-IMAGE?type=REACTION&object=MALATE-DEHYDROGENASE-ACCEPTOR-RXN"},
      "Compartment"              -> "c",
      "Resistance"               -> P["MQO(mqn8)_k","Value"-> 1*^-1]*(c["mql8"][t]+c["mqn8"][t])/(Cap["mal"]*Cap["mqn8"])/c["E-MQO"][t]],
Compound["E-MQO",
          "Compartment"          -> "c",
          "Name"                 -> "enzyme of MQO",
          "Capacity"             -> 1,
          "Link"                 -> {"EcoCyc" -> "http://www.ecocyc.org/ECOLI/NEW-IMAGE?type=ENZYME&object=EG12069-MONOMER"},
          "Potential"            -> P["E-MQO_IC", "Value"->0.49999774764485973577*^+00001],
          "Scale"                -> 1],
Flux["E-MQO-syn",
      "Reactants"                -> 0 ,
      "Products"                 -> "E-MQO",
      "Name"                     -> "enzyme synthesis of MQO",
      "Compartment"              -> "c",
      "Flux"                     -> 1,
      "Clamped"                  -> True],

(* *** glyoxylate shunt/cycle                              *** *)
Flux["ICL",
      "Reactants"                -> "icit",
      "Products"                 -> "glx" + "succ",
      "Name"                     -> "isocitrate lyase",
      "Subsystem"                -> "Anaplerotic Reactions",
      "EC"                       -> "4.1.3.1",
      "Link"                     -> {"EcoCyc" -> "http://biocyc.org/ECOLI/NEW-IMAGE?type=REACTION&object=ISOCIT-CLEAV-RXN"},
      "Compartment"              -> "c",
```

```
     "Resistance"              -> 0/(Cap["icit"])/c["E-ICL"][t]],
Compound["E-ICL",
         "Compartment"         -> "c",
         "Name"                -> "enzyme of ICL",
         "Link"                -> {"EcoCyc" -> "http://biocyc.org/ECOLI/NEW-IMAGE?type=ENZYME&object=ISOCIT-LYASE"},
         "Capacity"            -> 1,
         "Potential"           -> P["E-ICL_IC", "Value"->0.47783515494725001815*^+00001],
         "Scale"               -> 1],
Flux["E-ICL-syn",
     "Reactants"               -> 0 ,
     "Products"                -> "E-ICL",
     "Name"                    -> "enzyme synthesis of ICL",
     "Compartment"             -> "c",
     "Flux"                    -> (s[P["ICL_IclR","Value"-> 2],1-c["IclR"][t]]*s[P["ICL_FruR","Value"-> 2],c["FruR"][t]]*
                                   s[P["ICL_CRP","Value"-> 1],1-c["CRP"][t]]),
     "Clamped"                 -> True],

Flux["MALS",
     "Reactants"               -> "accoa" + "glx" + "h2o",
     "Products"                -> "coa" + "h" + "mal",
     "Name"                    -> "malate synthase",
     "EC"                      -> "2.3.3.9",
     "Link"                    -> {"EcoCyc" -> "http://biocyc.org/ECOLI/NEW-IMAGE?type=REACTION&object=MALSYN-RXN"},
     "Compartment"             -> "c",
     "Resistance"              -> P["MALS_k","Value"-> 1*^-9]/(Cap["accoa"]*Cap["glx"]*Cap["h2o"])/c["E-MALS"][t]],
Compound["E-MALS",
         "Compartment"         -> "c",
         "Name"                -> "enzyme of MALS",
         "Link"                -> {"EcoCyc" -> "http://biocyc.org/ECOLI/NEW-IMAGE?type=ENZYME&object=MALATE-SYNTHASE"},
         "Comment"             -> ("We assume that aceB carries the main activity under our conditions and glcB" <>
                                   "is mainly responsible for growth on glycolate (see EcoCyc)"),
         "Capacity"            -> 1,
         "Potential"           -> P["E-MALS_IC", "Value"->0.48733218219641045721*^+00001],
         "Scale"               -> 1],
Flux["E-MALS-syn",
     "Reactants"               -> 0 ,
     "Products"                -> "E-MALS",
     "Name"                    -> "enzyme synthesis of MALS",
     "Compartment"             -> "c",
     "Flux"                    -> (s[P["MALS_FruR","Value"-> 1],c["FruR"][t]]*s[P["MALS_ArcA","Value"-> 1],1-c["ArcA"][t]]*
                                   s[P["MALS_IclR","Value"-> 1],1-c["IclR"][t]]*s[P["MALS_CRP","Value"-> 1],1-c["CRP"][t]]),
     "Clamped"                 -> True],

(* *** Reductive TCA                                                              *** *)
Flux["PPCK",
     "Reactants"               -> "atp" + "oaa",
     "Products"                -> "adp" + "co2" + "pep",
     "Name"                    -> "phosphoenolpyruvate carboxykinase",
     "Subsystem"               -> "Anaplerotic reactions",
     "EC"                      -> "4.1.1.49",
     "Link"                    -> {"EcoCyc"->"http://biocyc.org/ECOLI/NEW-IMAGE?type=REACTION&object=PEPCARBOXYKIN-RXN"},
     "Compartment"             -> "c",
     "Resistance"              -> P["PPCK_k","Value"-> 1.0*^-8]/(Cap["atp"]*Cap["oaa"])/c["E-PPCK"][t]
    ],
Compound["E-PPCK",
         "Compartment"         -> "c",
         "Name"                -> "enzyme of PPCK",
         "Capacity"            -> 1,
         "Potential"           -> P["E-PPCK_IC", "Value"->0.48101093269028538657*^+00001],
         "Link"                -> {"EcoCyc"->"http://biocyc.org/ECOLI/NEW-IMAGE?type=GENE&object=EG10688"},
         "Scale"               -> 1],
Flux["E-PPCK-syn",
     "Reactants"               -> 0 ,
```

```
          "Products"                 -> "E-PPCK",
          "Name"                     -> "enzyme synthesis",
          "Compartment"              -> "c",
          "Flux"                     -> s[P["PPCK_FruR","Value"-> 2],c["FruR"][t]],
          "Clamped"                  -> True],

     Flux["PPC",
          "Reactants"                -> "co2" + "h2o" + "pep",
          "Products"                 -> "h" + "oaa" + "pi",
          "Name"                     -> "phosphoenolpyruvate carboxylase",
          "Subsystem"                -> "Anaplerotic reactions",
          "EC"                       -> "4.1.1.31",
          "Link"                     -> {"EcoCyc"->"http://biocyc.org/ECOLI/NEW-IMAGE?type=REACTION&object=PEPCARBOX-RXN"},
          "Compartment"              -> "c",
          "Resistance"               -> P["PPC_k","Value"-> 1.0*^-1]/(Cap["co2"]*Cap["h2o"]*Cap["pep"])/c["E-PPC"][t]*c["mal"][t]^P["PPC_n_mal","Valu
e"-> 2],
          "Link"                     -> {"EcoCyc" -> "http://biocyc.org/ECOLI/NEW-IMAGE?type=REACTION&object=PEPCARBOX-RXN"}],
     Compound["E-PPC",
              "Compartment"          -> "c",
              "Name"                 -> "enzyme of PPC",
              "Capacity"             -> 1,
              "Link"                 -> {"EcoCyc"->"http://biocyc.org/ECOLI/NEW-IMAGE?type=ENZYME&object=PEPCARBOX-CPLX"},
              "Potential"            -> P["E-PPC_IC", "Value"->0.14398625135581835099*^+00001],
              "Scale"                -> 1],
     Flux["E-PPC-syn",
          "Reactants"                -> 0 ,
          "Products"                 -> "E-PPC",
          "Name"                     -> "enzyme synthesis",
          "Compartment"              -> "c",
          "Flux"                     -> s[P["PPC_FruR","Value"-> 2],1-c["FruR"][t]],
          "Clamped"                  -> True],


     (* *** Pyruvate Metabolism                                                   *** *)
     Flux["LDH",
          "Reactants"                -> "lac" + "nad",
          "Products"                 -> "h" + "nadh" + "pyr",
          "Name"                     -> "D-lactate dehydrogenase",
          "Subsystem"                -> "Pyruvate metabolism",
          "EC"                       -> "1.1.1.28",
          "Link"                     -> {"EcoCyc" -> "http://www.ecocyc.org/ECOLI/NEW-IMAGE?type=REACTION&object=DLACTDEHYDROGNAD-RXN"},
          "Resistance"               -> P["LDH_k","Value"-> 1]/(Cap["pyr"]*Cap["nadh"])/c["E-LDH"][t],
          "Compartment"              -> "c"],
     Compound["E-LDH",
              "Compartment"          -> "c",
              "Name"                 -> "enzyme of LDH",
              "Capacity"             -> 1,
              "Link"                 -> {"EcoCyc" -> "http://www.ecocyc.org/ECOLI/NEW-IMAGE?type=ENZYME&object=DLACTDEHYDROGNAD-MONOMER"},
              "Potential"            -> P["E-LDH_IC", "Value"->0.49999774764485973577*^+00001],
              "Scale"                -> 1],
     Flux["E-LDH-syn",
          "Reactants"                -> 0 ,
          "Products"                 -> "E-LDH",
          "Name"                     -> "enzyme synthesis",
          "Compartment"              -> "c",

          "Flux"                     ->  s[P["LDH_ArcA","Value"-> 1],1-c["ArcA"][t]],
          "Clamped"                  -> True],
     Flux["POX",
          "Reactants"                -> "h2o" + "pyr" + "q8",
          "Products"                 -> "ac" + "co2" + "q8h2",
          "Name"                     -> "pyruvate oxidase",
          "Subsystem"                -> "oxidative phophorylation",
```

```
     "EC"                        -> "1.2.5.1",
     "Link"                      -> {"EcoCyc"->"http://biocyc.org/ECOLI/NEW-IMAGE?type=REACTION&object=RXN-11496"},
     "Compartment"               -> "c",
     "Resistance"                -> P["POX_k","Value"-> 1]*(c["q8h2"][t]+c["q8"][t])/(Cap["h2o"]*Cap["pyr"]*Cap["q8"])/c["E-POX"][t]],
Compound["E-POX",
         "Compartment"           -> "c",
         "Name"                  -> "enzyme of POX",
         "Capacity"              -> 1,
         "Link"                  -> {"EcoCyc"->"http://biocyc.org/ECOLI/NEW-IMAGE?type=ENZYME&object=PYRUVOXID-CPLX"},
         "Potential"             -> P["E-POX_IC", "Value"->0.49999774764485973577*^+00001],
         "Scale"                 -> 1],
Flux["E-POX-syn",
     "Reactants"                 -> 0 ,
     "Products"                  -> "E-POX",
     "Name"                      -> "enzyme synthesis",
     "Compartment"               -> "c",
     "Flux"                      -> 1,
     "Clamped"                   -> True],

Flux["ACS",
     "Reactants"                 -> "ac" + "atp" + "coa",
     "Products"                  -> "accoa" + "amp" + "ppi",
     "Name"                      -> "acetyl-CoA synthetase",
     "Subsystem"                 -> "Pyruvate metabolism",
     "EC"                        -> "6.2.1.1",
     "Link"                      -> {"EcoCyc"->"http://biocyc.org/ECOLI/NEW-IMAGE?type=REACTION&object=ACETATE--COA-LIGASE-RXN"},
     "Compartment"               -> "c",
     "Resistance"                -> P["ACS_k","Value"-> 1*^-8]/(Cap["ac"]*Cap["atp"]*Cap["coa"])/c["E-ACS"][t]],
Compound["E-ACS",
         "Compartment"           -> "c",
         "Name"                  -> "enzyme of ACS",
         "Capacity"              -> 1,
         "Link"                  -> {"EcoCyc"->"http://biocyc.org/ECOLI/NEW-IMAGE?type=GENE&object=EG11448"},
         "Potential"             -> P["E-ACS_IC", "Value"->0.49975266736257850653*^+00001],
         "Scale"                 -> 1],
Flux["E-ACS-syn",
     "Reactants"                 -> 0 ,
     "Products"                  -> "E-ACS",
     "Name"                      -> "enzyme synthesis",
     "Compartment"               -> "c",
     "Flux"                      -> s[P["ACS_CRP","Value"-> 10],c["CRP"][t]],
     "Clamped"                   -> True],

Flux["PTAr",
     "Reactants"                 -> "accoa" + "pi",
     "Products"                  -> "actp" + "coa",
     "Name"                      -> "phosphotransacetylase",
     "Subsystem"                 -> "Pyruvate metabolism",
     "EC"                        -> "2.3.1.8",
     "Link"                      -> {"EcoCyc"->"http://biocyc.org/ECOLI/NEW-IMAGE?type=REACTION&object=PHOSACETYLTRANS-RXN"},
     "Compartment"               -> "c",
     "Resistance"                -> (P["PTAr_k","Value"-> 1.0*^-6]/(Cap["accoa"]*Cap["pi"])/c["E-PTAr"][t])],
Compound["E-PTAr",
         "Compartment"           -> "c",
         "Name"                  -> "enzyme of PTAr",
         "Capacity"              -> 1,
         "Link"                  -> {"EcoCyc"->"http://biocyc.org/ECOLI/NEW-IMAGE?type=ENZYME&object=PHOSACETYLTRANS-CPLX"},
         "Potential"             -> P["E-PTAr_IC", "Value"->0.49999774764485973577*^+00001],
         "Scale"                 -> 1],
Flux["E-PTAr-syn",
     "Reactants"                 -> 0 ,
     "Products"                  -> "E-PTAr",
     "Name"                      -> "enzyme synthesis",
```

```
     "Compartment"             -> "c",
     "Flux"                    -> 1,
     "Clamped"                 -> True],

Flux["ACKr",
     "Reactants"               -> "ac" + "atp",
     "Products"                -> "actp" + "adp",
     "Name"                    -> "acetate kinase",
     "Subsystem"               -> "Pyruvate metabolism",
     "EC"                      -> "2.7.2.1",
     "Link"                    -> {"EcoCyc"->"http://biocyc.org/ECOLI/NEW-IMAGE?type=REACTION&object=ACETATEKIN-RXN"},
     "Compartment"             -> "c",
     "Resistance"              -> 0/c["E-ACKr"][t]],
Compound["E-ACKr",
         "Compartment"         -> "c",
         "Name"                -> "enzyme of ACKr",
         "Capacity"            -> 1,
         "Potential"           -> P["E-ACKr_IC", "Value"->0.25000015482243731491*^+00001],
         "Link"                -> {"EcoCyc"->"http://biocyc.org/ECOLI/NEW-IMAGE?type=ENZYME&object=ACETATEKINA-MONOMER"},
         "Scale"               -> 1],
Flux["E-ACKr-syn",
     "Reactants"               -> 0 ,
     "Products"                -> "E-ACKr",
     "Name"                    -> "enzyme synthesis",
     "Compartment"             -> "c",
     "Flux"                    -> s[P["ACKr_FNR","Value"-> 1],c["FNR"][t]]*s[P["ACKr_ArcA","Value"-> 0],c["ArcA"][t]],
     "Clamped"                 -> True],

Flux["PFL",
     "Reactants"               -> "coa" + "pyr",
     "Products"                -> "accoa" + "for",
     "Name"                    -> "pyruvate formate lyase",
     "Subsystem"               -> "Pyruvate metabolism",
     "Compartment"             -> "c",
     "Link"                    -> {"EcoCyc" -> "http://biocyc.org/ECOLI/NEW-IMAGE?type=REACTION&object=PYRUVFORMLY-RXN"},
     "EC"                      -> "2.3.1.54",
     "Resistance"              -> P["PFL_k","Value"-> 1.0*^-7]/c["E-PFL"][t]/(Cap["coa"]*Cap["pyr"])],
Compound["E-PFL",
         "Compartment"         -> "c",
         "Name"                -> "enzyme of PFL",
         "Link"                -> {"EcoCyc" -> "http://biocyc.org/ECOLI/NEW-IMAGE?type=ENZYME&object=PYRUVFORMLY-CPLX"},
         "Comment"             -> {"TdcE is neglected."},
         "Capacity"            -> 1,
         "Potential"           -> P["E-PFL_IC", "Value"->0.21763935268425891323*^+00001],
         "Scale"               -> 1],
Flux["E-PFL-syn",
     "Reactants"               -> 0 ,
     "Products"                -> "E-PFL",
     "Name"                    -> "enzyme synthesis of PFL",
     "Compartment"             -> "c",
     "Flux"                    -> s[P["PFL_FNR","Value"-> 1],c["FNR"][t]]*s[P["PFL_ArcA","Value"-> 0.2],c["ArcA"][t]],
     "Clamped"                 -> True],
Flux["ADHEr",
     "Reactants"               -> "accoa" + (2) "h" + (2) "nadh",
     "Products"                -> "coa" + "etoh" + (2) "nad",
     "Name"                    -> "Acetaldehyde dehydrogenase",
     "Subsystem"               -> "Pyruvate metabolism",
     "EC"                      -> "1.2.1.10",
     "Compartment"             ->  "c",
     "Link"                    -> {"EcoCyc" -> "http://biocyc.org/ECOLI/NEW-IMAGE?type=REACTION&object=ACETALD-DEHYDROG-RXN"},
     "Resistance"              -> P["ADHEr_k","Value"-> 1.0*^-11]/c["E-ADHEr"][t]/(Cap["accoa"]*Cap["nadh"]^2*Cap["h"]^2)],
Compound["E-ADHEr",
         "Compartment"         -> "c",
```

```
          "Name"                -> "enzyme of ADHEr",
          "Link"                -> {"EcoCyc" -> "http://biocyc.org/ECOLI/NEW-IMAGE?type=ENZYME&object=ADHE-CPLX" ,
                                     "EcoCyc" -> "http://biocyc.org/ECOLI/NEW-IMAGE?type=ENZYME&object=MHPF-MONOMER"},
          "Comment"             -> {"MhpF is neglected."},
          "Capacity"            -> 1,
          "Potential"           -> P["E-ADHEr_IC", "Value"->0.25000196640781822133*^+00001],
          "Scale"               -> 1],
Flux["E-ADHEr-syn",
     "Reactants"                -> 0 ,
     "Products"                 -> "E-ADHEr",
     "Name"                     -> "enzyme synthesis",
     "Compartment"              -> "c",
     "Flux"                     -> s[P["ADHEr_FNR","Value"-> 1],c["FNR"][t]],
     "Clamped"                  -> True],
Flux["FDH-H",
     "Reactants"                -> "for" + "h",
     "Products"                 -> "co2" + "h2",
     "Name"                     -> "Formate Dehydrogenase H",
     "Subsystem"                -> "Oxidative Phosphorylation",
     "EC"                       -> "",
     "Compartment"              ->  "cm",
     "Link"                     -> {"EcoCyc" -> "http://www.ecocyc.org/ECOLI/NEW-IMAGE?type=REACTION&object=FHLMULTI-RXN"},
     "Resistance"               -> P["FDH-H_k","Value"-> 1*^-11]/c["E-FDH-H"][t]/(Cap["for"]*Cap["h"])],
Compound["E-FDH-H",
          "Compartment"         -> "cm",
          "Name"                -> "enzyme of FDH-H",
          "Link"                -> {"EcoCyc" -> "http://www.ecocyc.org/ECOLI/NEW-IMAGE?type=ENZYME&object=FHLMULTI-CPLX"},
          "Capacity"            -> 1,
          "Potential"           -> P["E-FDH-H_IC", "Value"->0.27916886268975051877*^+00001],
          "Scale"               -> 1],
Flux["E-FDH-H-syn",
     "Reactants"                -> 0 ,
     "Products"                 -> "E-FDH-H",
     "Name"                     -> "enzyme synthesis",
     "Compartment"              -> "c",
     "Flux"                     -> s[P["FDH-H_FNR","Value"-> 1],c["FNR"][t]],
     "Clamped"                  -> True],
Flux["FDH-N",
     "Reactants"                -> "for" + (3) "h" + "mqn8",
     "Products"                 -> "co2" + (2) "h(e)" + "mql8",
     "Name"                     -> "Formate Dehydrogenase N",
     "Subsystem"                -> "Oxidative Phosphorylation",
     "EC"                       -> "1.1.5.6",
     "Compartment"              ->  "cm",
     "Link"                     -> {"EcoCyc" -> "http://www.ecocyc.org/ECOLI/NEW-IMAGE?type=REACTION&object=FORMATEDEHYDROG-RXN"},
     "Resistance"               -> P["FDH-N_k","Value"-> 1*^-4]*(c["mql8"][t]+c["mqn8"][t])/c["E-FDH-N"][t]/(Cap["for"]*Cap["mqn8"]*Cap["h"]^3)]
,
Compound["E-FDH-N",
          "Compartment"         -> "cm",
          "Name"                -> "enzyme of FDH-N",
          "Link"                -> {"EcoCyc" -> "http://www.ecocyc.org/ECOLI/NEW-IMAGE?type=ENZYME&object=FORMATEDEHYDROGN-CPLX"},
          "Capacity"            -> 1,
          "Potential"           -> P["E-FDH-N_IC", "Value"->0.27916886268975051877*^+00001],
          "Scale"               -> 1],
Flux["E-FDH-N-syn",
     "Reactants"                -> 0 ,
     "Products"                 -> "E-FDH-N",
     "Name"                     -> "enzyme synthesis",
     "Compartment"              -> "c",
     "Flux"                     -> s[P["FDH-N_FNR","Value"-> 1],c["FNR"][t]],
     "Clamped"                  -> True],
Flux["FDH-O",
     "Reactants"                -> "for" + (3) "h" + "mqn8",
```

```
        "Products"              -> "co2" + (2) "h(e)" + "mql8",
        "Name"                  -> "Formate Dehydrogenase",
        "Subsystem"             -> "Oxidative Phosphorylation",
        "EC"                    -> "1.1.5.6",
        "Compartment"           ->  "cm",
        "Link"                  -> {"EcoCyc" -> "http://www.ecocyc.org/ECOLI/NEW-IMAGE?type=REACTION&object=FORMATEDEHYDROG-RXN"},
        "Resistance"            -> P["FDH-O_k","Value"-> 1*^-4]*(c["mql8"][t]+c["mqn8"][t])/c["E-FDH-O"][t]/(Cap["for"]*Cap["mqn8"]*Cap["h"]^3)]
,
    Compound["E-FDH-O",
            "Compartment"       -> "cm",
            "Name"              -> "enzyme of FDH-O",
            "Link"              -> {"EcoCyc" -> "http://www.ecocyc.org/ECOLI/NEW-IMAGE?type=ENZYME&object=FORMATEDEHYDROGO-CPLX"},
            "Capacity"          -> 1,
            "Potential"         -> P["E-FDH-O_IC", "Value"->0.558330818605416O2381*^+00001],
            "Scale"             -> 1],
    Flux["E-FDH-O-syn",
        "Reactants"             -> 0 ,
        "Products"              -> "E-FDH-O",
        "Name"                  -> "enzyme synthesis",
        "Compartment"           -> "c",
        "Flux"                  -> 1,
        "Clamped"               -> True],

    (* *** Pentose Phosphate Cycle (strongly simplified)                        *** *)
    Flux["G6PDH2r",
        "Reactants"             -> "g6p" + "nadp",
        "Products"              -> "6pgl" + "h" + "nadph",
        "Name"                  -> "glucose 6-phosphate dehydrogenase",
        "Subsystem"             -> "Pentose Phosphate Cycle",
        "EC"                    -> "1.1.1.49",
        "Link"                  -> {"EcoCyc" -> "http://biocyc.org/ECOLI/NEW-IMAGE?type=REACTION&object=GLU6PDEHYDROG-RXN"},
        "Compartment"           -> "c",
        "Resistance"            -> P["G6PDH2r_k","Value"-> 1*^-7]/(Cap["g6p"]*Cap["nadp"])/c["E-G6PDH2r"][t]],
    Compound["E-G6PDH2r",
            "Compartment"       -> "c",
            "Name"              -> "enzyme of G6PDH2r",
            "Capacity"          -> 1,
            "Link"              -> {"EcoCyc"->"http://biocyc.org/ECOLI/NEW-IMAGE?type=ENZYME&object=GLU6PDEHYDROG-MONOMER"},
            "Potential"         -> P["E-G6PDH2r_IC", "Value"->0.14398625135581835099*^+00001],
            "Scale"             -> 1],
    Flux["E-G6PDH2r-syn",
        "Reactants"             -> 0 ,
        "Products"              -> "E-G6PDH2r",
        "Name"                  -> "enzyme synthesis",
        "Compartment"           -> "c",
        "Flux"                  -> s[P["G6PDH2r_FruR","Value"-> 3],1-c["FruR"][t]],
        "Clamped"               -> True],
    Flux["PGL",
        "Reactants"             -> "6pgl" + "h2o",
        "Products"              -> "6pgc" + "h",
        "Name"                  -> "6-phosphogluconolactonase",
        "Subsystem"             -> "Pentose Phosphate Cycle",
        "EC"                    -> "3.1.1.31",
        "Link"                  -> {"EcoCyc" -> "http://biocyc.org/ECOLI/NEW-IMAGE?type=REACTION&object=6PGLUCONOLACT-RXN"},
        "Compartment"           -> "c",
        "Resistance"            -> 0],
    Compound["E-PGL",
            "Compartment"       -> "c",
            "Name"              -> "enzyme of PGL",
            "Capacity"          -> 1,
            "Link"              -> {"EcoCyc"->"http://biocyc.org/ECOLI/NEW-IMAGE?type=ENZYME&object=6PGLUCONOLACT-MONOMER"},
            "Potential"         -> P["E-PGL_IC", "Value"->0.49999774764485973577*^+00001],
            "Scale"             -> 1],
```

```
Flux["E-PGL-syn",
     "Reactants"               -> 0 ,
     "Products"                -> "E-PGL",
     "Name"                    -> "enzyme synthesis",
     "Compartment"             -> "c",
     "Flux"                    -> 1,
     "Clamped"                 -> True],
Flux["GND",
     "Reactants"            -> "6pgc" + "nadp",
     "Products"     ->  "co2" + "nadph" + "ru5p-D",
     "Name"                 -> "phosphogluconate dehydrogenase",
     "Subsystem"            -> "Pentose Phosphate Cycle",
     "EC"           -> "1.1.1.44",
     "Link"                 -> {"EcoCyc" -> "http://biocyc.org/ECOLI/NEW-IMAGE?type=REACTION&object=6PGLUCONDEHYDROG-RXN"},
     "Resistance"           -> P["GND_k","Value"-> 1.0*^-9]/(Cap["6pgc"]*Cap["nadp"])/c["E-GND"][t],
     "Compartment"          ->  "c"],
Compound["E-GND",
         "Compartment"         -> "c",
         "Name"                -> "enzyme of GND",
         "Capacity"            -> 1,
         "Link"                -> {"EcoCyc"->"http://biocyc.org/ECOLI/NEW-IMAGE?type=ENZYME&object=6PGLUCONDEHYDROG-CPLX"},
         "Potential"           -> P["E-GND_IC", "Value"->0.49999774764485973577*^+00001],
         "Scale"               -> 1],
Flux["E-GND-syn",
     "Reactants"               -> 0 ,
     "Products"                -> "E-GND",
     "Name"                    -> "enzyme synthesis",
     "Compartment"             -> "c",
     "Flux"                    -> s[P["GND_FruR","Value"-> 0],1-c["FruR"][t]],
     "Clamped"                 -> True],
Flux["RPE",
     "Reactants"            -> "ru5p-D",
     "Products"     ->  "xu5p-D",
     "Name"                 -> "ribulose 5-phosphate 3-epimerase",
     "Subsystem"            -> "Pentose Phosphate Cycle",
     "EC"           -> "5.1.3.1",
     "Link"                 -> {"EcoCyc" -> "http://biocyc.org/ECOLI/NEW-IMAGE?type=REACTION&object=RIBULP3EPIM-RXN"},
     "Resistance"           -> 0,
     "Compartment"          ->  "c"],
Compound["E-RPE",
         "Compartment"         -> "c",
         "Name"                -> "enzyme of RPE",
         "Capacity"            -> 1,
         "Link"                -> {"EcoCyc"->"http://biocyc.org/ECOLI/NEW-IMAGE?type=ENZYME&object=RIBULP3EPIM-MONOMER"},
         "Potential"           -> P["E-RPE_IC", "Value"->0.49999774764485973577*^+00001],
         "Scale"               -> 1],
Flux["E-RPE-syn",
     "Reactants"               -> 0 ,
     "Products"                -> "E-RPE",
     "Name"                    -> "enzyme synthesis",
     "Compartment"             -> "c",
     "Flux"                    -> 1,
     "Clamped"                 -> True],
Flux["RPI",
     "Reactants"               -> "r5p",
     "Products"                -> "ru5p-D",
     "Name"                    -> "ribose-5-phosphate isomerase",
     "Subsystem"               -> "Pentose Phosphate Cycle",
     "EC"                      -> "5.3.1.6",
     "Link"                    -> {"EcoCyc" -> "http://biocyc.org/ECOLI/NEW-IMAGE?type=REACTION&object=RIB5PISOM-RXN"},
     "Resistance"              -> 0,
     "Compartment"             -> "c"],
Compound["E-RPI",
```

```
        "Compartment"         -> "c",
        "Name"                -> "enzyme of RPI",
        "Capacity"            -> 1,
        "Link"                -> {"EcoCyc"->"http://biocyc.org/ECOLI/NEW-IMAGE?type=ENZYME&object=RIB5PISOMA-CPLX",
                                  "EcoCyc"->"http://biocyc.org/ECOLI/NEW-IMAGE?type=ENZYME&object=RIB5PISOMB-CPLX"},
        "Potential"           -> P["E-RPI_IC", "Value"->0.49999774764485973577*^+00001],
        "Scale"               -> 1],
Flux["E-RPI-syn",
    "Reactants"               -> 0 ,
    "Products"                -> "E-RPI",
    "Name"                    -> "enzyme synthesis",
    "Compartment"             -> "c",
    "Flux"                    -> 1,
    "Clamped"                 -> True],
Flux["TALA",
    "Reactants"               -> "g3p" + "s7p",
    "Products"                ->  "e4p" + "f6p",
    "Name"                    -> "transaldolase",
    "Subsystem"               -> "Pentose Phosphate Cycle",
    "EC"                      -> "2.2.1.2",
    "Link"                    -> {"EcoCyc" -> "http://biocyc.org/ECOLI/NEW-IMAGE?type=REACTION&object=TRANSALDOL-RXN"},
    "Resistance"              -> 0,
    "Compartment"             -> "c"],
Compound["E-TALA",
        "Compartment"         -> "c",
        "Name"                -> "enzyme of TALA",
        "Capacity"            -> 1,
        "Link"                -> {"EcoCyc"->"http://biocyc.org/ECOLI/NEW-IMAGE?type=ENZYME&object=TRANSALDOLA-MONOMER",
                                  "EcoCyc"->"http://biocyc.org/ECOLI/NEW-IMAGE?type=ENZYME&object=TRANSALDOLB-CPLX"},
        "Potential"           -> P["E-TALA_IC", "Value"->0.49999774764485973577*^+00001],
        "Scale"               -> 1],
Flux["E-TALA-syn",
    "Reactants"               -> 0 ,
    "Products"                -> "E-TALA",
    "Name"                    -> "enzyme synthesis",
    "Compartment"             -> "c",
    "Flux"                    -> 1,
    "Clamped"                 -> True],
Flux["TKT1",
    "Reactants"               -> "r5p" + "xu5p-D",
    "Products"                ->  "g3p" + "s7p",
    "Name"                    -> "transketolase",
    "Subsystem"               -> "Pentose Phosphate Cycle",
    "EC"                      -> "2.2.1.1",
    "Link"                    -> {"EcoCyc" -> "http://biocyc.org/ECOLI/NEW-IMAGE?type=REACTION&object=1TRANSKETO-RXN"},
    "Resistance"              -> 0,
    "Compartment"             ->  "c"],
Compound["E-TKT1",
        "Compartment"         -> "c",
        "Name"                -> "enzyme of TKT1",
        "Capacity"            -> 1,
        "Link"                -> {"EcoCyc"->"http://biocyc.org/ECOLI/NEW-IMAGE?type=ENZYME&object=TRANSKETOI-CPLX",
                                  "EcoCyc"->"http://biocyc.org/ECOLI/NEW-IMAGE?type=ENZYME&object=CPLX0-1261"},
        "Potential"           -> P["E-TKT1_IC", "Value"->0.49999774764485973577*^+00001],
        "Scale"               -> 1],
Flux["E-TKT1-syn",
    "Reactants"               -> 0 ,
    "Products"                -> "E-TKT1",
    "Name"                    -> "enzyme synthesis",
    "Compartment"             -> "c",
    "Flux"                    -> 1,
    "Clamped"                 -> True],
```

```
        Flux["TKT2",
             "Reactants"              -> "e4p" + "xu5p-D",
             "Products"               ->  "f6p" + "g3p",
             "Name"                   -> "transketolase",
             "Subsystem"              -> "Pentose Phosphate Cycle",
             "EC"                     -> "2.2.1.1",
             "Link"                   -> {"EcoCyc" -> "http://biocyc.org/ECOLI/NEW-IMAGE?type=REACTION&object=2TRANSKETO-RXN"},
             "Resistance"             -> 0,
             "Compartment"            -> "c"],
        Compound["E-TKT2",
                 "Compartment"        -> "c",
                 "Name"               -> "enzyme of TKT2",
                 "Capacity"           -> 1,
                 "Link"               -> {"EcoCyc"->"http://biocyc.org/ECOLI/NEW-IMAGE?type=ENZYME&object=TRANSKETOI-CPLX",
                                           "EcoCyc"->"http://biocyc.org/ECOLI/NEW-IMAGE?type=ENZYME&object=CPLX0-1261"},
                 "Potential"          -> P["E-TKT2_IC", "Value"->0.49999774764485973577*^+00001],
                 "Scale"              -> 1],
        Flux["E-TKT2-syn",
             "Reactants"              -> 0 ,
             "Products"               -> "E-TKT2",
             "Name"                   -> "enzyme synthesis",
             "Compartment"            -> "c",
             "Flux"                   -> 1,
             "Clamped"                -> True],

        (* *** Oxidative Phosphorylation                                              *** *)
        Flux["NADHI(q8)",
             "Reactants"              -> "h" + "nadh" + "q8" + 4 "h",
             "Products"               -> "nad" + "q8h2" + 4 "h(p+)",
             "Name"                   -> "NADH dehydrogenase (ubiquinone-8 ) nuo",
             "Subsystem"              -> "Oxidative phosphorylation",
             "EC"                     -> "1.6.5.3",
             "Link"                   -> {"EcoCyc"->"http://www.ecocyc.org/ECOLI/NEW-IMAGE?type=REACTION&object=NADH-DEHYDROG-A-RXN"},
             "Compartment"            -> "cm",
             "Resistance"             -> P["NADHI(q8)_k","Value"->     1.0*^-9]*(c["q8h2"][t]+c["q8"][t])/(Cap["h"]^5*Cap["nadh"]*Cap["q8"])/c["E-NADH
I"][t]],
        Flux["NADHI(mqn8)",
             "Reactants"              -> "h" + "nadh" + "mqn8" + 4 "h",
             "Products"               -> "nad" + "mql8" + 4 "h(p+)",
             "Name"                   -> "NADH dehydrogenase (menaquinone-8 ) nuo",
             "Subsystem"              -> "Oxidative phosphorylation",
             "EC"                     -> "1.6.5.-",
             "Link"                   -> {"EcoCyc"->"http://www.ecocyc.org/ECOLI/NEW-IMAGE?type=REACTION&object=RXN0-5388"},
             "Compartment"            -> "cm",
             "Resistance"             -> (P["NADHI(mqn8)_k","Value"->   1.0*^-14]*(c["mql8"][t]+c["mqn8"][t])/
                                          (Cap["h"]^5*Cap["nadh"]*Cap["mqn8"])/c["E-NADHI"][t])],
        Compound["E-NADHI",
                 "Compartment"        -> "cm",
                 "Name"               -> "enzyme of NADH-DH I",
                 "Link"               -> {"EcoCyc" -> "http://www.ecocyc.org/ECOLI/NEW-IMAGE?type=ENZYME&object=NADH-DHI-CPLX"},
                 "Capacity"           -> 1,
                 "Potential"          -> P["E-NADHI_IC", "Value"->0.55832563847867682227*^+00001],
                 "Scale"              -> 1],
        Flux["E-NADHI-syn",
             "Reactants"              -> 0 ,
             "Products"               -> "E-NADHI",
             "Name"                   -> "enzyme synthesis",
             "Compartment"            -> "c",
             "Flux"                   -> s[P["NADHI_FNR","Value"-> 1],1-c["FNR"][t]]*s[P["NADHI_ArcA","Value"-> 1],1-c["ArcA"][t]],
             "Clamped"                -> True],
        Flux["NADHII(q8)",
             "Reactants"              -> "h" + "nadh" + "q8",
             "Products"               -> "nad" + "q8h2",
```

```
                "Name"                   -> "NADH dehydrogenase (ubiquinone-8 ) ndh",
                "Subsystem"              -> "Oxidative phosphorylation",
                "EC"                     -> "1.6.5.9",
                "Link"                   -> {"EcoCyc"->"http://www.ecocyc.org/ECOLI/NEW-IMAGE?type=REACTION&object=RXN0-5330"},
                "Compartment"            -> "cm",
                "Resistance"             -> P["NADHII_k","Value"->          1.0*^-4]*(c["q8h2"][t]+c["q8"][t])/(Cap["h"]*Cap["nadh"]*Cap["q8"])/c["E-NADH
II"][t]],
           Compound["E-NADHII",
                    "Compartment"        -> "cm",
                    "Name"               -> "enzyme of NADH-DH II",
                    "Link"               -> {"EcoCyc"->"http://www.ecocyc.org/ECOLI/NEW-IMAGE?type=GENE&object=EG10649"},
                    "Capacity"           -> 1,
                    "Potential"          -> P["E-NADHII_IC", "Value"->0.69916794370916646173*^+00000],
                    "Scale"              -> 1],
           Flux["E-NADHII-syn",
                "Reactants"              -> 0 ,
                "Products"               -> "E-NADHII",
                "Name"                   -> "enzyme synthesis",
                "Compartment"            -> "c",
                "Flux"                   -> (s[P["NADHII_FNR","Value"-> 1],1-c["FNR"][t]]*s[P["NADHII_ArcA","Value"-> 1],c["ArcA"][t]]*
                                             s[P["NADHII_PdhR","Value"-> 1],1-c["PdhR"][t]]),
                "Clamped"                -> True],
           Flux["CYTBD2",
                "Reactants"              -> 2*P["BD2_H","Value"-> 2.0] "h" + "o2" + 2 "q8h2",
                "Products"               -> 2*P["BD2_H"] "h(p+)"+  2 * "h2o" + 2* "q8",
                "Name"                   -> "cytochrome oxidase bd2",
                "Subsystem"              -> "Oxidative phosphorylation",
                "EC"                     -> "1.10.3.-",
                "Link"                   -> {"EcoCyc"->"http://www.ecocyc.org/ECOLI/NEW-IMAGE?type=REACTION&object=RXN0-5266"},
                "Compartment"            -> "cm",
                "Resistance"             -> (P["CYTBD2_k","Value"->    1.0*^-11]*(c["q8h2"][t]+c["q8"][t])^2/
                                             c["E-CYTBD2"][t]/(Cap["h"]^P["BD2_H"]*Cap["o2"]*Cap["q8h2"]^2))],
           Compound["E-CYTBD2",
                    "Compartment"        -> "cm",
                    "Name"               -> "enzyme of bd2",
                    "Link"               -> {"EcoCyc"->"http://www.ecocyc.org/ECOLI/NEW-IMAGE?type=ENZYME&object=APP-UBIOX-CPLX"},
                    "Capacity"           -> 1,
                    "Potential"          -> P["E-CYTBD2_IC", "Value"->0.89920486398578039489*^-00001],
                    "Scale"              -> 1],
           Flux["E-CYTBD2-syn",
                "Reactants"              -> 0 ,
                "Products"               -> "E-CYTBD2",
                "Name"                   -> "enzyme synthesis",
                "Compartment"            -> "c",
                "Flux"                   -> (P["CYTBD2_E","Value"->1,"TeXExport"->False]*s[P["CYTBD2_ArcA","Value"-> 5],c["ArcA"][t]]*
                                             s[P["CYTBD2_AppY","Value"-> 5],c["AppY"][t]]),
                "Clamped"                -> True],
           Flux["CYTBD",
                "Reactants"              -> 2*P["BD_H","Value"-> 2.0] "h" + (1) "o2" + 2 "q8h2",
                "Products"               -> 2*P["BD_H"] "h(p+)" + 2* "h2o" + 2* "q8",
                "Name"                   -> "cytochrome oxidase bd",
                "Subsystem"              -> "Oxidative phosphorylation",
                "EC"                     -> "1.10.3.-",
                "Link"                   -> {"EcoCyc"->"http://www.ecocyc.org/ECOLI/NEW-IMAGE?type=REACTION&object=RXN0-5266"},
                "Compartment"            -> "cm",
                "Resistance"             -> (P["CYTBD_k","Value"->     1.0*^-12]*(c["q8h2"][t]+c["q8"][t])^2/
                                             c["E-CYTBD"][t]/(Cap["h"]^P["BD_H"]*Cap["o2"]*Cap["q8h2"]^2))],
           Compound["E-CYTBD",
                    "Compartment"        -> "cm",
                    "Name"               -> "enzyme of bd",
                    "Link"               -> {"EcoCyc"->"http://www.ecocyc.org/ECOLI/NEW-IMAGE?type=ENZYME&object=CYT-D-UBIOX-CPLX"},
                    "Capacity"           -> 1,
                    "Potential"          -> P["E-CYTBD_IC", "Value"->0.17447649271260848303*^+00000],
```

```
         "Scale"                  -> 1],
Flux["E-CYTBD-syn",
     "Reactants"                  -> 0 ,
     "Products"                   -> "E-CYTBD",
     "Name"                       -> "enzyme synthesis",
     "Compartment"                -> "c",
     "Flux"                       -> (P["CYTBD_E","Value"->1,"TeXExport"->False]*s[P["CYTBD_FNR","Value"->5],1-c["FNR"][t]]*
                                      s[P["CYTBD_ArcA","Value"-> 5],c["ArcA"][t]]*s[P["CYTBD_FruR","Value"-> 1],c["FruR"][t]]),
     "Clamped"                    -> True],
Flux["CYTBO3",
     "Reactants"                  -> 2*P["BO_H","Value"-> 4] "h" + (1) "o2" + 2 "q8h2",
     "Products"                   -> 2*P["BO_H"] "h(p+)" + 2* "h2o" + 2* "q8",
     "Name"                       -> "cytochrome oxidase bo",
     "Subsystem"                  -> "Oxidative phosphorylation",
     "EC"                         -> "1.10.3.10",
     "Link"                       -> {"EcoCyc"->"http://www.ecocyc.org/ECOLI/NEW-IMAGE?type=REACTION&object=RXN0-5268"},
     "Compartment"                -> "cm",
     "Resistance"                 -> (P["CYTBO3_k","Value"->    1.0*^-16]*(c["q8h2"][t]+c["q8"][t])^2/c["E-CYTBO3"][t]/
                                      (Cap["h"]^P["BO_H"]*Cap["o2"]*Cap["q8h2"]^2))],
Compound["E-CYTBO3",
         "Compartment"            -> "cm",
         "Name"                   -> "enzyme of bo",
         "Link"                   -> {"EcoCyc"->"http://www.ecocyc.org/ECOLI/NEW-IMAGE?type=ENZYME&object=CYT-O-UBIOX-CPLX"},
         "Capacity"               -> 1,
         "Potential"              -> P["E-CYTBO3_IC", "Value"->0.118597353357753787 51*^+00000],
         "Scale"                  -> 1],
Flux["E-CYTBO3-syn",
     "Reactants"                  -> 0 ,
     "Products"                   -> "E-CYTBO3",
     "Name"                       -> "enzyme synthesis",
     "Compartment"                -> "c",
     "Flux"                       -> (P["CYTBO3_E","Value"->1,"TeXExport"->False]*s[P["CYTBO_FNR","Value"-> 3],1-c["FNR"][t]]*
                                      s[P["CYTBO_ArcA","Value"-> 3],1-c["ArcA"][t]]*s[P["CYTBO_PdhR","Value"-> 3],1-c["PdhR"][t]]*
                                      s[P["CYTBO_CRP","Value"-> 3],c["CRP"][t]]*s[P["CYTBO_FruR","Value"-> 3],1-c["FruR"][t]]),
     "Clamped"                    -> True],
Flux["ATPS",
     "Reactants"                  -> "adp" + 40/10 "h(p+)" + "pi",
     "Products"                   -> "atp" + 3 "h" + "h2o",
     "Name"                       -> "ATP synthase (four protons for one ATP)",
     "Subsystem"                  -> "Oxidative phosphorylation",
     "EC"                         -> "3.6.3.14",
     "Link"                       -> {"EcoCyc" -> "http://www.ecocyc.org/ECOLI/NEW-IMAGE?type=REACTION&object=ATPSYN-RXN"},
     "Compartment"                -> "cm",
     "Resistance"                 -> 0/c["E-ATPS"][t]],
Compound["E-ATPS",
         "Compartment"            -> "cm",
         "Name"                   -> "enzyme of ATPS",
         "Link"                   -> {"EcoCyc" -> "http://www.ecocyc.org/ECOLI/NEW-IMAGE?type=ENZYME&object=ATPSYN-CPLX"},
         "Capacity"               -> 1,
         "Potential"              -> P["E-ATPS_IC", "Value"->0.55833081860541602381*^+00001],
         "Scale"                  -> 1],
Flux["E-ATPS-syn",
     "Reactants"                  -> 0,
     "Products"                   -> "E-ATPS",
     "Name"                       -> "enzyme synthesis of ATPS",
     "Compartment"                -> "c",
     "Flux"                       -> 1,
     "Clamped"                    -> True],
Flux["ADK",
     "Reactants"                  -> "amp" + "atp",
     "Products"                   -> (2) "adp",
     "Name"                       -> "adenylate kinase",
     "Subsystem"                  -> "Nucleotide Salvage Pathways",
```

```
     "EC"                      -> "2.7.4.3",
     "Link"                    -> {"EcoCyc" -> "http://www.ecocyc.org/ECOLI/NEW-IMAGE?type=REACTION&object=ADENYL-KIN-RXN"},
     "Compartment"             -> "c",
     "Resistance"              -> 0],
Compound["E-ADK",
         "Compartment"         -> "c",
         "Name"                -> "enzyme of ADK",
         "Link"                -> {"EcoCyc" -> "http://www.ecocyc.org/ECOLI/NEW-IMAGE?type=ENZYME&object=ADENYL-KIN-MONOMER"},
         "Capacity"            -> 1,
         "Potential"           -> P["E-ADK_IC", "Value"->0.49999774764485973577*^+00001],
         "Scale"               -> 1],
Flux["E-ADK-syn",
     "Reactants"               -> 0,
     "Products"                -> "E-ADK",
     "Name"                    -> "enzyme synthesis of ADK",
     "Compartment"             -> "c",
     "Flux"                    -> 1,
     "Clamped"                 -> True],
Flux["ATPM",
     "Reactants"               -> "atp" + "h2o",
     "Products"                -> "adp" + "h" + "pi",
     "Name"                    -> "ATP maintenance requirement",
     "Subsystem"               -> "Oxidative Phosphorylation",
     "Compartment"             -> "c",
     "Clamped"                 -> True,
     "Flux"                    -> P["ATPM_TH","Value"-> 10]*TH[c["atp"][t]/c["adp"][t],P["ATPM_TH_LO","Value"-> 2.5],P["ATPM_TH_HI","Value"-> 3
.0]]],
Flux["PPA",
     "Reactants"               -> "ppi"  + "h2o",
     "Products"                -> 2 "pi"  + "h",
     "Name"                    -> "inorganic pyrophosphatase",
     "Link"                    -> {"EcoCyc" -> "http://biocyc.org/ECOLI/NEW-IMAGE?type=REACTION&object=INORGPYROPHOSPHAT-RXN"},
     "EC"                      ->  "3.6.1.1",
     "Compartment"             -> "c",
     "Resistance"              -> 0],
Compound["E-PPA",
         "Compartment"         -> "c",
         "Name"                -> "enzyme of PPA",
         "Link"                -> {"EcoCyc" -> "http://www.ecocyc.org/ECOLI/NEW-IMAGE?type=ENZYME&object=CPLX0-243"},
         "Capacity"            -> 1,
         "Potential"           -> P["E-PPA_IC", "Value"->0.49999774764485973577*^+00001],
         "Scale"               -> 1],
Flux["E-PPA-syn",
     "Reactants"               -> 0,
     "Products"                -> "E-PPA",
     "Name"                    -> "enzyme synthesis of PPA",
     "Compartment"             -> "c",
     "Flux"                    -> 1,
     "Clamped"                 -> True],
Flux["THD-SthA",
     "Reactants"               -> "nad" + "nadph",
     "Products"                -> "nadh" + "nadp",
     "Name"                    -> "NADH transhydrogenase",
     "Subsystem"               -> "Oxidative Phosphorylation",
     "EC"                      -> "1.6.1.1",
     "Link"                    -> {"EcoCyc" -> "http://www.ecocyc.org/ECOLI/NEW-IMAGE?type=REACTION&object=PYRNUTRANSHYDROGEN-RXN"},
     "Compartment"             -> "c",
     "Resistance"              -> P["THD-SthA_k","Value"-> 1.0*^-5]/(Cap["nad"]*Cap["nadph"])/c["E-THD-SthA"][t]],
Compound["E-THD-SthA",
         "Compartment"         -> "c",
         "Name"                -> "enzyme of THD-SthA",
         "Link"                -> {"EcoCyc" -> "http://www.ecocyc.org/ECOLI/NEW-IMAGE?type=ENZYME&object=UDHA-CPLX"},
         "Capacity"            -> 1,
```

```
         "Potential"           -> P["E-THD-SthA_IC", "Value"->0.49999774764485973577*^+00001],
         "Scale"               -> 1],
Flux["E-THD-SthA-syn",
     "Reactants"               -> 0,
     "Products"                -> "E-THD-SthA",
     "Name"                    -> "enzyme synthesis of THD-SthA",
     "Compartment"             -> "c",
     "Flux"                    -> 1,
     "Clamped"                 -> True],
Flux["THD-PntAB",
     "Reactants"               -> 2 "h" + "nad" + "nadph",
     "Products"                -> 2 "h(e)" + "nadh" + "nadp",
     "Name"                    -> "NADH transhydrogenase",
     "Subsystem"               -> "Oxidative Phosphorylation",
     "EC"                      -> "1.6.1.2",
     "Link"                    -> {"EcoCyc" -> "http://www.ecocyc.org/ECOLI/NEW-IMAGE?type=REACTION&object=TRANS-RXN0-277"},
     "Compartment"             -> "cm",
     "Resistance"              -> P["THD-PntAB_k","Value"-> 1.0*^-1]/(Cap["nad"]*Cap["nadph"])/c["E-THD-PntAB"][t]],
Compound["E-THD-PntAB",
         "Compartment"         -> "cm",
         "Name"                -> "enzyme of THD-PntAB",
         "Link"                -> {"EcoCyc" -> "http://www.ecocyc.org/ECOLI/NEW-IMAGE?type=ENZYME&object=PYRNUTRANSHYDROGEN-CPLX"},
         "Capacity"            -> 1,
         "Potential"           -> P["E-THD-PntAB_IC", "Value"->0.55833081860541602381*^+00001],
         "Scale"               -> 1],
Flux["E-THD-PntAB-syn",
     "Reactants"               -> 0,
     "Products"                -> "E-THD-PntAB",
     "Name"                    -> "enzyme synthesis of THD-PntAB",
     "Compartment"             -> "c",
     "Flux"                    -> 1,
     "Clamped"                 -> True],


(* *** growth                                      *** *)

Flux["GROWTH",
     "Reactants"               ->
     Rationalize[Expand[(
         +P["GROWTH_nu_g6p",     "Value" -> 114.878]    * "g6p"
         +P["GROWTH_nu_f6p",     "Value" -> 40.0464]    * "f6p"
         +P["GROWTH_nu_dhap",     "Value" -> 141.76]    * "dhap"
         +P["GROWTH_nu_3pg",     "Value" -> 1782.47]    * "3pg"
         +P["GROWTH_nu_pep",     "Value" -> 867.078]    * "pep"
         +P["GROWTH_nu_pyr",     "Value" -> 3016.96]    * "pyr"
         +P["GROWTH_nu_accoa",   "Value" -> 3610.63]    * "accoa"
         +P["GROWTH_nu_succoa",  "Value" -> 565.8]      * "succoa"
         +P["GROWTH_nu_akg",     "Value" -> 1169.72]    * "akg"
         +P["GROWTH_nu_oaa",     "Value" -> 2865.58]    * "oaa"
         +P["GROWTH_nu_r5p",     "Value" -> 843.385]    * "r5p"
         +P["GROWTH_nu_e4p",     "Value" -> 420.075]    * "e4p"
         +P["GROWTH_nu_atp",        "Value" -> 39609.8] * "atp"
         +P["GROWTH_nu_nadph",   "Value" -> 17542.9]    * "nadph"
         +P["GROWTH_nu_nad", "Value" -> 2993.3]         * "nad"
                        )*(DCW/Vc)*10^(-6)],10^(-100)],
     "Products"                ->
     Rationalize[Expand[(
         +(P["GROWTH_nu_accoa"]+P["GROWTH_nu_succoa"]) * "coa"
         +P["GROWTH_nu_atp"]                           * "adp"
         +P["GROWTH_nu_nad"]                           * "nadh"
         +P["GROWTH_nu_nadph"]                         * "nadp"
         -P["GROWTH_nu_g3p",     "Value" ->  -62.835]   * "g3p"
         -P["GROWTH_nu_succ",     "Value" -> -565.8]    * "succ"
```

```
          -P["GROWTH_nu_fum",     "Value" -> -1088.13]   * "fum"
          -P["GROWTH_nu_co2",     "Value" -> -1876.89]   * "co2"
          -P["GROWTH_nu_ac",     "Value"  -> -598.11]    * "ac"
                         )*(DCW/Vc)*10^(-6)],10^(-100)],
      "Name"                    -> "Growth",
      "Subsystem"               -> "Growth",
      "Compartment"             -> "c",
      "Clamped"                 -> True,
      "TeXExport"               -> False,
      "Flux"                    ->
      TH[c["atp"][t]/c["adp"][t],P["GROWTH_ATP_LO","Value"-> 2.5],P["GROWTH_ATP_HI","Value"-> 3.0]]
      P["GROWTH_A","Value" -> 4*^-5]*
      (P["GROWTH_B","Value" -> 0.65*^6]                   +
       P["GROWTH_nu_g6p"]    * Log[c["g6p"][t]]    +
       P["GROWTH_nu_f6p"]    * Log[c["f6p"][t]]    +
       P["GROWTH_nu_dhap"]   * Log[c["dhap"][t]]   +
       P["GROWTH_nu_3pg"]    * Log[c["3pg"][t]]    +
       P["GROWTH_nu_pep"]    * Log[c["pep"][t]]    +
       P["GROWTH_nu_pyr"]    * Log[c["pyr"][t]]    +
       P["GROWTH_nu_accoa"]  * Log[c["accoa"][t]]  +
       P["GROWTH_nu_succoa"] * Log[c["succoa"][t]] +
       P["GROWTH_nu_akg"]    * Log[c["akg"][t]]    +
       P["GROWTH_nu_oaa"]    * Log[c["oaa"][t]]    +
       P["GROWTH_nu_r5p"]    * Log[c["r5p"][t]]    +
       P["GROWTH_nu_e4p"]    * Log[c["e4p"][t]]    +
       P["GROWTH_nu_atp"]    * Log[c["atp"][t]]    +
       P["GROWTH_nu_nad"]    * Log[c["nad"][t]]    +
       P["GROWTH_nu_nadph"]  * Log[c["nadph"][t]]),
      "Resistance"              -> Indeterminate],

(* *** genetic regulation                                 *** *)
Compound["PdhR",
         "Name"                 -> "Transcription Factor PdhR (unmodified form)",
         "Comment"              -> "PdhR + pyruvate = PdhR-pyruvate; PdhR is Repressor",
         "Clamped"              -> True,
         "Compartment"          -> "c",
         "Link"                 -> {"EcoCyc"->"http://biocyc.org/ECOLI/NEW-IMAGE?type=ENZYME&object=EG11088-MONOMER"},
         "Capacity"             -> 1,
         "Potential"            -> MM[(c["pyr"][t]/P["PdhR_km","Value"-> 1.0*^-4])^P["PdhR_n","Value"-> -1]]],
Compound["ArcA",
         "Name"                 -> "Transcription Factor ArcA (phosphorylatd form)",
         "Clamped"              -> True,
         "Compartment"          -> "c",
         "Capacity"             -> 1,
         "Link"                 -> {"EcoCyc"->"http://biocyc.org/ECOLI/NEW-IMAGE?type=ENZYME&object=PHOSPHO-ARCA"},
         "Potential"            -> MM[((c["q8"][t]  *P["ArcA_dea_q","Value"->1]+c["mqn8"][t]*(1-P["ArcA_dea_q"]))/
                                    (c["q8h2"][t]*P["ArcA_act_q","Value"->0]+c["mql8"][t]*(1-P["ArcA_act_q"]))/
                                    P["ArcA_km","Value"-> 20.0])^P["ArcA_n_q8","Value"-> -1]]],
Compound["FNR",
         "Name"                 -> "Transcription Factor FNR (reduced form)",
         "Clamped"              -> True,
         "Compartment"          -> "c",
         "Capacity"             -> 1,
         "Link"                 -> {"EcoCyc"->"http://biocyc.org/ECOLI/NEW-IMAGE?type=ENZYME&object=CPLX0-7797"},
         "Potential"            -> MM[(c["o2"][t]/P["FNR_km_o2","Value"-> 0.1*^-6])^P["FNR_n","Value"-> -2]]],
Compound["FruR",
         "Name"                 -> "Transcription Factor FruR",
         "Clamped"              -> True,
         "Compartment"          -> "c",
         "Capacity"             -> 1,
         "Link"                 -> {"EcoCyc"->"http://biocyc.org/ECOLI/NEW-IMAGE?type=ENZYME&object=CPLX-128"},
         "Potential"            -> MM[(c["fdp"][t]/P["FruR_km_fdp","Value"-> 1*^-6])^P["FruR_n","Value"-> -1]]],
Compound["AppY",
```

```
                                "Name"                  -> "Transcription Factor AppY (phosphorylatd form)",
                                "Clamped"               -> True,
                                "Compartment"           -> "c",
                                "Link"                  -> {"EcoCyc"->"http://biocyc.org/ECOLI/NEW-IMAGE?type=ENZYME&object=PD00967"},
                                "Capacity"              -> 1,
                                "Potential"             -> MM[(c["for"][t]/P["AppY_km_for","Value"-> 1*^-5])^P["AppY_n","Value"-> 2]]],
                       Compound["CRP",
                                "Clamped"               -> True,
                                "Name"                  -> "Transcription Factor CRP-cAMP",
                                "Compartment"           -> "c",
                                "Capacity"              -> 1,
                                "Link"                  -> {"EcoCyc"->"http://biocyc.org/ECOLI/NEW-IMAGE?type=ENZYME&object=CPLX0-226"},
                                "Potential"             -> MM[(c["pep"][t]/c["pyr"][t]/P["CRP_km","Value"-> 1.0*^-1])^P["CRP_n","Value"-> 1]]],
                       Compound["IclR",
                                "Name"                  -> "Transcription Factor IcR (active form)",
                                "Clamped"               -> True,
                                "Compartment"           -> "c",
                                "Capacity"              -> 1,
                                "Link"                  -> {"EcoCyc"->"http://biocyc.org/ECOLI/NEW-IMAGE?type=ENZYME&object=PD04099"},
                                "Potential"             -> MM[(c["pyr"][t]/c["glx"][t]/P["IclR_km","Value"-> 10])^P["IclR_n","Value"-> 1]]]
                   },
                   "Tsim" -> P["TOUT","Value"->t1]];

(* *** Adding the production flux                                                *** *)
net = net /.
NetworkDescription[n_,d_,opts___]:>
NetworkDescription[n,
                   Module[{fprod,Cs,cprod},
                          Cs = Cases[d,Compound[c_,___]:>c];
                          fprod=Flux["PROD",
                                     "Reactants"      -> 0,
                                     "Products"       -> Total[((P[("PROD_nu_"<>#),"Value"->0]*#)& /@ Cs)],
                                     "Clamped"        -> True,
                                     "Compartment"    -> "c",
                                     "TeXExport"      -> False,
                                     "Flux"           -> P["PROD_k","Value"->0]*DCW/Vc*10^(-3)*c["kprod"][t]];
                          cprod=Compound["kprod",
                                         "Clamped"    -> True,
                                         "Compartment"-> "c",
                                         "Capacity"   -> 1,
                                         "Potential"  -> TH[t,P["kprod_t0","Value"->t0],P["kprod_t1","Value"->t1]]];
                          Join[d,{cprod,fprod}]],
                   opts];
net]
```
