# Supplementary material for: A mathematical model of metabolism and regulation provides a systems-level view of how Escherichia coli responds to oxygen
Source: Front Microbiol. 2014 Mar 27;5:124. doi: 10.3389/fmicb.2014.00124 (PMC3973912; doi:10.3389/fmicb.2014.00124)
Supplement: Supplementary Data Sheet 3 — Transcription Factors and Their Metabolic Signals. [file DataSheet3.PDF]

Transcription Factors and Their Metabolic Signals

In the model, we use the activity $a_{\mathrm{TF},i}$ of transcription factor i. Minimal transcription factor activity is represented by $a_{\mathrm{TF},i} = 0$, while maximal activity is represented by $a_{\mathrm{TF},i} = 1$.

We assume a phenomenological Hill type equation

$$a_{\mathrm{TF},i} = \frac{x_{\mathrm{TF},i}^{n_{\mathrm{TF},i}}}{x_{\mathrm{TF},i}^{n_{\mathrm{TF},i}} + k_{\mathrm{TF},i}^{n_{\mathrm{TF},i}}}.$$

that describes $a_{\mathrm{TF},i}$ in dependence on the respective metabolic signal $x_{\mathrm{TF},i}$. For $n_{\mathrm{TF},i} > 0$ transcription factor i is activated by its metabolic signal $x_{\mathrm{TF},i}$. For $n_{\mathrm{TF},i} < 0$ it is inhibited. The following table lists the transcription factors together with their metabolic signals x_{TF} used in the model and the according references that motivated the choice of the expression for x_{TF}.

	Metabolic Signal		Parameters	
	x_{TF}	Reference	$k_{TF,i}$	$n_{TF,i}$
FNR	c_{O_2}	[13, 8]	0.1 µmol/l	−2
ArcA[a]	$x_{ArcA}(c_{\mathrm{q8}}, c_{\mathrm{q8h2}}, c_{\mathrm{mqn8}}, c_{\mathrm{mql8}})$	[6, 1]	20	−1
CRP	$c_{\mathrm{pep}}/c_{\mathrm{pyr}}$	[7, 4]	0.1	1
FruR	c_{fdp}	[9]	0.1 µmol/l	−1
PdhR	c_{pyr}	[12, 11]	100 µmol/l	
AppY	c_{for}	[5, 2]	10 µmol/l	2
IclR	$c_{\mathrm{pyr}}/c_{\mathrm{glx}}$	[10]	10	1

a) The precise nature of the metabolic signal of ArcA is subject to a current debate. **Alvarez et al.** [1] report that ubiquinone is necessary to deactivate the sensor kinase ArcB and that menaquinole is necessary for activation of the sensor kinase ArcB. In contrast, **Bekker et al.** [3], **Sharma et al.** [14] report that also other quinone species control the activity of ArcB. To be able to capture the different hypotheses we choose the following expression for the metabolic signal of ArcB

$$x_{ArcA} = \frac{k_{dea,q} \cdot c_{\mathrm{q8}} + (1 - k_{dea,q}) \cdot c_{\mathrm{mqn8}}}{k_{act,q} \cdot c_{\mathrm{q8h2}} + (1 - k_{act,q}) \cdot c_{\mathrm{mql8}}}, \qquad \text{with } 0 \leq k_{dea,q}, k_{act,q} \leq 1.$$

The parameters $k_{dea,q}$ and $k_{act,q}$ determine the relative importance of the ubiquinones over the menaquinones in deactivation and activation of ArcB, respectively. A simulation study with the model (not shown) shows that different choices of the parameters $k_{dea,q}$ and $k_{act,q}$ are able to explain the data that the model uses as a reference. As our model cannot accommodate the influence of the separate redox states of ubiquinol and menaquinol onto ArcA, we have selected to use $k_{dea,q} = 1$ and $k_{act,q} = 0$.

References

[1] Alvarez, A. F., Rodriguez, C., and Georgellis, D. (2013), Ubiquinone and Menaquinone Electron Carriers Represent the Yin and Yang in the Redox Regulation of the ArcB Sensor Kinase., *J. Bacteriol.*, 195, 13, 3054–61, doi:10.1128/JB.00406-13

[2] Atlung, T., Knudsen, K., Heerfordt, L., and Brøndsted, L. (1997), Effects of sigmaS and the transcriptional activator AppY on induction of the *Escherichia coli hya* and *cbdAB-appA* operons in response to carbon and phosphate starvation., *J. Bacteriol.*, 179, 7, 2141–6

[3] Bekker, M., Alexeeva, S., Laan, W., Sawers, G., Teixeira de Mattos, J., and Hellingwerf, K. (2010), The ArcBA two-component system of *Escherichia coli* is regulated by the redox state of both the ubiquinone and the menaquinone pool., *J. Bacteriol.*, 192, 3, 746–54, doi:10.1128/JB.01156-09

[4] Bettenbrock, K., Fischer, S., Kremling, A., Jahreis, K., Sauter, T., and Gilles, E.-D. (2006), A quantitative approach to catabolite repression in Escherichia coli, *J. Biol. Chem.*, 281, 5, 2578–2584, doi: 10.1074/jbc.M508090200

[5] Brøndsted, L. and Atlung, T. (1996), Effect of growth conditions on expression of the acid phosphatase (*cyx-appA*) operon and the *appY* gene, which encodes a transcriptional activator of *Escherichia coli*, *J. Bacteriol.*, 178, 6, 1556–1564

[6] Georgellis, D., Lynch, A., and Lin, E. (1997), In vitro phosphorylation study of the arc two-component signal transduction system of *Escherichia coli*, *J. Bacteriol.*, 179, 17, 5429–5435

[7] Hogema, B. M., Arents, J. C., Bader, R., Eijkemans, K., Yoshida, H., Takahashi, H., Aiba, H., and Postma, P. W. (1998), Inducer exclusion in Escherichia coli by non-PTS substrates: the role of the PEP to pyruvate ratio in determining the phosphorylation state of enzyme IIAGlc, *Molecular Microbiology*, 30, 3, 487–498, doi:10.1046/j.1365-2958.1998.01053.x

[8] Jordan, P. A., Thomson, A. J., Ralph, E. T., Guest, J. R., and Green, J. (1997), FNR is a direct oxygen sensor having a biphasic response curve, *FEBS Lett.*, 416, 3, 349–352

[9] Kochanowski, K., Volkmer, B., Gerosa, L., Haverkorn van Rijsewijk, B. R., Schmidt, A., and Heinemann, M. (2013), Functioning of a metabolic flux sensor in *Escherichia coli.*, *Proc. Natl. Acad. Sci. U.S.A.*, 110, 3, 1130–5, doi:10.1073/pnas.1202582110

[10] Lorca, G. L., Ezersky, A., Lunin, V. V., Walker, J. R., Altamentova, S., Evdokimova, E., Vedadi, M., Bochkarev, A., and Savchenko, A. (2007), Glyoxylate and pyruvate are antagonistic effectors of the Escherichia coli IclR transcriptional regulator., *J. Biol. Chem.*, 282, 22, 16476–91, doi: 10.1074/jbc.M610838200

[11] Ogasawara, H., Ishida, Y., Yamada, K., Yamamoto, K., and Ishihama, A. (2007), PdhR (pyruvate dehydrogenase complex regulator) controls the respiratory electron transport system in Escherichia coli., *J. Bacteriol.*, 189, 15, 5534–41, doi:10.1128/JB.00229-07

[12] Quail, M. A. and Guest, J. R. (1995), Purification, characterization and mode of action of PdhR, the transcriptional repressor of the pdhR-aceEF-lpd operon of Escherichia coli., *Molecular microbiology*, 15, 3, 519–29

[13] Sawers, G. (1999), The aerobic/anaerobic interface, *Curr. Opin. Microbiol.*, 2, 2, 181–187, doi: 10.1016/S1369-5274(99)80032-0

[14] Sharma, P., Stagge, S., Bekker, M., Bettenbrock, K., and Hellingwerf, K. J. (2013), Kinase Activity of ArcB from *Escherichia coli* is Subject to Regulation by Both Ubiquinone and Demethylmenaquinone., *PLoS ONE*, 8, 10, e75412, doi:10.1371/journal.pone.0075412
