# Supplementary material for: A mathematical model of metabolism and regulation provides a systems-level view of how Escherichia coli responds to oxygen
Source: Front Microbiol. 2014 Mar 27;5:124. doi: 10.3389/fmicb.2014.00124 (PMC3973912; doi:10.3389/fmicb.2014.00124)
Supplement: Supplementary Data Sheet 4 — Map of Measurement Data and Model Results. [file DataSheet4.PDF]

Supplementary Data Sheet 4: Model Results – Central Metabolism of *Escherichia coli*

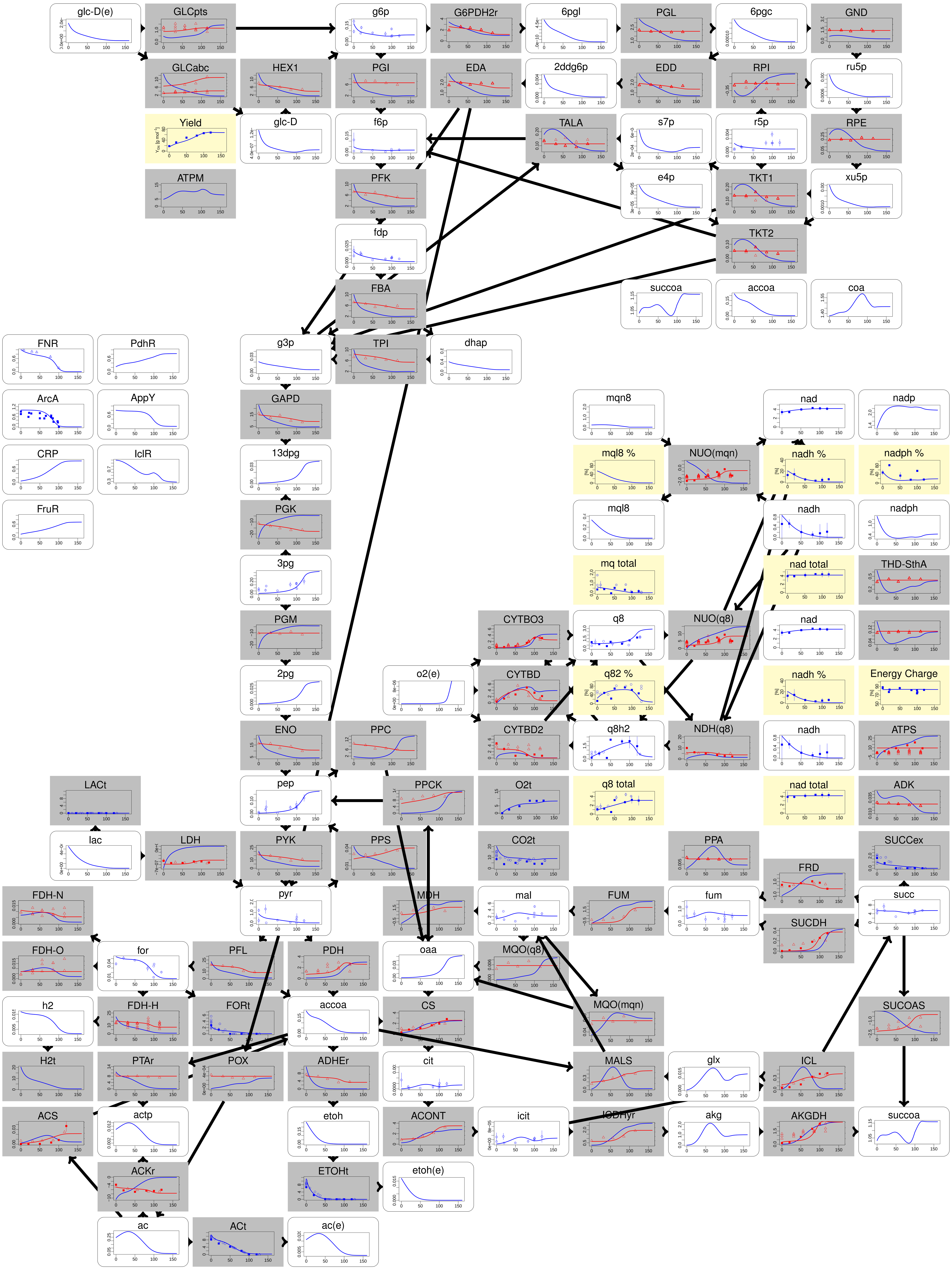

Legend: Steady-state simulation results of the model in comparison to measurement data. The abscissa is aerobiosis in percent.
White boxes represent metabolites. The ordinate is in $\mathrm{nmol/g}$. Blue lines and circles show simulation and measurement data, respectively. The error bars show the technical standard deviation.
Gray boxes represent reactions. The blue lines show the reaction flux in $\mathrm{mmol/gDCW/h}$. Blue symbols show flux data computed from the steady state concentration of extracellular concentrations. Different symbols indicate that the data was measured in different laboratories. The red lines show gene expression in arbitrary units. If two red lines are shown, they refer to different genes with qualitatively different expression. Red triangles show microarray data. If for one reaction several triangles are shown, they refer to different genes. Red squares show RT-PCR data with standard deviation.
The labels of the boxes are explained in Supplementary Data Sheet 1
