# Supplementary material for: A mathematical model of metabolism and regulation provides a systems-level view of how Escherichia coli responds to oxygen
Source: Front Microbiol. 2014 Mar 27;5:124. doi: 10.3389/fmicb.2014.00124 (PMC3973912; doi:10.3389/fmicb.2014.00124)

Steady-state response of the model to enforced precursor effluxes

The abscissas show the biomass-specific production flux J_{prod} in mol/gDCW/h. The blue lines show the productivity per fermenter volume $q_{\text{prod}} = J_{\text{prod}} \cdot c_X$ where c_X is the biomass concentration in g/l.

The black solid lines show the biomass concentration c_X. The black dashed lines show the intracellular concentration of the respective metabolite. The latter two curves are scaled to the undisturbed case and start at unity. The abbreviations for the precursor molecules are explained in Supplementary Data Sheet 1.

Aerobic Conditions

Aeration is fixed at a level that results in 160% aerobiosis of the undisturbed case ($J_{\text{prod}} = 0$).

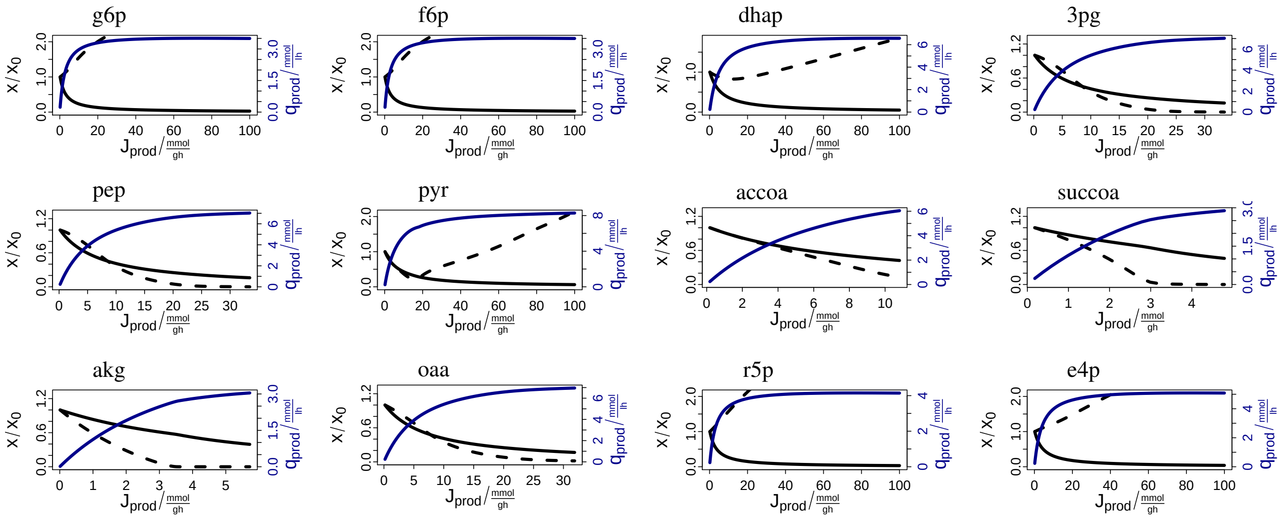

Aneaerobic Conditions

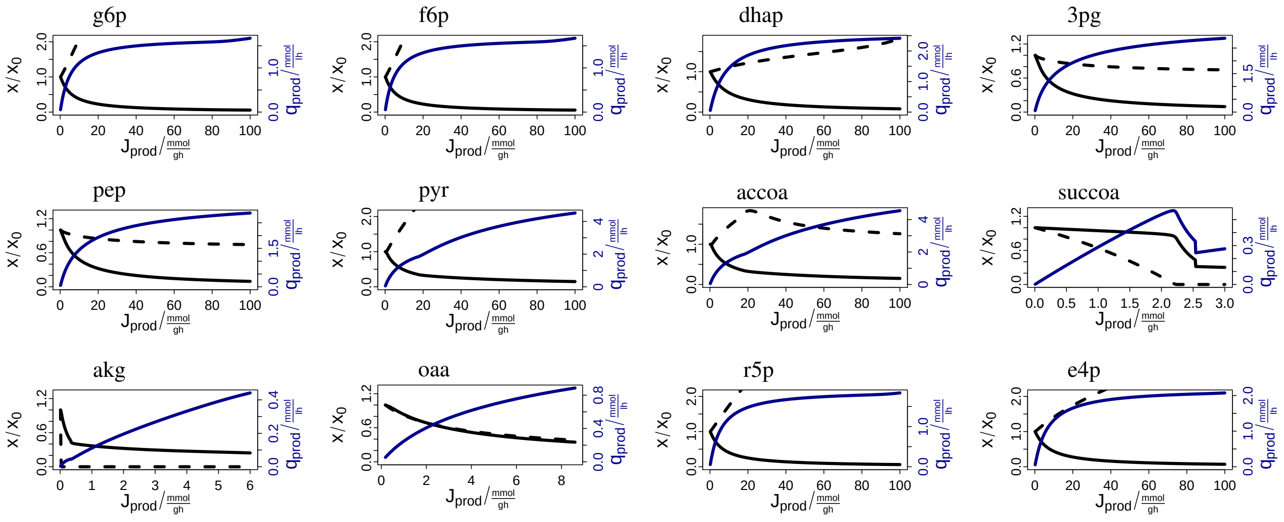

Supplement: Supplementary Data Sheet 5 — Steady State Answer of the Model to enforced precursor effluxes. [file DataSheet5.PDF]
